# Drug-perturbation-based stratification of blood cancer

**DOI:** 10.1172/JCI93801

**Published:** 2017-12-11

**Authors:** Sascha Dietrich, Małgorzata Oleś, Junyan Lu, Leopold Sellner, Simon Anders, Britta Velten, Bian Wu, Jennifer Hüllein, Michelle da Silva Liberio, Tatjana Walther, Lena Wagner, Sophie Rabe, Sonja Ghidelli-Disse, Marcus Bantscheff, Andrzej K. Oleś, Mikołaj Słabicki, Andreas Mock, Christopher C. Oakes, Shihui Wang, Sina Oppermann, Marina Lukas, Vladislav Kim, Martin Sill, Axel Benner, Anna Jauch, Lesley Ann Sutton, Emma Young, Richard Rosenquist, Xiyang Liu, Alexander Jethwa, Kwang Seok Lee, Joe Lewis, Kerstin Putzker, Christoph Lutz, Davide Rossi, Andriy Mokhir, Thomas Oellerich, Katja Zirlik, Marco Herling, Florence Nguyen-Khac, Christoph Plass, Emma Andersson, Satu Mustjoki, Christof von Kalle, Anthony D. Ho, Manfred Hensel, Jan Dürig, Ingo Ringshausen, Marc Zapatka, Wolfgang Huber, Thorsten Zenz

**Affiliations:** 1 European Molecular Biology Laboratory (EMBL), Heidelberg, Germany.; 2 Department of Medicine V, University Hospital Heidelberg, Heidelberg, Germany.; 3 Molecular Therapy in Hematology and Oncology, and Department of Translational Oncology, National Center for Tumor Diseases and German Cancer Research Centre, Heidelberg, Germany.; 4 Molecular Medicine Partnership Unit (MMPU), Heidelberg, Germany.; 5 Cellzome, Heidelberg, Germany.; 6 Division of Hematology, Departments of Internal Medicine and Biomedical Informatics, The Ohio State University, Columbus, Ohio, USA.; 7 Division of Epigenomics and Cancer Risk Factors, German Cancer Research Centre, Heidelberg, Germany.; 8 Division of Biostatistics, German Cancer Research Centre, Heidelberg, Germany.; 9 Institute of Human Genetics, University of Heidelberg, Heidelberg, Germany.; 10 Department of Molecular Medicine and Surgery, Karolinska Institutet, Stockholm, Sweden.; 11 European Molecular Biology Laboratory (EMBL), Chemical Biology Core Facility, Heidelberg, Germany.; 12 Department of Translational Medicine, Amedeo Avogadro University of Eastern Piedmont, Novara, Italy; Division of Hematology, Oncology Institute of Southern Switzerland, Bellinzona, Switzerland.; 13 Friedrich-Alexander-University of Erlangen-Nürnberg, Department of Chemistry and Pharmacy, Organic Chemistry II, Erlangen, Germany.; 14 Hematology/Oncology, Department of Medicine, Johann Wolfgang Goethe University, Frankfurt, Germany; Department of Haematology, Cambridge Institute of Medical Research, University of Cambridge, Cambridge, United Kingdom.; 15 German Consortium for Translational Cancer Research (DKTK), Heidelberg, Germany.; 16 Department of Hematology/Oncology, University Hospital Freiburg, Freiburg, Germany and Tumorzentrum ZeTuP Chur, Chur, Schweiz.; 17 Department of Internal Medicine I, University Hospital Cologne, Cologne, Germany.; 18 INSERM U1138, Université Pierre et Marie Curie-Paris and Service d’Hématologie Biologique, Hôpital Pitié-Salpêtrière, Paris, France.; 19 Hematology Research Unit Helsinki, University of Helsinki, Helsinki, Finland and Department of Hematology, Comprehensive Cancer Centre, Helsinki University Hospital, Helsinki, Finland.; 20 Heidelberg Centre for Personalized Oncology, DKFZ-HIPO, DKFZ, Heidelberg, Germany.; 21 Mannheim Oncology Practice, Mannheim, Germany.; 22 Department of Hematology, University Hospital Essen, Essen, Germany.; 23 Department of Hematology, University of Cambridge, Cambridge, United Kingdom.; 24 Division of Molecular Genetics, German Cancer Research Centre, Heidelberg, Germany.; 25 Department of Hematology, University of Zürich, Zürich, Switzerland.

**Keywords:** Hematology, Oncology, B cell receptor, Drug screens, Leukemias

## Abstract

As new generations of targeted therapies emerge and tumor genome sequencing discovers increasingly comprehensive mutation repertoires, the functional relationships of mutations to tumor phenotypes remain largely unknown. Here, we measured ex vivo sensitivity of 246 blood cancers to 63 drugs alongside genome, transcriptome, and DNA methylome analysis to understand determinants of drug response. We assembled a primary blood cancer cell encyclopedia data set that revealed disease-specific sensitivities for each cancer. Within chronic lymphocytic leukemia (CLL), responses to 62% of drugs were associated with 2 or more mutations, and linked the B cell receptor (BCR) pathway to trisomy 12, an important driver of CLL. Based on drug responses, the disease could be organized into phenotypic subgroups characterized by exploitable dependencies on BCR, mTOR, or MEK signaling and associated with mutations, gene expression, and DNA methylation. Fourteen percent of CLLs were driven by mTOR signaling in a non–BCR-dependent manner. Multivariate modeling revealed immunoglobulin heavy chain variable gene (IGHV) mutation status and trisomy 12 as the most important modulators of response to kinase inhibitors in CLL. Ex vivo drug responses were associated with outcome. This study overcomes the perception that most mutations do not influence drug response of cancer, and points to an updated approach to understanding tumor biology, with implications for biomarker discovery and cancer care.

## Introduction

The clinical response to anticancer agents is heterogeneous, which is a major barrier to effective cancer care. Being able to more accurately predict response before choice of treatment would improve response rates, reduce unnecessary treatments, and be more economical. However, predicting patient response to drugs is not reliable for most cancers, owing to a lack of predictive biomarkers and our incomplete understanding of the mechanisms underlying response heterogeneity (1, 2).

Determinants of drug response have been investigated in immortalized cancer cell lines (http://www.cancerRxgene.org, ref. 3; http://www.broadinstitute.org/ccle, ref. 4; http://www.broadinstitute.org/ctrp, ref. 5), and recent technology improvements have increased throughput (6) and used near-complete genetic profiles (7). However, the properties of disease cells and the heterogeneity of a disease can only be partially captured using cell line panels. An ideal platform to query mechanisms underlying variable drug response will directly interrogate primary cancer cells of individual patients. Clonal selection is reduced by short-term culture of primary cells, and the true genetic and phenotypic diversity of a disease is represented by a large, representative cohort of patient-derived samples. Rare mutations (from the long tail of the mutation distribution in the disease) are included, which enables uncovering determinants of drug response that might be missed using current methods. A unique feature of such direct use of patient cells is the potential to derive individualized therapeutic options for the donating patients (8–11) and the ability to pursue sensitivity signals clinically (12). Indeed, several studies have yielded novel genetic markers and drug repurposing opportunities based on individual patient observations (13–15).

Targeted treatments have revolutionized care of some blood cancers (16–19). While a new generation of targeted drugs is emerging for leukemia and lymphoma (20–23), surprisingly little use has been made of molecular information for therapeutic stratification (24, 25). This is in part due to shortcomings of traditional biomarker discovery in clinical trials, where throughput is limited in both drug number and sample size. Here we propose that by mapping the distinctive signaling pathway dependencies and drug sensitivity patterns of individual cancers in parallel, it is possible to discover genotype-phenotype associations and underlying molecular mechanisms in a more rapid and systematic fashion, and thus to better support precision medicine stratification. We report a large-scale study of drug sensitivities of primary leukemia and lymphoma that links drug responses to genotypes and molecular processes involved in disease pathogenesis.

## Results

### Mapping drug sensitivity of primary leukemia and lymphoma cells.

**Figure 1:**
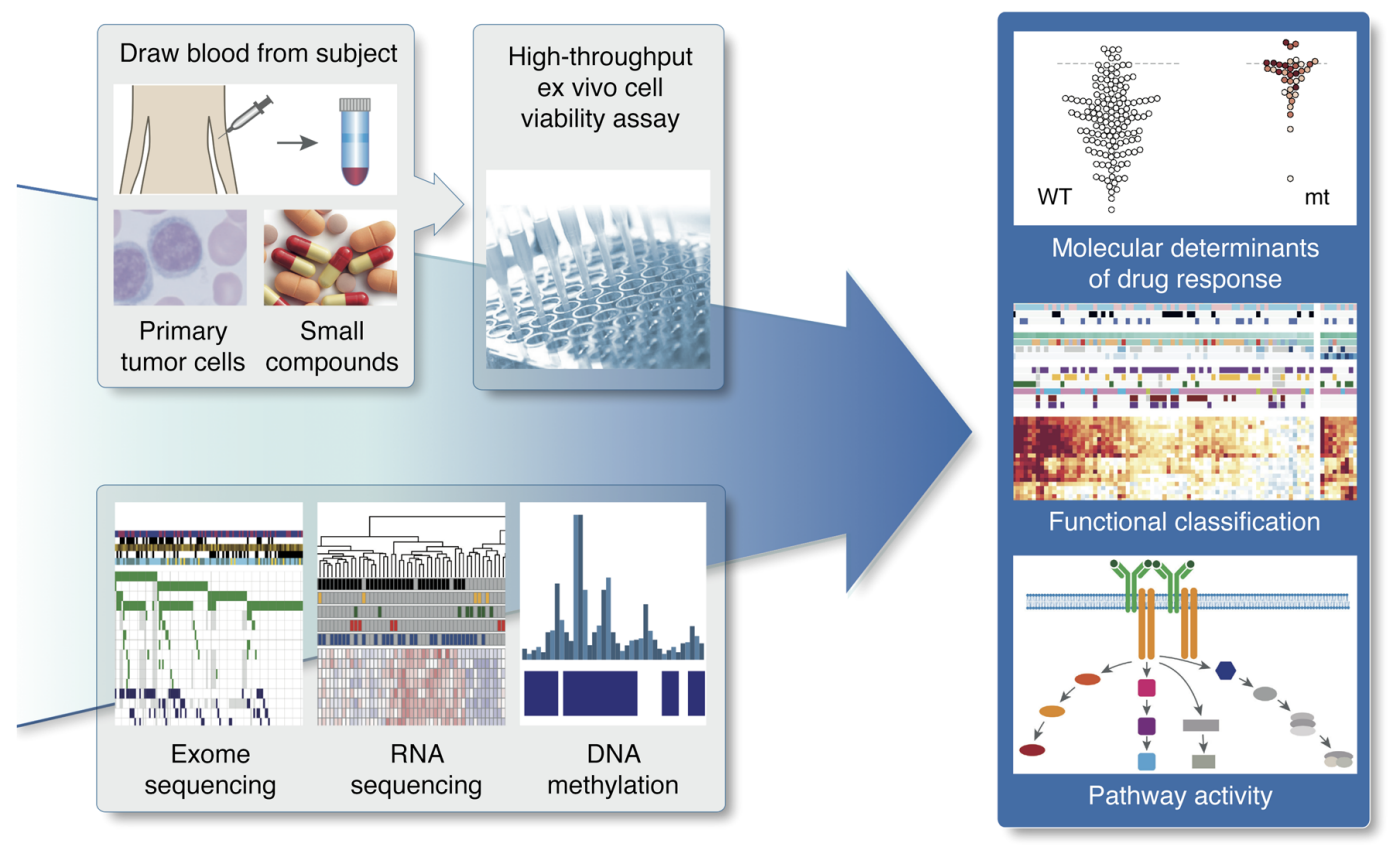
Study outline. By combining functional drug response screening with omics profiling, we systematically queried drug response phenotypes, underlying molecular predictors, and pathway dependencies of leukemia and lymphoma. mt, mutant.

**Figure 2:**
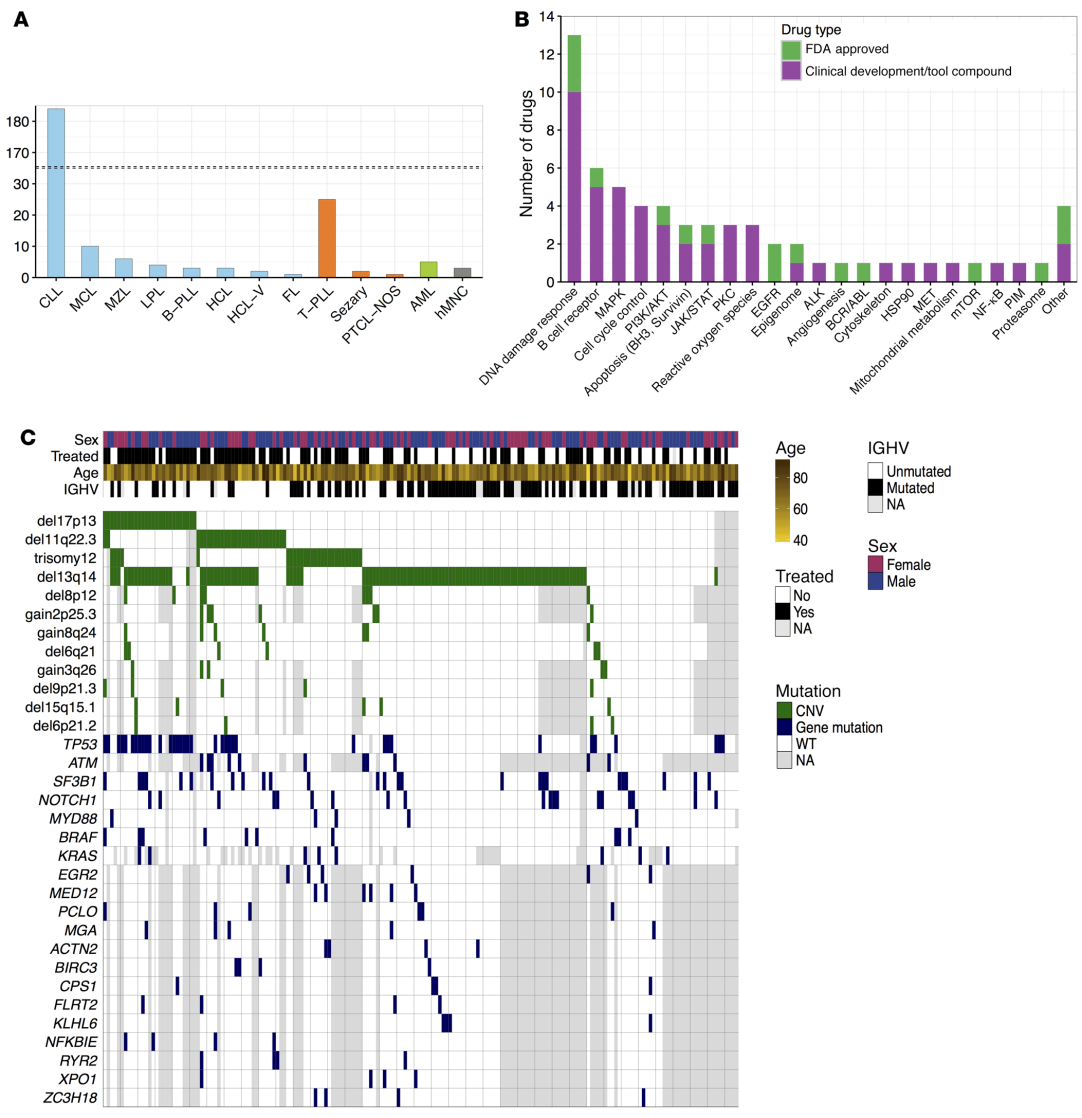
Overview of sample cohort and drugs. (**A**) Pathology classification of samples. The largest groups were chronic lymphocytic leukemia (CLL; *n* = 184), T cell prolymphocytic leukemia (T-PLL; *n* = 25), and mantle cell lymphoma (MCL; *n* = 10). Color indicates cell lineage: B cell (blue), T cell (orange), myeloid (green), and normal blood cells (gray). The dashed line indicates a scale break. (**B**) Compounds tallied by their targets. Green indicates FDA-approved drugs and purple indicates drugs that are tool compounds or in clinical development. (**C**) The genetic landscape of our CLL cohort (*n* = 184), including recurrent copy number variations (CNVs, green) and somatic mutations (blue); instances of missing data are shown in gray. Previously reported associations include the frequent co-occurrence of del17p13 and *TP53* mutation (Fisher test: *P* = 10^–11^, odds ratio = 29), del11q22 and *ATM* mutation (Fisher test: *P* = 0.05, odds ratio = 3.7). In addition, we detected a mutual exclusivity pattern between del13q14 and trisomy 12 (Fisher test: *P* = 0.0006, odds ratio = 0.2). ALK, anaplastic lymphoma kinase; FL, follicular lymphoma; HCL-V, hairy cell leukemia variant; hMNC, human mononuclear cell; LPL, lymphoplasmacytic lymphoma; NA, not available; PTCL-NOS, peripheral T cell lymphoma not otherwise specified.

We measured the effect of drugs used clinically or targeting pathways important in cancer on the viability of primary leukemia and lymphoma samples of B cell, T cell, and myeloid origin ex vivo (Figures 1 and 2, and Supplemental Tables 1–3; supplemental material available online with this article; https://doi.org/10.1172/JCI93801DS1). We used a T-shaped experimental design in order to cover heterogeneity of responses widely, among 12 diseases, and deeply (184 samples) within 1 disease, chronic lymphocytic leukemia (CLL).

To query molecular determinants of drug response, we used targeted sequencing and whole-exome sequencing (WES) (Supplemental Table 4) for paired tumor and normal samples, mapping of structural variants, genome-wide DNA methylation profiles (450k/850k microarrays), and RNA sequencing (RNA-Seq), and assembled the Primary Blood Cancer Cell Encyclopedia (PACE).

**Figure 3:**
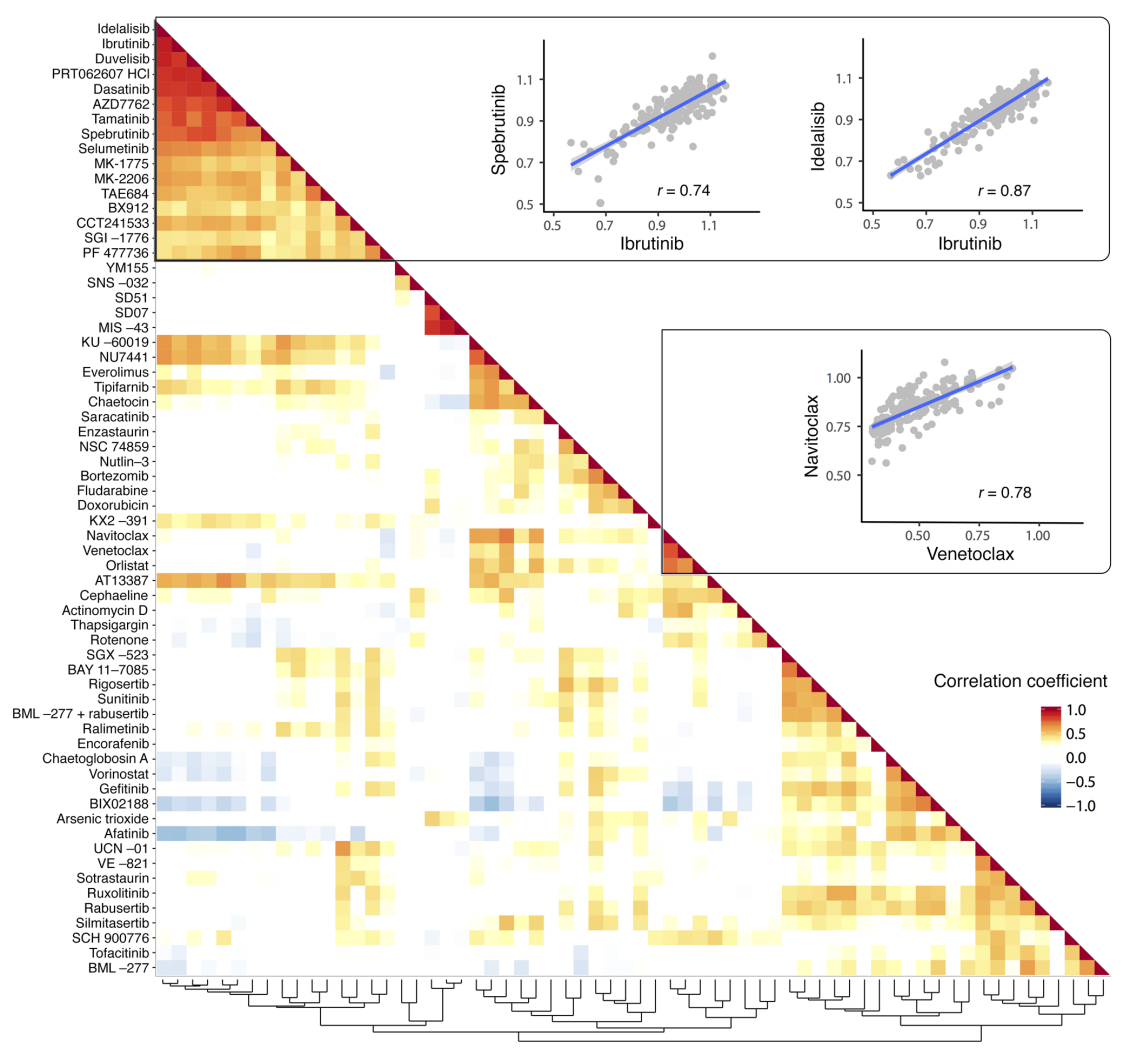
Drug profile similarities reflect mode of action. “Guilt by association” prediction of drug targets and mechanism of action. For each pair of drugs used in the screen, the Pearson correlation coefficient (*r*) was computed from the viabilities of the 184 CLL samples after drug treatment (average of the 2 lowest concentrations). The rows and columns of the resulting drug-drug correlation matrix were arranged based on the hierarchical clustering shown at the bottom, and the matrix is displayed as a heatmap. The major blocks are (i) kinase inhibitors targeting the B cell receptor, including idelalisib (PI3K), ibrutinib (BTK), duvelisib (PI3K), PRT062607 (SYK); (ii) inhibitors of redox signaling/reactive oxygen species (ROS) (MIS−43, SD07, SD51); and (iii) BH3 mimetics (navitoclax, venetoclax). The scatter plots show 3 instances of pairwise correlation analyses of drugs.

We profiled 246 patient and 3 control samples with 64 drugs (data of 63 used after data quality control) in series of 5 concentrations, which resulted in a drug response matrix of 79,680 measurements. Similar to clinical response heterogeneity, drugs showed a heterogeneous spectrum of responses across samples (Supplemental Figure 1). We began the data analysis by clustering the drugs based on the similarity of their response profiles across CLL samples (Figure 3). The clustering gave a detailed reflection of drug target identity or relatedness. For instance, the responses to inhibitors targeting the B cell receptor (BCR) components Bruton’s tyrosine kinase (BTK), phosphatidylinositol 3-kinase (PI3K), and spleen tyrosine kinase (SYK) were highly correlated across the 184 CLL samples and showed a distinctive profile, which was shared with inhibitors of kinases downstream of the BCR, including AKT, LYN, and SRC. A BCR-like profile was also elicited by nominally unrelated drugs including AZD7762, PF477736 (targeting checkpoint kinase, CHEK), and AT13387 (targeting heat shock protein, HSP90).

**Figure 4:**
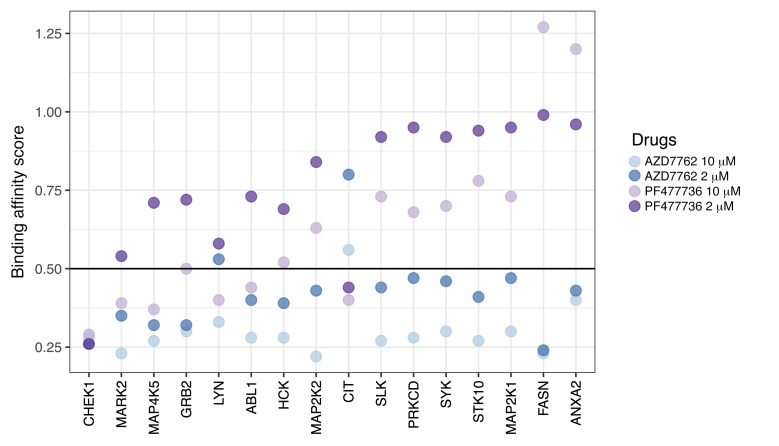
Target profiling of AZD7762 and PF477736. Binding affinity scores were determined proteome-wide using the kinobead assay (26); lower scores indicate stronger physical binding. Here, the data are shown for those proteins that had a score less than 0.5 in at least one assay, and that were previously identified as responders to B cell receptor stimulation with anti-IgM in B cell lines (27).

To understand the unexpected activity of AZD7762 and PF477736, we used the kinobeads assay, which quantifies drug-protein binding affinity through competition with immobilized nonselective binders and proteome-wide quantitative mass spectrometry (26). In addition to CHEK1/2 kinases, both drugs targeted dozens of other proteins (Supplemental Table 5). We intersected these target lists with proteins that were identified as BCR effectors based on their BCR-dependent phosphorylation in lymphoma cell lines after BCR engagement (27). This intersection yielded 16 proteins, including well-known BCR pathway members (Figure 4). We then tested the effect of AZD7762 on proximal BCR signaling by measuring anti–IgM-induced calcium release in HBL2 and BL60 cell lines, commonly used models for lymphoma. Similar to ibrutinib, AZD7762 blocked anti–IgM-induced calcium mobilization (Supplemental Figure 2). To characterize the drug’s effects further downstream of the BCR, we assayed the activation of signaling targets using phospho-specific antibodies in a lymphoma cell line (HBL-2) and 5 primary CLL samples. Upon AZD7762 exposure, we observed consistent downregulation of p-AKT, p-BTK, and p-SYK, but not p-S6 (mTOR) (Supplemental Figure 3), and induction of apoptosis (Supplemental Figure 4). AZD7762 caused stronger viability effects in unmutated CLL (U-CLL) samples, which we confirmed under conditions of coculture with stroma cells (Supplemental Figure 4).

To follow up on the AT13387 result, we investigated 2 additional HSP90 inhibitors, ganetespib and onalespib, in 120 of the CLL samples. Consistent with our data for AT13387, these drugs had higher activity in U-CLL than in M-CLL (Supplemental Figure 5). HSP90 inhibitors are known to attenuate BCR and nuclear factor-κB (NF-κB) signaling (28), and our findings are in line with a report that AT13387 compromises the activity of the pivotal BCR-proximal effector SYK, which was identified as an HSP90 client protein (29).

Together, these results show that the similarity of response profiles across a large set of patient samples accurately assigns drugs into groups of similar mechanisms of action. In line with this concept, the phenotypic clustering of drugs depended on the sample selection; when we performed the same analysis on the T cell prolymphocytic leukemia (T-PLL) samples, the cluster of BCR-targeting drugs largely dissolved, while other clusters (reactive oxygen species [ROS], Bcl-2 homology domain 3, BH3 mimetics) — less dependent on disease-specific activity — remained (Supplemental Figure 6). We conclude that the drug perturbations acted as specific molecular probes for the tumor cells’ survival dependencies and that drug response profiles allow “guilt by association”–based mapping of drug targets. They enable the discovery of unexpected targets, as demonstrated by the targeting of the BCR signaling cascade by molecules originally designed to inhibit HSP90 or CHEK.

### Disease-specific drug sensitivity phenotypes.

**Figure 5:**
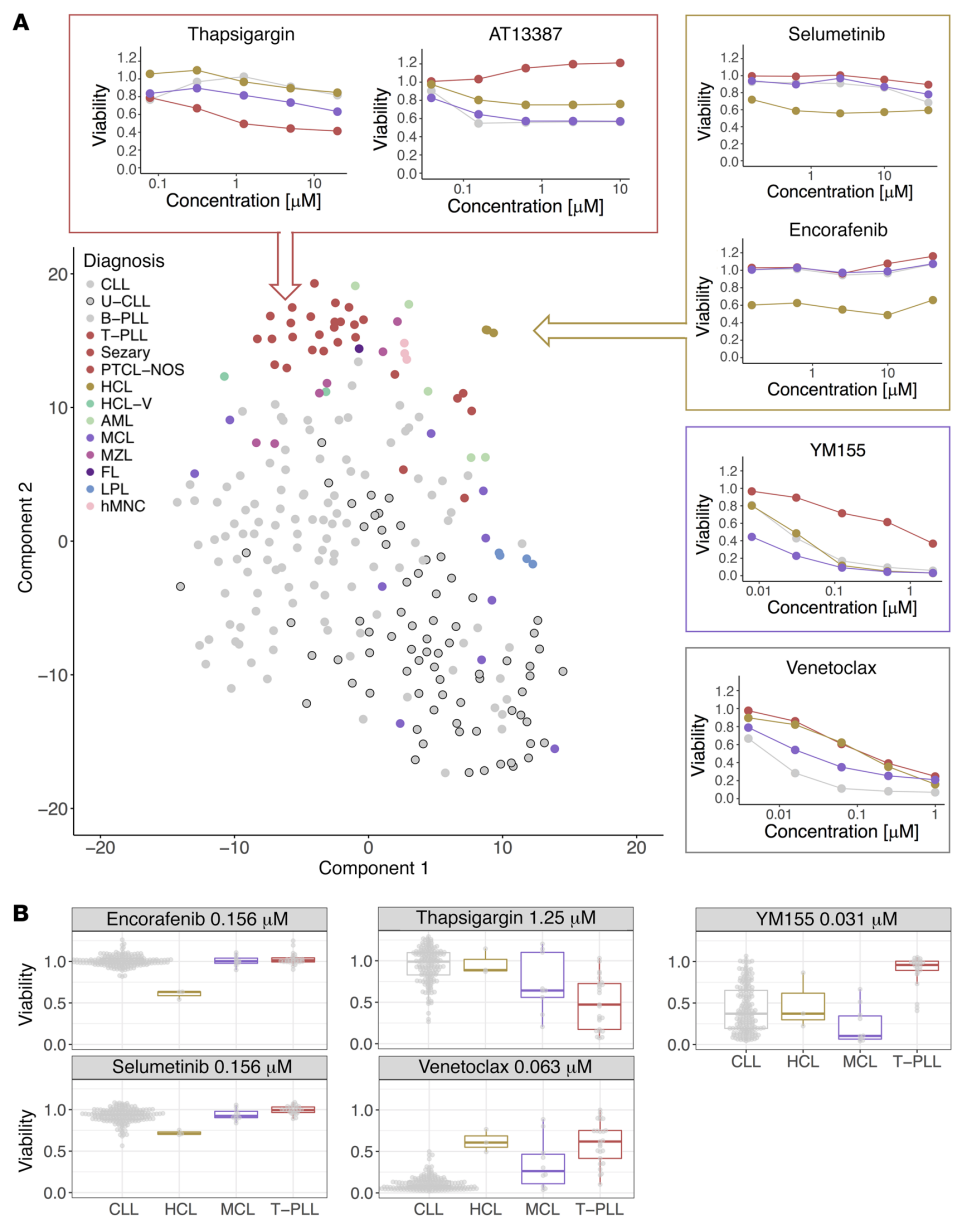
Disease-specific drug response phenotypes of blood cancers. (**A**) *t*-distributed stochastic neighbor embedding (*t*-SNE), a machine learning algorithm for dimensionality reduction, was used to visualize similarities among 246 patient samples with respect to the 315 drug sensitivity measurements (each of 63 drugs at 5 concentrations). The plot shows a distinctive separation of pathologic disease entities based on their drug sensitivity pattern. The line plots show mean viabilities for individual disease entities (CLL, gray; HCL, yellow; MCL, purple; and T-PLL, brown) and drugs across 5 concentrations, highlighting disease-specific differences. (**B**) Primary data for individual drugs provide examples for disease-specific response and sample variation (CLL, *n* = 184; HCL, *n* = 3; MCL, *n* = 10; T-PLL, *n* = 25). FL, follicular lymphoma; HCL-V, hairy cell leukemia variant; hMNC, human mononuclear cell; LPL, lymphoplasmacytic lymphoma PTCL-NOS, peripheral T cell lymphoma not otherwise specified.

To gain a global overview of drug response patterns across all patients, we employed *t*-distributed stochastic neighbor embedding (*t*-SNE), a machine learning algorithm for visualizing a set of objects in a 2-dimensional plane. This unsupervised analysis partitioned different disease types (i.e., T cell lymphomas, HCL, lymphoplasmacytic lymphoma [LPL], CLL) and the healthy mononuclear cells into distinct clusters based on their drug sensitivities (Figure 5A). This finding indicates that drug responses depend on disease, reflecting underlying cell lineages, differentiation states, and pathway activities. To further dissect the influence of disease on drug response and to identify disease-specific vulnerabilities, we compared each drug’s viability effects across diseases (Figure 5B and Supplemental Figure 7). T-PLL was not responsive to BCR inhibitors, as expected, but was also more resistant to other drugs, including apoptosis-inducing BH3 mimetics and AT13387. In contrast, T-PLL was most sensitive to thapsigargin, a noncompetitive inhibitor of the sarco-/endoplasmic reticulum Ca^2+^ ATPase (SERCA), and to JAK inhibitors (*P* < 0.001), revealing repurposing opportunities for these drugs, some of which are already in clinical use. In CLL, BH3 mimetics and BCR inhibitors showed disease-specific activity, similar to clinical observations. Acute myeloid leukemia (AML) was sensitive to tamatinib (targeting SYK) and tipifarnib (targeting farnesyl protein transferase), and marginal zone lymphoma (MZL) was resistant to BCR inhibitors and other kinase inhibitors, a result consistent with prevalent reliance of MZL on NF-κB–activating mutations (30). Mantle cell lymphoma (MCL) was preferentially sensitive to YM155 (*P* < 0.001), a cytotoxic agent with unclear mechanism of action reported to target survivin, Mcl-1 (31), and PI3K signaling (32). Mirroring clinical observations, subsets of MCL samples were sensitive to BCR inhibitors or the mTOR inhibitor everolimus (23, 33). Hairy cell leukemia (HCL), which commonly carries the BRAF V600E mutation (34), was distinctly responsive to BRAF and MEK inhibition.

These results validate our experimental approach, as they show how the clinical response of diseases is recapitulated. Moreover, they demonstrate that fine-grained classification of disease is possible based on drug response phenotypes, and how new disease-specific drug sensitivities with potential clinical exploitation can be uncovered.

### Drug-perturbation-based functional classification of CLL.

**Figure 6:**
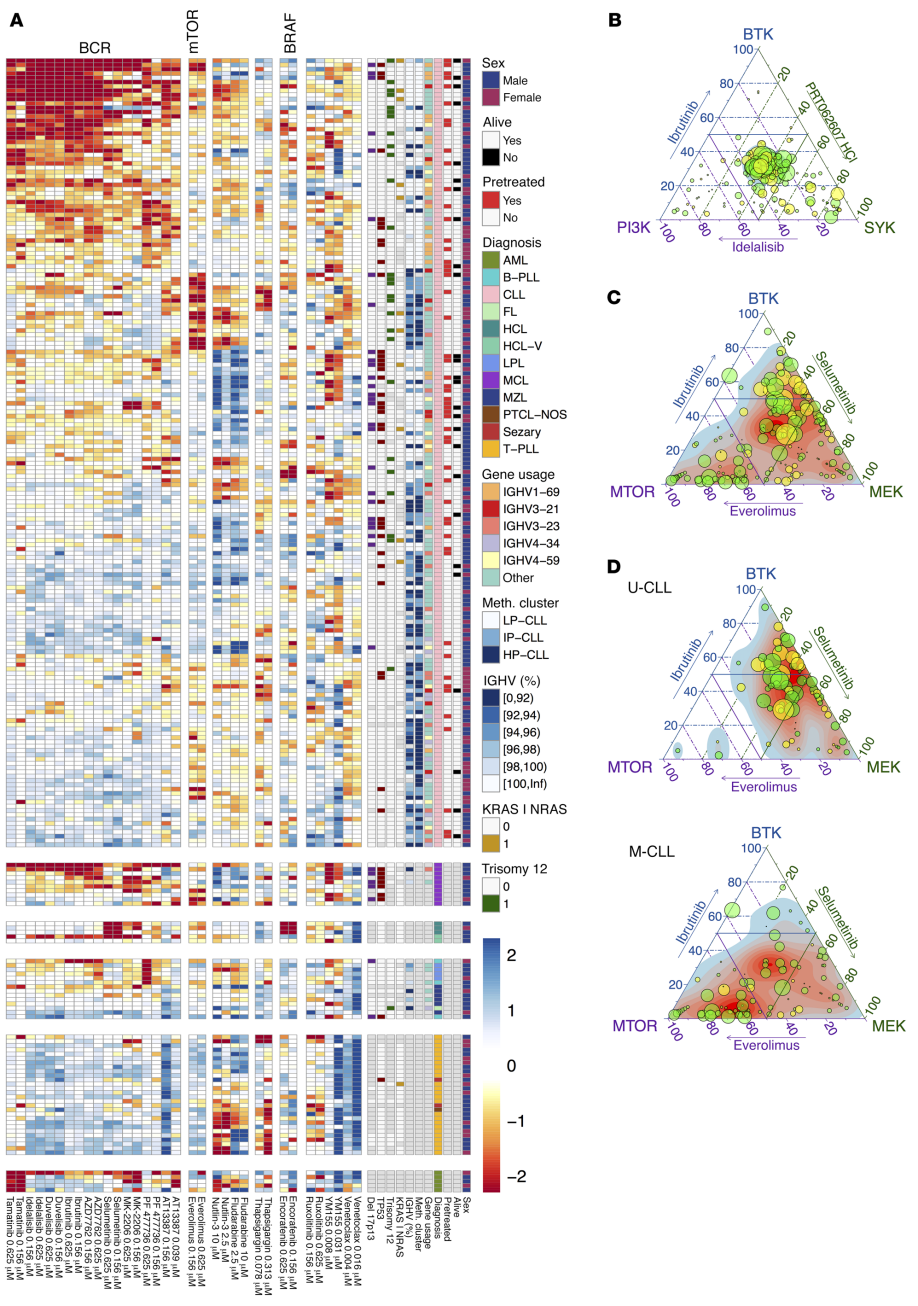
Functional classification of blood cancer based on drug perturbations. (**A**) A global overview of the drug response landscape reveals heterogeneity within diseases and functionally defined disease subgroups. The heatmap matrix shows the viability measurements for 246 samples (rows) and 17 of the drugs at 2 concentrations each (columns). The data are shown on a *Z*-score scale, i.e., centered and scaled within each column. The color bars to the right show sample annotations. Prior to clustering, samples were divided into 6 disease groups, indicated by the horizontal gaps. A more detailed version of this plot is available in Supplemental Figure 8. (**B**) Relative effects of ibrutinib (BTK), idelalisib (PI3K), and PRT062607 (SYK) on each of the 184 CLL samples are shown in ternary plots. Given percentage viability values (*v_i_*) of 3 drugs compared, the relative effect of drug *i* is measured by (100 – *v_i_*)/(300 – [*v*_1_ + *v*_2_ + *v*_3_]), for *i* = 1, 2, and 3. Numbers per sample add up to 1 and correspond to positions within an equilateral triangle. The maximum of 100 – *v_i_*, as a measure of the overall susceptibility of the sample, is shown by dot size. Each drug is represented by the average of the 2 lowest concentrations. Response to the BCR inhibitors was similar in the majority of CLL samples. Prior treatment is indicated by dot color (green: pretreated, *n* = 52; yellow: untreated, *n* = 132). (**C**) In contrast, comparison of relative responses to ibrutinib, selumetinib, and everolimus revealed a heterogeneous response. (**D**) Same data as in panel **C**, but separately plotted for U- and M-CLL (*n* = 74 and *n* = 98, respectively). U-CLL showed predominant reliance on BTK and MEK signaling, whereas M-CLL showed a less BTK-dependent response pattern, with many cases of predominant MEK or mTOR sensitivity. FL, follicular lymphoma; HCL-V, hairy cell leukemia variant; hMNC, human mononuclear cell; LPL, lymphoplasmacytic lymphoma PTCL-NOS, peripheral T cell lymphoma not otherwise specified.

To gain a global overview of drug response patterns across patients we clustered tumors and drugs by response (Figure 6A and Supplemental Figure 8). We considered each concentration separately, in a model-free approach that allows for dose-dependent target specificity. Within CLL, response to BCR inhibitors formed a dominant and continuous gradient that separated the samples by their immunoglobulin heavy chain variable (IGHV) region mutation status. IGHV status (U-CLL or mutated [M-CLL]) reflects the cellular differentiation stage of the tumor-initiating cell and provides a key biological subdivision of CLL with major clinical implications (35). Our finding is consistent with the increased BCR signaling capacity in U-CLL (36) and shows the ability of drug-based screening to probe key survival pathways. Within this dominant gradient driven by BCR dependence, we discovered a group of patients with M-CLL that were sensitive to everolimus, an mTOR inhibitor. Moreover, comparison of the responses revealed that a subset of these rely on mTOR signaling activity independent of BCR signaling. A similar organization, with a gradient of BCR activity and a subgroup with BCR-independent mTOR activity, appeared in MCL, a related B cell lymphoma. Clinical studies demonstrated strong activity of the BTK inhibitor ibrutinib and the mTOR inhibitor temsirolimus in subsets of MCL (23, 37), and our finding reveals potential biomarkers for treatment. Altogether, the patterns that we observed based on unsupervised analysis suggest that CLL can be subdivided into functional disease categories based on drug response, which is in line with CLL being able to arise from multiple driver pathways that can be activated to different degrees. Moreover, they suggest that there is a limited repertoire of such constellations and that most tumors fall into a finite number of signature clusters.

### A model of phenotypic heterogeneity of CLL.

To further dissect signaling activities and survival dependencies in CLL, we selected drugs that probe specific molecular pathways. First, we compared 3 drugs that inhibit BCR signaling targets: ibrutinib (BTK), idelalisib (PI3K), and PRT062607 (SYK). These 3 targets are key components of proximal BCR signaling. While the responses of CLL samples to these agents ex vivo were variable across tumors, they were similar across the 3 drugs (Figure 6B). Next, we selected drugs that showed more differences between each other and compared dependency on BCR (BTK, SYK, PI3K) with MEK (selumetinib) and mTOR (everolimus) (Figure 6C and Supplemental Figure 9, A and B). The spread-out distribution of the samples reflects a heterogeneous response of CLLs to inhibition of these signaling components. To dissect this distribution, we stratified the analysis between U- and M-CLL. We found that U-CLLs are relatively homogeneous and predominantly rely on BTK and MEK signaling, consistent with MEK/ERK activation downstream of the BCR (Figure 6D). In contrast, M-CLLs showed a more heterogeneous organization, with BTK-independent response groups characterized by mTOR sensitivity. To further explore the relationships between responses to inhibition of BCR, mTOR, and MEK in CLL, we exposed primary CLL samples (6 M-CLL and 6 U-CLL) to ibrutinib, idelalisib, selumetinib, and everolimus and compared the drug-induced gene-expression changes (Supplemental Figure 9C). While the tumors’ transcriptional responses to ibrutinib and idelalisib were similar, larger differences existed between each tumor’s response to BCR inhibitors, selumetinib (MEK) and everolimus (mTOR). This finding implies that CLL survival signaling can be mediated by the BCR as well as by BCR-independent pathways through mTOR and/or MEK.

**Figure 7:**
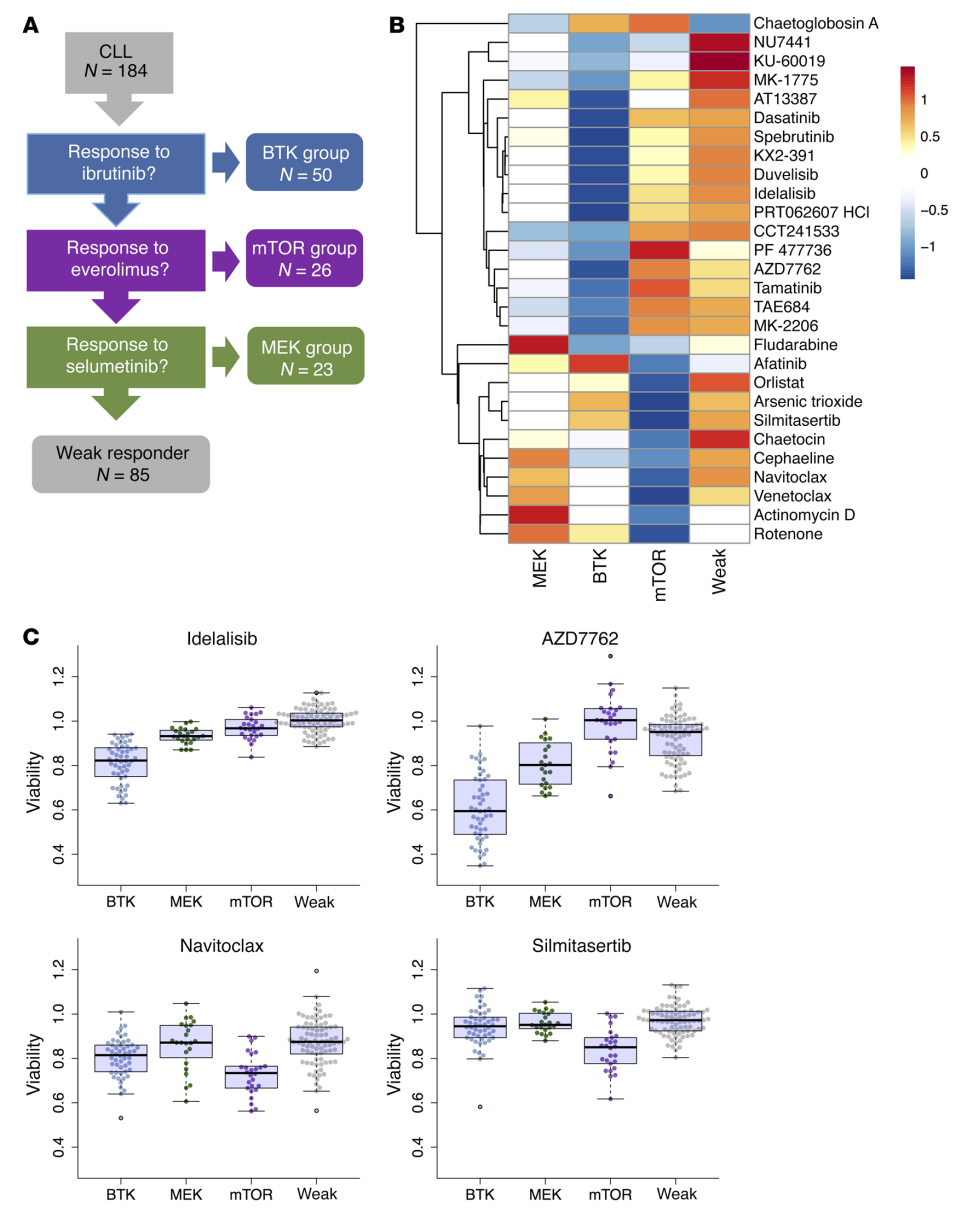
Hierarchical model of drug response phenotypes in CLL. (**A**) We derived a decision tree model that classifies CLL patients into 4 drug-response-based groups. First, we asked if ibrutinib caused strong viability effects (BTK group *n* = 50/184), second, whether the remaining patient samples responded to everolimus (mTOR group *n* = 26/184) and third, whether they responded to selumetinib (*n* = 23/184). The remaining patients were classified as weak responders (*n* = 85/184) (Supplemental Figure 10). (**B**) Summary of cosensitivities for the 4 groups. We compared drug responses of each group to all samples from the remaining groups (average of the 2 lowest concentrations) using Student’s *t* test. Significant differences (FDR = 5%) with a mean effect size greater than 5% are shown. The heatmap visualizes mean viabilities, row-centered and scaled to zero mean and unit standard deviation. (**C**) Exemplary plots of individual sample data for 4 of the drugs shown in panel **B**. The BH3 mimetic navitoclax and the CK2 inhibitor silmitasertib had stronger viability effects in the mTOR group. AZD7762 and idelalisib had stronger viability effects in the BTK group.

Based on these results, we devised a simple classification tree with binary thresholds of response (Figure 7A and Supplemental Figure 10). It stratifies CLL based on response to ibrutinib (BTK group *n* = 50/184), response to the mTOR inhibitor everolimus but not to inhibition of upstream BTK (mTOR group *n* = 26/184), response to the MEK inhibitor selumetinib, but not to ibrutinib or everolimus (MEK group *n* = 23/184), and a group with weak response (*n* = 85/184). Although defined based on these 3 reference drugs, the BTK and mTOR groups showed coordinated differences in their responses to other drugs as well (Figure 7B). The BTK group was consistently more responsive to other BCR inhibitors (idelalisib [PI3K], spebrutinib [BTK], duvelisib [PI3K], and PRT062607 [SYK]) and multiple other kinase inhibitors (e.g., ATM, DNA-PK, CHEK). Notably, the mTOR group exhibited increased sensitivity to the casein kinase 2 (CK2) inhibitor silmitasertib (Figure 7C). This unanticipated cosensitivity is in line with a recent report of a biological link between CK2 and mTOR activity (38). The mTOR group also exhibits specific sensitivity towards venetoclax and navitoclax (Figure 7C), both inhibitors of the antiapoptotic protein BCL-2.

### Phenotypic subgroups have distinct molecular characteristics.

We next asked whether this drug response phenotype–based classification of patients was associated with distinct molecular profiles or clinical outcomes. Patients in the mTOR group had a longer time to treatment (TTT) compared with the MEK and BTK groups (Supplemental Figure 11A; *P* = 0.04). At the genetic level, we found trisomy 12 to be enriched in the BTK and mTOR groups and absent in weak responders (Supplemental Figure 11B). Trisomy 12 is a structural variant of poorly understood molecular function that occurs in 15%–20% of CLL patients and is associated with a higher incidence of aggressive transformation (39). In contrast, the most frequent alteration in CLL, del13q14, was enriched in weak responders but depleted in the BTK group. Del13q14 involves the putative pathogenic disease loci DLEU2 and microRNA cluster MIR15A–MIR16-1 (39), and our finding might provide further leads towards functional annotation of this deletion. The mTOR group consisted almost exclusively (22 of 23) of M-CLL and comprised 3 of the 4 cases with mutations in *KLHL6*. Deleterious mutations of *KLHL6* are involved in B lymphocyte antigen receptor signaling (40). These results indicate that intrinsic molecular differences underlie the phenotypic response groups.

At the level of gene expression, we searched for genes differentially expressed between the groups (Supplemental Figure 11C). We applied gene set enrichment analysis (Supplemental Figures 12 and 13) and detected enrichment of gene sets known to be associated with increased polycomb repressive complex 1 (PRC1) activity (41), TNF-α stimulation, and IL-2 for the mTOR group. IL-2 induces survival signals in CLL (42) and T cells (43) through p-70S6 and mTOR activity. As a functional link has been reported between active IL-2 signaling and higher IL-10 expression in CLL (44), we investigated expression of several cytokines that are important for CLL survival or characteristic of distinct B cell subsets (45) (Supplemental Figure 11D). Of these, we found only IL-10 to be upregulated in the mTOR group. Moreover, within the mTOR group, IL-10 expression was correlated with better response to everolimus (*P* = 0.03, Supplemental Figure 14). Increased expression of IL-10 is a property of regulatory B cells (B10 cells) (46), and our result might be related to the recent discovery of a subset of M-CLL that shows a B10-like phenotype associated with BCR anergy (47). To further dissect the roles of cytokines, we exposed primary CLL cells (*n* = 16) to different concentrations of cytokines (IL-2, -4, -10, and -21), LPS, and anti-IgM. IL-4, -10, and -21 had prosurvival effects on most samples. However, an effect of IL-10 stimulation was markedly absent in the samples from the mTOR group, possibly due to already high endogenous levels (Supplemental Figure 15).

**Figure 8:**
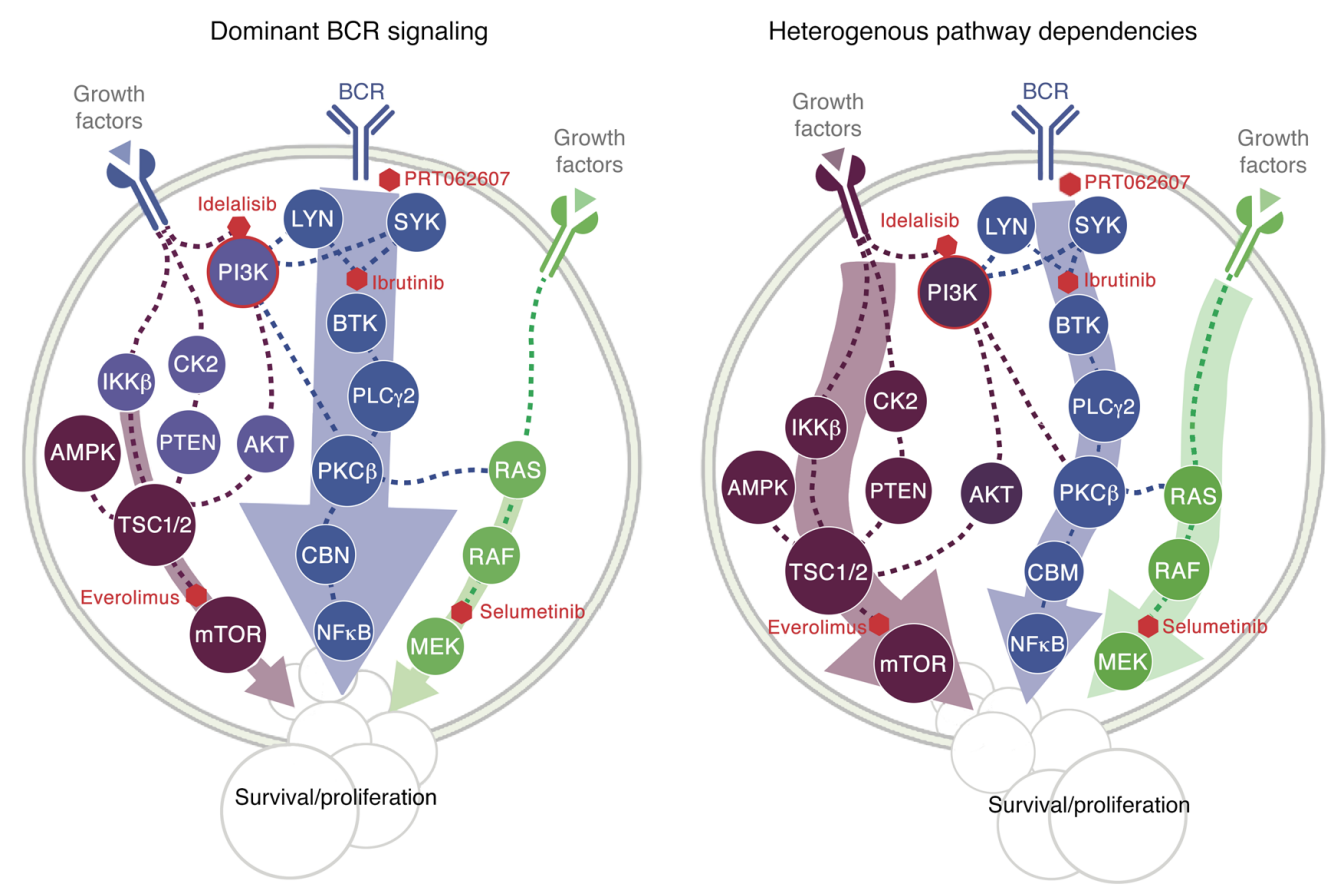
A model for the roles of BCR, mTOR, and MEK pathway activities in CLL. BCR-dependent cases are highly sensitive to inhibition of SYK, BTK, and PI3K. MEK and mTOR activation occur downstream of BCR. Most U-CLL patients belong to this group. In contrast, there is a group of CLL where cells receive survival signals from alternative sources (e.g., cytokines/chemokines) and whose drug response pattern is inconsistent with canonical BCR signaling, as the effect of inhibiting mTOR is greater than for BTK. PI3K, phosphatidylinositol 3-kinase; IKKβ, IκBα-Kinase-complexes; AMPK, AMP-activated protein kinase; TSC1/2, hamartin/tuberin; PDK1, pyruvate dehydrogenase kinase 1; SGK3, serine/threonine-protein kinase; PLC, phosphoinositide-phospholipase C; PKCβ, protein kinase C β; CBM, CARMA1-Bcl10-MALT1 complex; mTOR, mechanistic target of rapamycin; SYK,spleen tyrosine kinase; BTK, Bruton’s tyrosine kinase; Lyn, tyrosine kinase Lyn.

Together, these findings reveal unacknowledged heterogeneity of signaling dependencies in CLL. We summarize them in the signaling model shown in Figure 8. The majority of U-CLL cases depend on dominant, canonical BCR signaling. In contrast, a subset of M-CLLs show BCR-independent signaling mediated through mTOR, which can act downstream of cytokines or chemokines.

### Comprehensive survey of molecular determinants of response in CLL.

**Figure 9:**
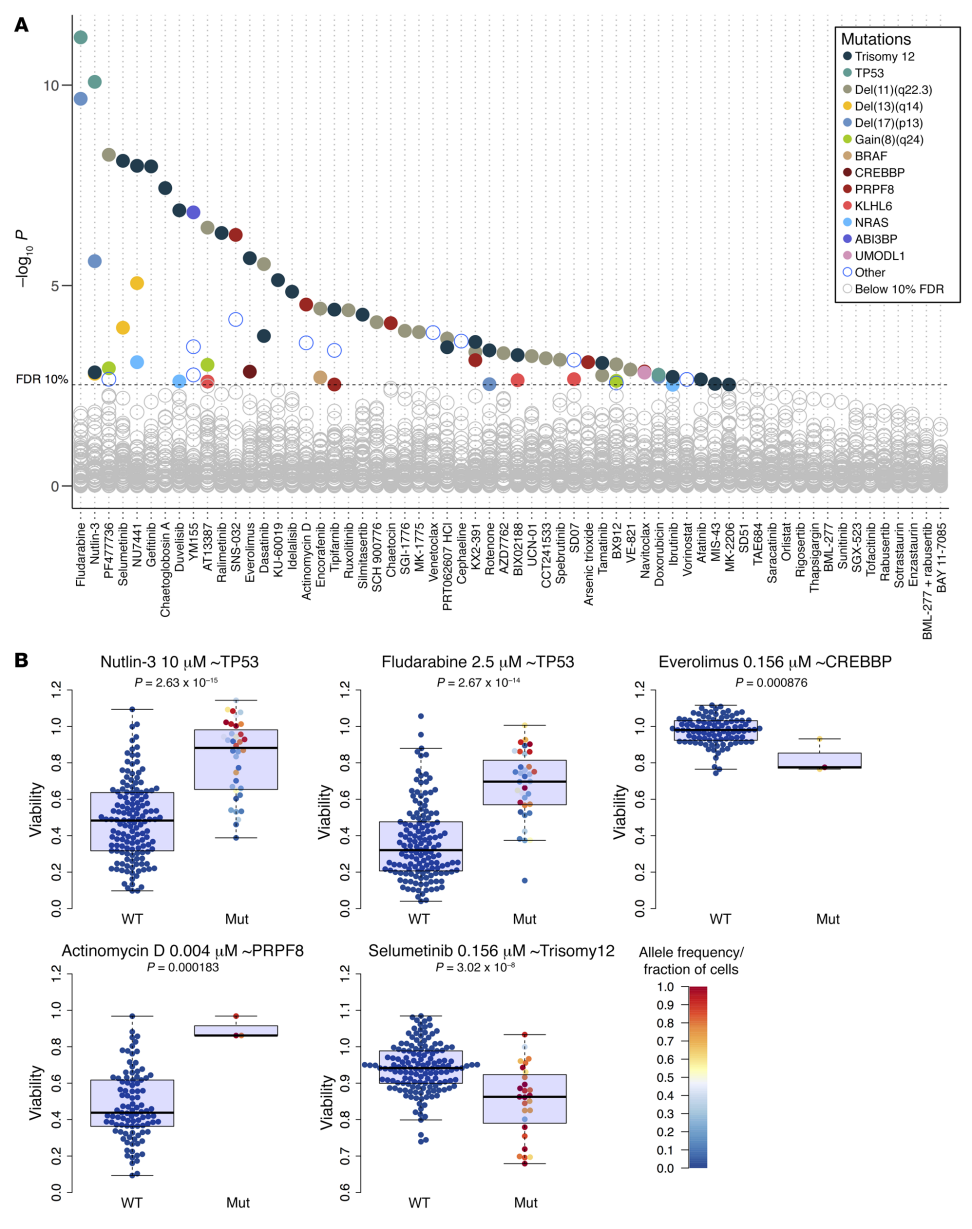
Genomics of drug sensitivity in CLL. (**A**) Drug responses are modulated by many of the mutations recurrent in CLL. The *y* axis shows the negative logarithm of the *t*-test *P* values of all tested associations. Viabilities across different drug concentrations were aggregated using Tukey’s median polish method. Each circle represents a drug-gene association. Tests with *P* values smaller than the threshold corresponding to a false discovery rate (FDR) of 10% (method of Benjamini and Hochberg) are indicated by colored circles, where the colors represent the gene mutations and structural aberrations. To control for potential confounding effects of prior treatment history of the donating patients, we also performed this analysis with pretreatment status as a blocking factor in the association tests; the results of this analysis are provided in Supplemental Figure 19 and are concordant with those shown here (Supplemental Figure 20). (**B**) Primary data of selected drug-gene associations. The fraction of cells for trisomy 12 and the allele frequency (AF) for the mutations *TP53*, *PRPF8*, and *CREBBP* is shown by the color code.

**Figure 10:**
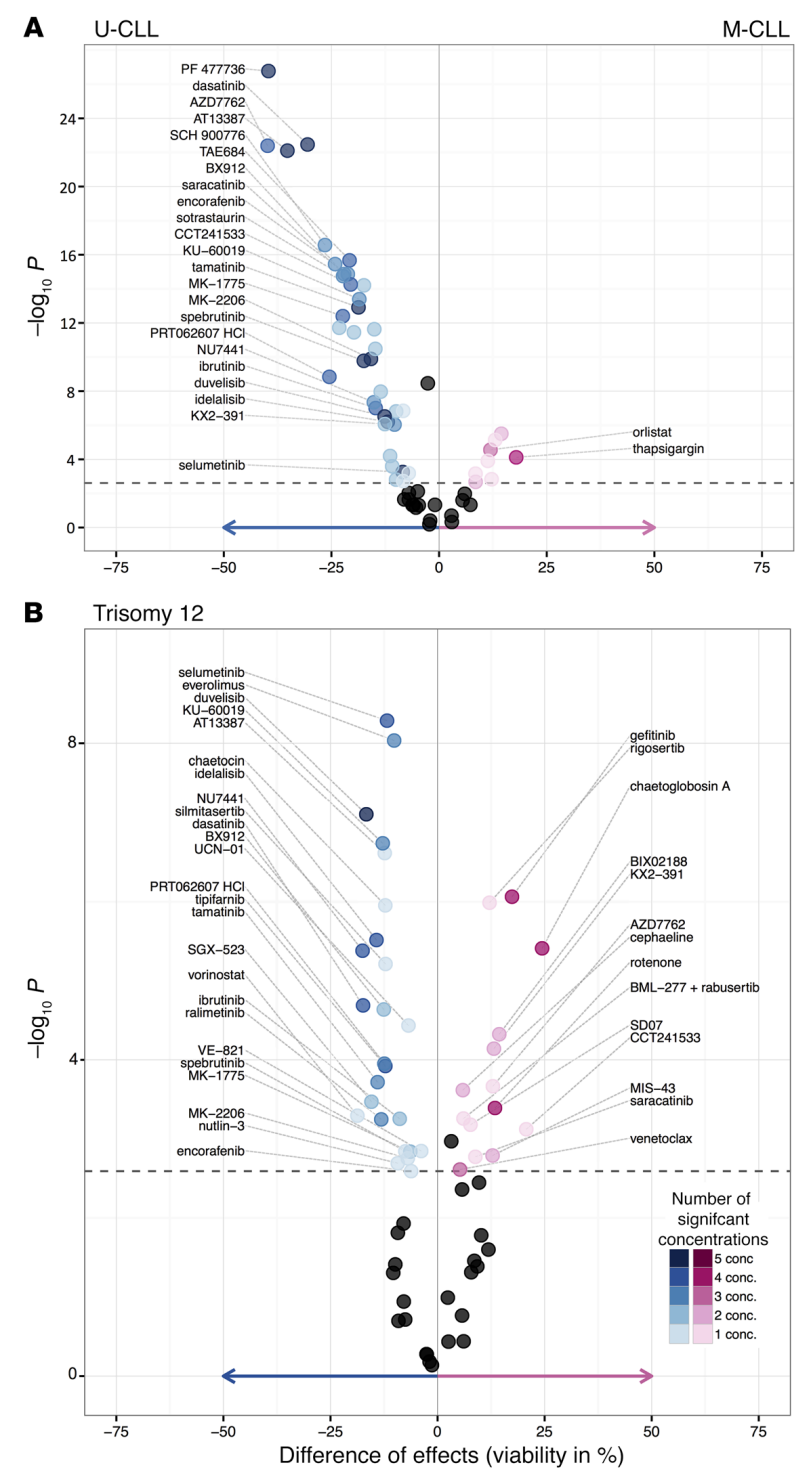
Impact of IGHV and trisomy 12 status on drug sensitivity in CLL. (**A**) Drug responses are modulated by IGHV status. The units on the *x* axis of the volcano plot are the difference in percentage viability; negative values indicate higher sensitivity in U-CLL than in M-CLL. For each drug, the 5 concentration steps were tested separately. Drugs with 3 or more significant associations are labeled, and the largest viability effect and corresponding *P* value are shown. Significant differences were evident for core BCR pathway inhibitors (duvelisib, idelalisib, spebrutinib), nominal CHEK inhibitors (PF477736, AZD7762, SCH 900776), AT13387, and dasatinib. The dashed line indicates an FDR of 10% (*P* < 0.0026). (**B**) Similar to panel **A**, for trisomy 12. Negative values indicate higher sensitivity in cases with trisomy 12.

Most cancer mutations have not been linked to drug response. Based on the cohesiveness of the above results, we used PACE to perform a comprehensive survey of genetic determinants of drug response in CLL, including IGHV status, somatic gene mutations, and structural variants (Figure 9). The most prominent factor was IGHV mutation status, which was associated with response to 42 (67%) drugs (*t* test, FDR = 10%), including idelalisib and ibrutinib, which are in clinical use (Figure 10A). Robust differences were seen even at the lowest concentrations. For instance, 156 nM ibrutinib led to a mean viability of 89.2% in U-CLL versus 99.5% in M-CLL (*P* < 0.001). These effect sizes are comparable to previous, smaller studies investigating individual drug effects (48). We confirmed them in a FACS-based annexin V/propidium iodide assay for apoptosis (Supplemental Figure 16). Similarly, several multi-kinase inhibitors were more active in U-CLL. Indeed, the strongest associations of response with IGHV status were observed for dasatinib and for 3 of the drugs already discussed above, the HSP90 inhibitor AT13387 and the CHEK inhibitors PF477736 and AZD7762. These results show how the critical role of BCR signaling renders CLL cells sensitive to a broad range of kinase inhibitors that act by multiple target engagement of BCR components. While our data show direct correspondences between the individual signaling activity pattern of a tumor and its response to ex vivo drug testing, they also highlight the caveat that clinical translation requires more sophistication than naive indication based on effect size in the assay; trials of dasatinib in CLL had limited success (49).

Responses to 53 drugs (84% of compounds) were modulated by at least 1 mutation (including IGHV), and 39 (62%) of drugs were associated with 2 or more mutations, indicating that the influence of gene mutations on drug responses is more pervasive than anticipated based on cell line–based surveys (3, 5). These mutations targeted diverse molecular processes (Figure 9 and Supplemental Figure 17) including DNA damage (del17p, *TP53*), MEK/ERK signaling (*RAS*, *BRAF*), transcription regulation (*CREBBP*), pre-mRNA processing and splicing (*PRPF8*), but also comprised mutations with less well-understood function (*UMODL1*, gain8q24, *ABI3BP*).

*TP53* mutations, which often co-occur with deletion of one allele of chromosome 17p, are associated with clinical resistance to chemotherapy and are the only genetic marker currently used to guide treatment decisions in CLL (50). Their effect was captured by PACE; fludarabine and doxorubicin had reduced activity in CLL with *TP53* mutation or del17p13 (Figure 9 and Supplemental Figure 17, A and B). Nutlin-3, which targets the MDM2/p53 interaction, also had decreased effect in *TP53*-mutant CLL (Figure 9B). Within mutant cases, the viability effects were associated with clone size, as expected from the drug’s mechanism of action. Analogous associations between *TP53* and response to nutlin-3 and fludarabine were found in MCL (Supplemental Figure 17C).

We investigated the impact of pretreatment status on gene–drug response associations, since 52 of 184 CLL patients had received treatment with chemotherapy and immunotherapy or either alone before sample collection. None of them had received kinase inhibitors. A notable difference between the pretreated and untreated samples was a higher prevalence of *TP53* mutations in the pretreated group (*P* = 4.7 × 10^–7^, Fisher test), a consequence of clonal selection under chemotherapy. Consequently, pretreated samples showed less response to fludarabine (Supplemental Figure 18) and nutlin-3, drugs with strong dependence on p53 function. The second main difference between pretreated and untreated samples was the higher prevalence of U-CLL cases (*P* = 2.3 × 10^–5^, Fisher test), due to progressive disease and thus more frequent need for treatment of U-CLL. This explains the stronger response to kinase inhibitors of pretreated samples. However, when considering pretreatment status separately in the *TP53*–wild type and -mutant groups, or in U-CLL and M-CLL, the association of pretreatment status with response to fludarabine or ibrutinib disappeared. We also systematically analyzed the impact of pretreatment and used pretreatment status as a blocking factor in the association tests of drug responses and genetic features (Supplemental Figure 19). A comparison of the association test *P* values shown in Figure 9 and the same analysis blocked for pretreatment indicates that the 2 analyses are highly concordant (Supplemental Figure 20). These results suggest that effects of pretreatment with chemo-immunotherapy were largely captured by *TP53* and IGHV mutation status, and otherwise were negligible with regard to our drug response association analyses.

HCL cases, which all carried the *BRAF* V600E mutation, had distinctive sensitivity to BRAF and MEK inhibition (Supplemental Figure 17D), whereas in *BRAF*-mutated CLL, the response to BRAF inhibition was less pronounced. This finding suggests that *BRAF* mutations are key disease drivers in HCL, but not CLL, where alternative survival signals (BCR) dominate also in the context of *BRAF* mutations. Indeed, only 3 of 10 *BRAF*-mutant CLL cases had the V600E substitution, and only 2 of these were clonal. *KRAS*-mutant CLL was sensitive to MEK inhibition, and showed increased viability with the BRAF inhibitor encorafenib, reflecting paradoxical BRAF activation (Supplemental Figure 17E).

CLL samples with mutations of the transcriptional cofactor *CREBBP*, known as a key driver in follicular lymphoma (51), were more sensitive to the mTOR inhibitor everolimus. *UMODL1* mutations were associated with resistance to BH3 mimetics (Supplemental Figure 17F).

Trisomy 12 is observed in 15%–20% of CLL and while clinically distinct, little is known about the molecular pathways involved. CLL with trisomy 12 showed a characteristic response profile with multiple drug associations, including increased sensitivity to PI3K, mTOR, and MEK inhibitors (Figure 10B). These associations persisted when we assessed them separately within U- and M-CLL (Supplemental Figure 21). In addition, we observed associations that were present only within M-CLL, including reduced sensitivity to chaetoglobosin A for cases with trisomy 12. To further investigate the relationship between MEK/ERK signaling and trisomy 12, we studied additional ERK (SCH772984) and MEK (cobimetinib, trametinib) inhibitors. These also showed preferential activity in CLL with trisomy 12 (Supplemental Figure 22), pointing to an essential role for MEK/ERK signaling in CLL with trisomy 12.

To explore the effect of trisomy 12 at the level of gene expression, we compared RNA-Seq data of CLL with and without trisomy 12. In addition to the expected gene dosage effect on chromosome 12 (Supplemental Figure 23A), we found 109 differentially expressed genes not on chromosome 12, based on stringent cutoffs (FDR = 0.1 and absolute logarithmic [base 2] fold change > 1.5). Of these, 72 were up- and 37 downregulated (Supplemental Figure 23B). We performed parametric analysis of gene set enrichment (PAGE) (52) on a more permissive list of all genes with a raw *P* value less than 0.05. This analysis linked trisomy 12 to gene sets annotated with BCR, PI3K, AKT, and mTOR signaling, chemokine signaling, and with regulation of the actin cytoskeleton (Supplemental Figure 24).

These results indicate that CLL with trisomy 12 has a specific signaling signature. Indeed, this disease subgroup was reported to exhibit increased p-ERK levels (53), shorter time to progression (54), and a distinct response to the BTK inhibitor ibrutinib (55). To explore the role of trisomy 12 across cancer types, we tabulated its incidence in the Mitelman database (56) and found it strongly overrepresented in tumors with B cell lineage (Supplemental Figure 25). Altogether, these findings suggest that trisomy 12 drives B cell lymphoma by modulating PI3K, MEK/ERK, and mTOR pathways and amplifying BCR signaling.

We conclude that in addition to the known biomarkers in CLL, there are a surprisingly large number of gene-drug associations, which in view of the disease’s genetic heterogeneity implies a commensurate heterogeneity in responses to drugs. The example of trisomy 12 shows how an association of a genetic feature with a spectrum of drugs can elucidate molecular mechanisms. Associations can be context-specific, as exemplified by the driver versus passenger nature of *BRAF* mutations in HCL versus CLL.

### Understanding complex networks of drug response predictors.

**Figure 11:**
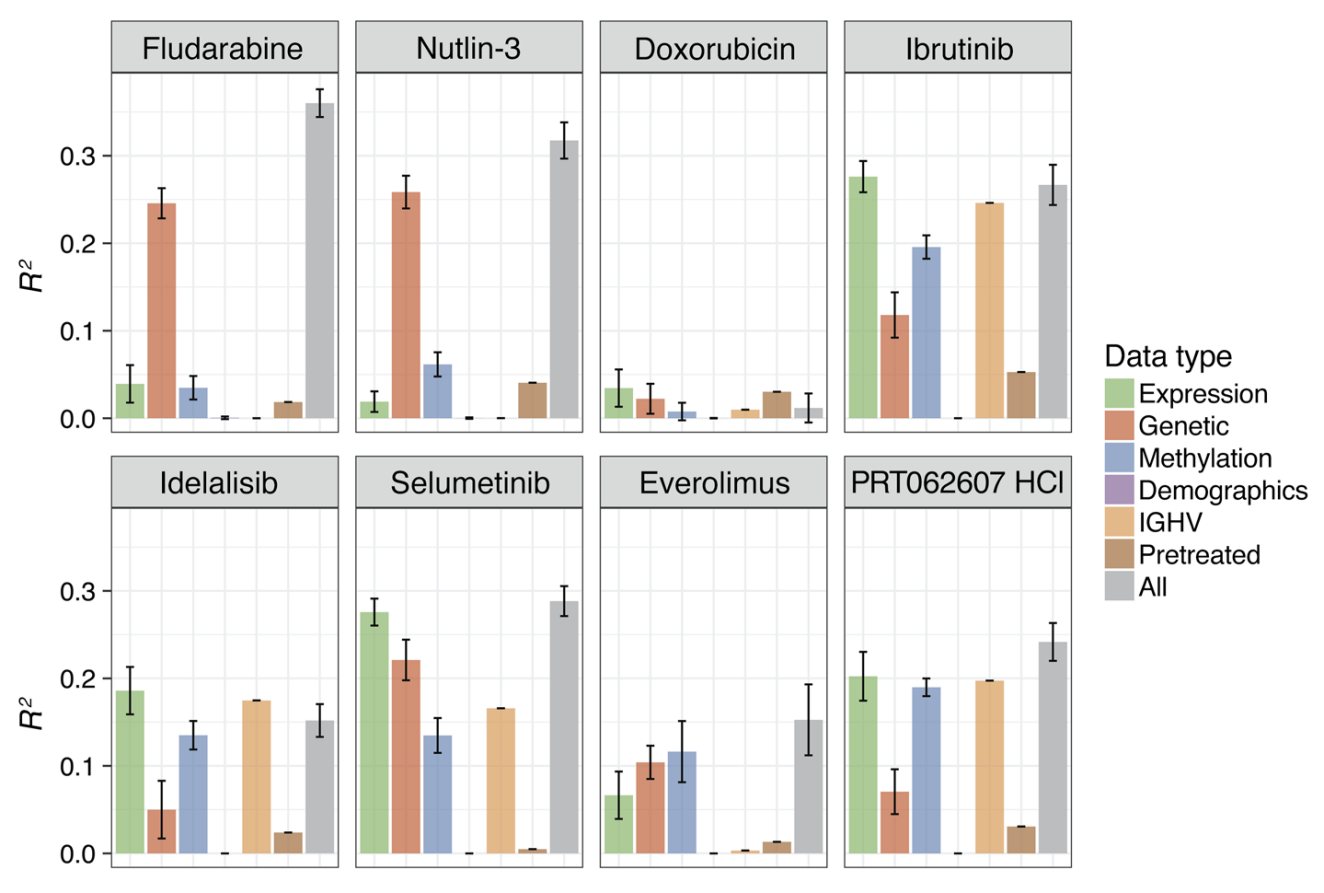
Explanatory power of data types for drug response prediction. Explanatory power (R^2^) of the features from the different data types for prediction of drug response. For fludarabine, doxorubicin, and nutlin-3, we fit multivariate regression models to predict the average viability value across all 5 concentrations. For the targeted drugs ibrutinib (BTK), idelalisib (PI3K), selumetinib (MEK), everolimus (mTOR), and PRT062607 (SYK), we used the average of the 2 lowest concentrations, 156 and 625 nM, as the dependent variable. As predictors, we used the different data types as indicated by the colors: demographics (age, sex), mutations, IGHV status, pretreatment (coded as 0/1), and the top 20 principal components of the gene expression or DNA methylation data matrices. In addition to using each data type separately, we also fit models with all data types combined (gray). L1 (lasso) regularization was used, with the parameter lambda chosen by cross-validation, and shown are mean and standard deviation across 100 repetitions. Drug responses to nutlin-3 and fludarabine were predominantly explained by gene mutations and copy number variants (genetics). In contrast, response to kinase inhibitors was best explained by IGHV status, gene expression, or methylation patterns.

**Figure 12:**
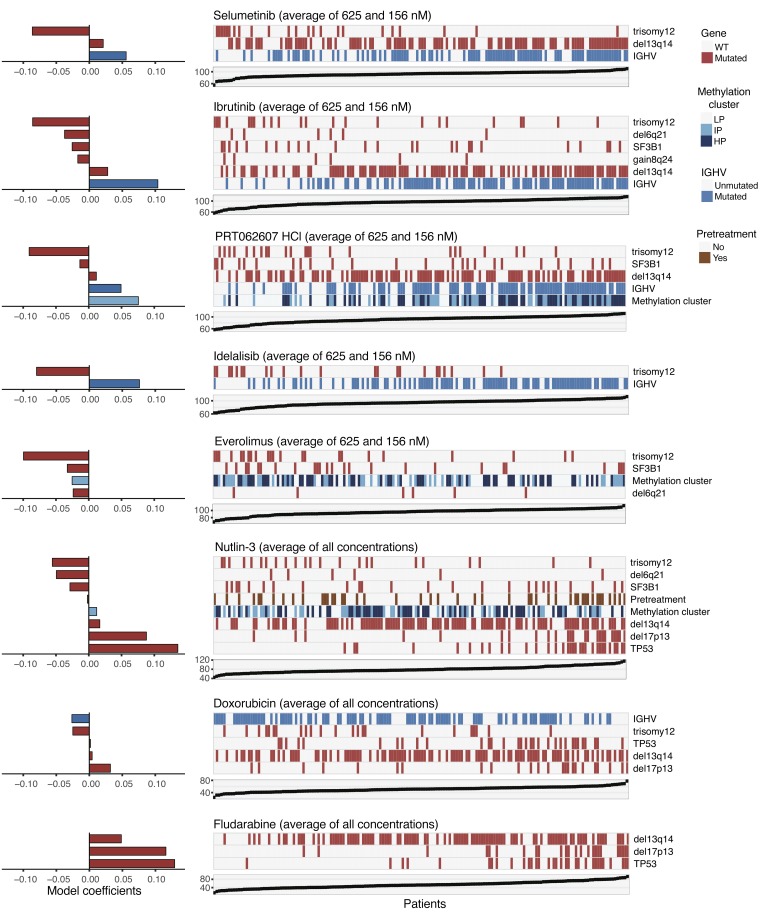
Multivariate regression models for drug response. Visualization of fitted adaptive L1 (lasso) regularization multivariate models using gene mutations, IGHV status, pretreatment, and methylation clusters (coded as 0/0.5/1) as predictors (gene expression and DNA methylation principal components were set aside due to redundancy). Each matrix shows the predictor values corresponding to the model for a drug, and the response values are shown in the scatter plot below. The fitted model coefficients are shown by horizontal bars. Negative coefficients (e.g., trisomy 12) indicate lower viability after drug treatment (i.e., greater sensitivity) if the feature is present. The red and blue boxes indicate the non-zero regression coefficients and their signs LP, low programmed; IP, intermediate programmed; HP, high programmed.

The molecular basis of variable drug response phenotypes is multifactorial and can involve multiple layers including gene mutations, gene expression, and DNA methylation (7). While the results presented above provide a comprehensive catalog of marginal associations of single mutations, an understanding of the combinatorial interplay of multiple factors will be essential for a meaningful prediction of drug response. To address this challenge, we applied linear regression with lasso regularization and derived for each drug a multivariate predictor composed of genetic, gene expression, and DNA methylation covariates (Figures 11 and 12) (57). We first assessed to what extent single omics data types or the combination of all our omics data explained the variable drug responses. Responses to chemotherapeutics and nutlin-3 were predominantly explained by genetic factors, whereas response to BCR inhibitors was best predicted by IGHV, gene expression, and DNA methylation (Figure 11).

Next, we visualized predictor profiles for individual drugs, focusing on the genetic variables and a 3-category summary of the DNA methylation data (58). The profiles were reflective of the drugs’ mechanisms of action. For nutlin-3 and fludarabine, *TP53* and del17p were the most dominant predictors (Figure 12). The predictor profiles for BCR inhibitors highlighted IGHV status and trisomy 12 as key factors, but additional aberrations including del13q14 contributed to the predictors for ibrutinib and the SYK inhibitor PRT062607 (Figure 12). Pretreatment status was not selected as a prediction feature for most drugs, and where selected its coefficient was mostly small, but there were a few notable exceptions, including rotenone (Supplemental Figure 26).

Our multivariate analysis points to a fundamental and previously underappreciated role of trisomy 12 in CLL biology. It also highlights roles of other aberrations, including del13q14, and suggests that DNA methylation patterns contain information about disease biology beyond what is implied by IGHV status.

### Impact on outcome.

**Figure 13:**
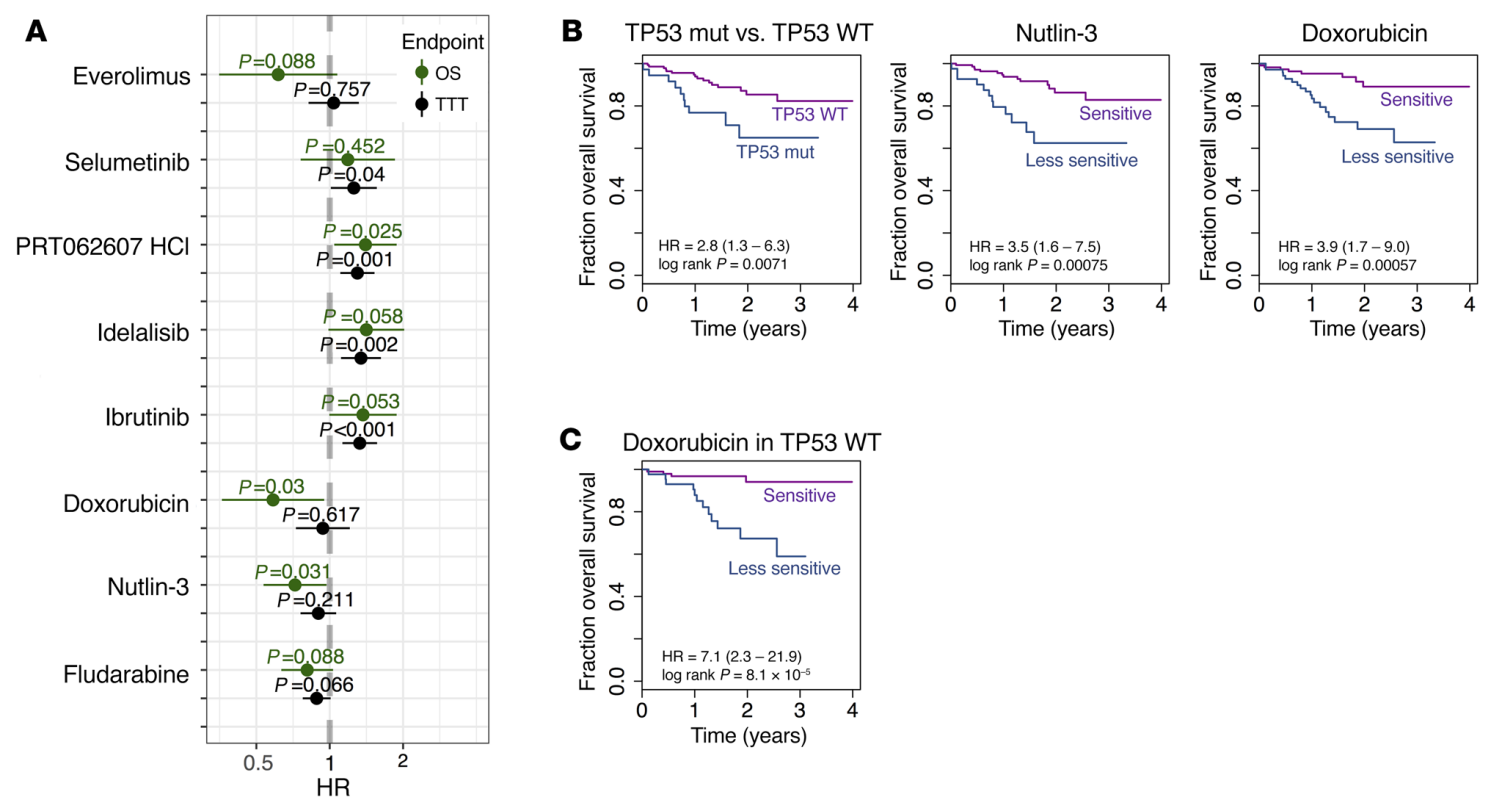
Ex vivo drug response and outcome. (**A**) Association of drug responses with time from sampling to treatment (TTT; *n* = 174) and overall survival (OS; *n* = 184), assessed by univariate Cox regressions. Shown are estimated hazard ratios (HR) and 95% confidence intervals. The average viability values, across all 5 concentrations for fludarabine, doxorubicin, and nutlin-3, and across the 2 lowest concentrations 156 and 625 nM for the targeted drugs ibrutinib (BTK), idelalisib (PI3K), selumetinib (MEK), everolimus (mTOR), and PRT062607 (SYK), were scaled such that a unit change of the regressor corresponds to 10% change in cell viability. (**B**) Kaplan-Meier plots for OS stratified by *TP53* mutation status, and nutlin-3 and doxorubicin response. Patient groups of nutlin-3 or doxorubicin responders and weak responders were defined by ex vivo drug responses dichotomized using maximally selected rank statistics to visualize effects. The same 172 CLL patient samples were used for all 3 Kaplan-Meier plots. Thirty-six patient samples were *TP53* mutated, and 39 and 40 patient samples were in the nutlin-3 or doxorubicin weak-responder groups, respectively. (**C**) Analogous to the rightmost plot in panel **B**, but limited to patients with wild-type *TP53*.

The use of primary patient cells allowed us to assess associations of drug response phenotypes with clinical outcomes. Consistent with previous reports (24, 50), mutations of *TP53*, *SF3B1*, and *BRAF*, the deletions del17p13 and del11q23, and IGHV status were individually associated with TTT and/or overall survival (OS) (Supplemental Figure 27). We asked whether ex vivo drug responses predicted outcome and to what extent they could improve upon the established biomarkers. First, we considered drugs whose response was associated with *TP53* mutation status, the most prominent clinical biomarker for CLL. We found that good responses to doxorubicin, fludarabine, and nutlin-3 were each predictive of better OS (Figure 13, A and B); when limiting the analysis to untreated patients, the results were similar (Supplemental Figure 28). Moreover, these results were only partially explained by *TP53* mutation status, since within wild-type *TP53* CLL, doxorubicin response had predictive value for OS (Figure 13C). Next, we fitted multivariate Cox models (Supplemental Tables 6–10) using established covariates [age, pretreatment, trisomy 12, del11q22.3, del17p13, *TP53* mutation, IGHV status] and individual drug responses as continuous variables. Again, doxorubicin response was associated with OS (*P* = 0.03, Supplemental Table 6). Response to BCR inhibitors was associated with inferior TTT (ibrutinib, idelalisib, PRT062607) and OS (PRT062607) (Figure 13A) and was partly explained by association with IGHV status. However, also within M-CLL, response to BCR inhibition was negatively correlated with TTT, and BCR inhibitors were significantly associated with TTT in multivariate models considering age, pretreatment, trisomy 12, del11q22.3, del17p13, *TP53* mutation, and IGHV status (Supplemental Tables 8–10). Together, these results show that drug-response phenotyping reads out disease-relevant biology beyond what is conferred by established biomarkers.

## Discussion

Our work maps the drug sensitivity landscape of primary leukemia and lymphoma cells and links response phenotypes to underlying molecular properties. We demonstrate that biomarkers for drug response can be read out by short-term drug response profiling within days, and that their information content matches or exceeds conventional biomarkers as well as omic profiling. PACE recapitulates the complete spectrum of known biomarkers (e.g., *TP53*, *BRAF*, *RAS*, and IGHV mutations) and reports a surprisingly large set of previously unappreciated modifiers of response to drugs including chemotherapeutics and targeted agents.

Within CLL, we developed a functional disease classification based on BCR, MEK, and mTOR signaling and demonstrate that the resulting groups are characterized by distinctive sensitivities to many drugs. Although our current classification is a simplification and is likely to evolve, we show that it uncovers disease-relevant biology and bears the potential for clinical exploitation. The model identifies CLL cases that predominantly rely on BCR signaling, and cases in which BCR-independent alternative signaling activities contribute to cell survival and proliferation. These functional disease groups showed clear enrichments with regard to many genetic features, but our attempts at describing the drug response groups through classification approaches using genetic features failed, which further highlights the unique and nonredundant information conveyed by functional readouts for precision oncology.

The distinct drug response phenotype of trisomy 12 in CLL implies amplification of BCR signaling as the mechanism underlying this driver mutation, a finding that would explain short progression-free survival (54), high p-ERK levels (53), the different response pattern to ibrutinib (55), and the characteristic incidence of trisomy 12 in B cell malignancies.

Even though the assay does not explicitly probe any particular drug’s mechanism of action, our results show that cell viability profiles measured across a diverse spectrum of drugs and many samples constitute unique footprints that can be used, via similarity and clustering, to sort tumors and drugs into biologically meaningful groups. Moreover, such profiles can be used to reveal individual tumor’s pathway dependencies, and to discover drug repurposing opportunities. While precise molecular understanding of factors underlying response remains a fundamental goal, clinical exploitation may start from such phenotypic readouts. In this respect, one clinically exploitable finding is the cosensitivity patterns observed for the drug sensitivity groups, which can provide a starting point for the development of combination therapies.

Our study extends the range of available biomarker types for blood cancers, which currently include IGHV status, DNA methylation profile, gene expression, and gene mutations (59). We were able to predict clinical endpoints in CLL from ex vivo drug response data. In part, this reflected transitive associations with established genetic markers, but multivariate analysis showed that drug response phenotypes improve current models. Hence, ex vivo drug response testing presents a powerful window into cells that is often more directly linked to cell physiology than current molecular data.

Prior efforts in biomarker development employed large-scale cell line–based drug screens (3–5, 60) and have sparked successful efforts of reanalysis (61). On the other hand, there were challenges to reproducibility across laboratories (62), and experiences with the older generation of chemosensitivity tests have been disappointing (63). Current improvements to molecular characterization and data analysis suggest consilience at the level of detected biological associations (64). Here, we show high consistency between drug-genotype associations measured in a primary-cell-based assay with molecular and clinical data. Crucial for the cogency is the large number of patients sampled, which provides statistical power and reduces spurious associations. In contrast to cell line–based screens that yielded surprisingly few truly novel genotype-phenotype associations (3–5), our data show that high sensitivity to discover molecular associations can be achieved by studying primary tumor samples in sufficient numbers within disease entities. In this manner, the potentially strong effects of cell of origin can be disentangled.

In our analysis, we considered cell viabilities at one or a few well-chosen drug treatment doses and thus avoided fitting parametric dose-response curve models or otherwise summarizing dose-response data across a wide range of doses. This choice was motivated by dose-dependent polypharmacology. In particular, kinase inhibitors typically bind to multiple kinases, with different affinities for each, and with different biological effects on cell physiology of each binding event. For instance, for encorafenib our data were consistent with relatively specific binding to *BRAF* V600E at low drug concentrations, whereas for higher concentrations other kinases appeared affected, too. Summarizing such data into a single value would, in effect, obscure the mutation-specific effect of this drug. More generally, at higher concentrations generic toxicity is expected to dominate over specific target effects. Moreover, to the extent that the data are intended as a model for what may happen in vivo, interest is on the effect of a drug at a concentration it will have at a therapeutic dose, not at exponentially higher or lower concentrations.

Our work highlights the complexity of genotype-phenotype relationships in cancer, which cannot be captured by simple univariate associations. Multivariate modeling indicated variable explanatory power of different omics data types, with drug-dependent model complexity and prediction performance. For instance, the response to BCR inhibitors depended on IGHV status (including its associated gene expression and DNA methylation patterns) and trisomy 12, reflecting the multiple layers of biology involved. PACE (http://pace.embl.de) provides a data resource to study such relationships in depth.

Short-term ex vivo drug assays coanalyzed with molecular profiles have the potential to become a key instrument to uncover mechanisms underlying drug response variation and to develop precision cancer care and stratification.

## Methods

### Patient samples.

We included peripheral blood samples from 246 leukemia and lymphoma patients and 3 healthy donors (Supplemental Table 3). Blood was separated by a Ficoll gradient (GE Healthcare), and mononuclear cells were cryopreserved.

### Compounds.

Compounds were obtained from Sigma-Aldrich, Enzo Life Sciences, Selleck Chemicals, and Merck and were dissolved in DMSO at 0.1–50 mM (mainly 10 mM) and stored at –20°C. For a detailed list of compounds, see Supplemental Tables 1 and 2. Spebrutinib was obtained from Celgene, LGX818 from Novartis. ROS-targeting agents (MIS-43, SD07, SD51) were provided by A. Mokhir, Erlangen, Germany.

### Drug response assays.

Drug response assays were performed with RPMI-1640 (Invitrogen) supplemented with penicillin/streptomycin (Invitrogen), L-glutamine (Invitrogen), and 10% pooled and heat-inactivated AB-type human serum (RPMI-HS, MP Biomedicals). Final DMSO concentrations did not exceed 0.5%. Cell viability was determined after 48 hours using the ATP-based CellTiter Glo assay (Promega). Luminescence was measured with a Tecan Infinite F200 Microplate Reader (Tecan Group AG) and with an integration time of 0.2 seconds per well. We verified the linearity of the relationship between the readout of the CellTiter Glo assay and cell count through a dilution series (1 × 10^6^ to 1 × 10^3^ cells per well), which we performed in 384-well format. CellTiter Glo reagent for stable luminescence was titrated, and a volume of 12 μl/well was picked for all reported experiments. We performed a pilot screen in 384-well format with 67 compounds (for 16 drugs with one and 51 drugs with two concentrations), using duplicate wells per drug and concentration. We plated compounds in polypropylene 96-well storage plates (Thermo Fisher Scientific), which were stored at –20°C. For each batch of samples, a new drug-storage plate was thawed and compounds were diluted by addition of RPMI-HS. Ten microliters of the compound dilutions were plated in white 384-well assay plates (Greiner Bio One). Plates were sealed with breathable foil (Sigma-Aldrich) in order to reduce evaporation on plate edges. Cells were incubated with compounds for 48 and 72 hours at 37°C in a pilot experiment, followed by immediate readout. As few effects were exclusively observed at 72 hours, we performed the main screen using a 48-hour incubation time only to reduce potential noise. We used 1 well per drug and concentration in the plate design. Sixty-four drugs in 5 concentrations across 249 patient samples were studied. Due to instability and subsequent batch effects, bortezomib was excluded. For all downstream analysis 63 drugs were used. Screening was done in 384-well assay plates. Drugs were preplated and frozen. For screening, we selected patient donors who had a white blood cell (WBC) count greater than 25,000 and samples for which we had at least 5 × 10^7^ cells available. After thawing and DMSO removal, primary patient cells were incubated in cell culture medium at room temperature for 3 hours on a roll mixer. The intention of this procedure was 2-fold: (a) to completely wash out remaining DMSO, and (b) to only consider cells during cell counting that survived the freezing procedure. Although the percentage of cells that survived the freezing was variable between patient samples, we observed no significant loss of cell viability during the 48-hour incubation time (in negative control wells, i.e., without drug treatment), as shown in Supplemental Figure 29. Indeed, we observed a trend for increased ATP luminescence after the 48-hour incubation, perhaps due to recovery of the cells from freezing stress. Of note, no cell proliferation is expected in these culture conditions. For each sample, we dispensed a volume of 15 μl in each well of the 384-well plates with stock solution concentration of 1.3 × 10^6^ cells/ml. The final cell concentration was 2 × 10^4^ cells per well.

### Genome and transcriptome analysis.

For 107 patients, we performed WES on tumor DNA and constitutive normal DNA. DNA was extracted using the QIAamp DNA kit (Qiagen) according to manufacturer’s protocol. DNA quantification was performed on a Qubit 2.0 Flourometer (Life Technologies). Libraries for WES were prepared on the SureSelect Automated Library Prep and Capture System (Agilent Technologies) according to the manufacturer’s protocol (version E.3). In brief, genomic DNA (1.5–3 μg) from each sample was fragmented to a length distribution peak of 150 to 200 nt for the preparation of paired-end sequencing libraries. Enrichment for exomic sequence was performed using Agilent SureSelect V4+UTR in-solution capture reagents following vendor’s protocol v2.0.1. Sequencing was carried out on HiSeq 2000 machines (Illumina) with 3 samples multiplexed per lane.

For RNA-Seq, RNA was extracted from 123 patients using the RNA RNeasy mini kit (Qiagen) according to the manufacturer’s protocol. RNA quantification was performed on a Qubit 2.0 Flourometer. Quality was assessed on an Agilent 2100 Bioanalyzer. An RNA integrity number (RIN) of at least 8 was required. RNA-Seq libraries were prepared according to the manufacturer’s protocol (Illumina TruSeq RNA sample preparation v2). Sequencing was performed on Illumina HiSeq 2000 machines with 2–3 samples multiplexed per lane.

### Targeted sequencing.

Sequencing was performed on a GS Junior benchtop sequencer (Roche) as described previously (65). Targeted sequencing was performed for *BRAF* (*n* = 231), *NOTCH1* (*n* = 231), *TP53* (*n* = 230), *SF3B1* (*n* = 231), *MYD88* (*n* = 230), *KRAS* (*n* = 188), *NRAS* (*n* = 188), *EZH2* (*n* = 188), and *PIK3CA* (*n* = 188). IGHV analysis was performed as described previously (66).

### DNA copy number variants.

DNA copy numbers were assessed using Illumina CytoSNP-12 and HumanOmni2.5-8 microarrays (*n* = 169). DNA (200 ng) was processed according to the manufacturer’s instructions. Arrays were read out using the iScan array scanner. Copy number variants were verified by using the exome sequencing data (*n* = 107). Fluorescence in situ hybridization (FISH) analysis was performed for del11q22.3 (*n* = 162), del17p13 (*n* = 159), del13q14 (*n* = 155), trisomy 12 (*n* = 152), del6q21 (*n* = 132), and gain8q24 (*n* = 125). Information on structural variants from FISH, exome sequencing, and SNP arrays was combined into 1 table (*n* = 219).

### DNA methylation arrays.

Genome-wide DNA methylation profiling was performed as described previously (58). A total of 196 CLL patients were assayed by Illumina Infinium HumanMethylation 450k or 850k.

### Data availability.

European Genome-Phenome Archive (EGA) accession EGAS0000100174. The complete data and computational analysis code used in this study are available from www.bioconductor.org in the R package pace.

### Statistics.

To quantify the response of a patient sample to a drug at a given concentration, we used viability relative to the control, i.e., the CellTiter Glo luminescence readout of the respective well divided by the median of luminescence readouts of the 32 DMSO control wells on the same plate. Integrative data analysis of gene and RNA sequencing, CNV, methylation profiles and drug responses was performed using R version 3 and included univariate association tests, multivariate regression with and without lasso penalization, Cox regression, generalized linear models, principal component analysis and clustering. The complete data analysis is described in further detail in the supplemental methods (Section 4), and a computer-executable transcript of analyses is provided in the form of Rmarkdown files via http://pace.embl.de

### Study approval.

The study was approved by the Ethics Committee Heidelberg (University of Heidelberg, Germany; S-206/2011; S-356/2013). Patients who donated tumor material provided written informed consent prior to study.

## Author contributions

WH and TZ conceptualized the study. LS, MSL, and TZ designed the experiments. SD, MO, SA, J Lu, VK, BV, Andreas Mock, Martin Sill, MZ, WH, and TZ performed software-based and formal analyses and data visualization. L Wagner, M Słabicki, S Wang, A Mokhir, S Oppermann, J Lewis, LS, SD, JH, BW, ML, LAS, EY, TW, SR, AB, CCO, A Jethwa, SGD, MB, TO, XL, KSL, A Jauch, RR, KP, and TZ performed methodology and experimental investigations. SD, MO, SA, VK, BV, TZ, and WH wrote the original draft of the manuscript. All authors reviewed and edited the manuscript. LS, SD, BW, TW, AKO, MO, JL, WH, and TZ were responsible for data curation. CK, CL, DR, AM, KZ, MH, FNK, CP, EA, SM, ADH, MH, JD, IR, and TZ provided resources.

## Supplementary Material

Supplemental data

## Version 1 12/11/2017

Electronic publication

## Version 2 01/02/2018

Print issue publication
