# Supplementary material for: Drug-perturbation-based stratification of blood cancer
Source: J Clin Invest. 2017 Dec 11;128(1):427–45. doi: 10.1172/JCI93801 (PMC5749541; doi:10.1172/JCI93801)
Supplement: Supplemental data [file jci-128-93801-s001.pdf]

Supplementary Figures and Tables

November 30, 2017

Drug Perturbation Based Stratification of Blood Cancer

Sascha Dietrich, MD*, Małgorzata Oleś, PhD*, Junyan Lu, PhD*, Leopold Sellner, MD, Simon Anders, PhD, Britta Velten, Bian Wu, MD, Jennifer Hüllein, PhD, Michelle da Silva Liberio, Tatjana Walther, Lena Wagner, Sophie Rabe, Sonja Ghidelli-Disse, Marcus Bantscheff, PhD, Andrzej K. Oleś, PhD, Mikołaj Słabicki, PhD, Andreas Mock, Christopher C. Oakes, PhD, Shihui Wang, Sina Oppermann, Marina Lukas, Vladislav Kim, Martin Sill, PhD, Axel Benner, Anna Jauch, PhD, Lesley Ann Sutton, PhD, Emma Young, Richard Rosenquist, PhD, Xiyang Liu, MD, Alexander Jethwa, Kwang Seok Lee, Joe Lewis, PhD, Kerstin Putzker, Christoph Lutz, MD, Davide Rossi, MD, Andriy Mokhir, PhD, Thomas Oellerich, MD, Katja Zirlik, MD, Marco Herling, MD, Florence Nguyen-Khac, MD, Christoph Plass, PhD, Emma Andersson, Satu Mustjoki, PhD, Christof von Kalle, MD, Anthony D. Ho, MD, Manfred Hensel, MD, Jan Dürig, MD, Ingo Ringshausen, MD, Marc Zapatka, PhD, Wolfgang Huber, PhD and Thorsten Zenz, MD.

* These authors contributed equally to this work

Contents

1 Data availability 5

2 Supplementary methods 5
2.1 Western blot analysis 5

3 Quality assessment and control 5

4 Data analysis 5
4.1 Raw data analysis 5
4.2 Whole exome sequencing analysis 6
4.3 RNA-seq analysis 6
4.4 SNP array analysis 6
4.5 Integrative data analysis 6
4.6 Associations of ex-vivo drug responses with genomic features 6
4.7 Batch effects 6
4.8 Gene expression and gene set enrichment analysis 7
4.9 Survival analyses 7
4.10 Penalized multivariate regression 7

5 Supplementary figures 8

6 Supplementary tables 37

List of Figures

S1 Drug induced effects on cell viability . . . 8
S2 Inhibition of calcium release after IgM stimulation . . . 8
S3 Effect of AZD7762 on key signaling cascade members of the B-cell receptor pathway . . . 9
S4 AZD7762 induces apoptosis of primary CLL cells . . . 10
S5 Association between the response to HSP90 inhibitors and IGHV status . . . 11
S6 Drug-drug correlations in MCL and T-PLL . . . 12
S7 Summary of disease-specific drug effects . . . 13
S8 Global overview of drug response landscape . . . 14
S9 Relative effects of BCR inhibitors (SYK, PI3K), everolimus (mTOR) and selumetinib (MEK) on cell viability and gene expression . . . 15
S10 Derivation of thresholds for the decision tree model . . . 16
S11 Characterization of drug response groups in CLL . . . 17
S12 Gene expression associated with the BTK group . . . 18
S13 Gene expression associated with the MEK and mTOR groups . . . 19
S14 Correlation of IL-10 mRNA expression and response to everolimus within the mTOR group . . . 20
S15 Response to cytokines in CLL . . . 21
S16 Ibrutinib induces apoptosis in primary CLL cells . . . 22
S17 Effect of mutations on drug response . . . 23
S18 Drug response in CLL stratified by pretreatment . . . 24
S19 Associations of drug sensitivities with gene mutations and structural aberrations in CLL . . . 25
S20 Impact of pretreatment on associations of drug sensitivities with gene mutations . . . 26
S21 Drug response associated with trisomy 12 within U-CLL and M-CLL . . . 27
S22 Drug response associated with trisomy 12 . . . 28
S23 Gene expression associated with trisomy 12 . . . 29
S24 Gene set enrichment analysis for trisomy 12 . . . 30
S25 Prevalence of trisomy 12 across different malignancies . . . 31
S26 Lasso model for rotenone . . . 31
S27 Impact of genetic factors on survival . . . 32
S28 Impact of drug response on survival in untreated patients . . . 33
S29 ATP luminescence of DMSO controls at the beginning and after 48 h of incubation . . . 34
S30 Assessment of batch effects . . . 35
S31 Reproducibility of drug response measurements . . . 36

List of Tables

S1 Drugs . . . 37
S2 Drug concentrations . . . 39
S3 Patient characteristics . . . 41
S4 Sequencing quality . . . 45
S5 Target profiling of AZD7762 and PF477736 in cell lysates of K562 cells . . . 48
S6 Multivariate Cox regression model for overall survival with response to fludarabine as a covariate 50
S7 Multivariate Cox regression model for overall survival with response to doxorubicin as a covariate 50
S8 Multivariate Cox regression model for time to treatment with response to ibrutinib as a covariate 51
S9 Multivariate Cox regression model for time to treatment with response to idelalisib as a covariate 51
S10 Multivariate Cox regression model for time to treatment with response to PRT62607 as a covariate 51

1 Data availability

European Genome-phenome Archive (EGA) accession EGAS00001001746.

2 Supplementary methods

2.1 Western blot analysis

Protein amount was determined using the BSA assay following the manufacturer's protocol. For cell lines 30 μg protein and for primary cells 15 μg protein was loaded on a 4–15% gradient SDS polyacrylamide gel (Bio-Rad). For comparative evaluation of molecular mass, 5 μl of PageRuler Prestained Protein Ladder (Thermo Scientific) was loaded next to protein samples. Proteins were transferred to a PVDF membrane (TransBlot® Turbo^{TM} mini-size LF PVDF membrane, Bio-Rad) using the 30 min. standard transfer program of the TransBlot® Turbo^{TM}(Bio-Rad). After blocking with 5% BSA (w/v), membranes were probed with primary antibodies either against PARP or against BTK, Syk, Akt and S6 ribosomal protein (1:1000 dilution, respectively), and against the phospho-proteins p-BTK (Tyr223), p-Syk (Tyr525/526), p-Akt (Ser473) and p-S6 ribosomal protein (Ser240/244) (1:500 dilutions) at 4 degrees celsius overnight, followed by 1 hour incubation at room temperature (RT). Membranes were washed three times with TBST and exposed to the appropriate HRP-conjugated secondary anti-rabbit or anti-mouse antibody (1:10 000) for 1 hour at RT and washed three times with TBST for 10 minutes before chemiluminescence detection. Proteins were detected with an enhanced chemiluminescence substrate solution (EZ-ECL, BI Biological Industries) using the semi-automated Chemidoc camera-based imager (Millipore). Equal protein loading and quality was controlled by stripping the blots for 15-20 minutes at RT in Restore Western blot Stripping buffer (Pierce), washing twice in TBST at RT, blocking with 5% bovine serum albumin for 1 hour, and re-probing the membrane with an anti-Actin antibody (1:10 000) and anti-mouse secondary antibody.

The following antibodies were used: primary antibodies against PARP (Rabbit mAb, #9542), Akt (40D4 Mouse mAb, #2920S), p-Akt (Ser473, D9E Rabbit mAb, #4060S), S6 ribosomal protein (5G10 Rabbit mAb, #2217S), p-S6 ribosomal protein (Ser240/244, Rabbit, #2215S), BTK (D3H5 Rabbit mAb, 8547S), p-BTK (Tyr223, Rabbit, 5082S), p-Syk (Tyr525/526, C87C1 Rabbit mAb, #2710S) were purchased from Cell Signaling Technology (ZA Leiden, Netherlands) and used in a 1:1000 or 1:500 (phospho-antibodies) dilution in 5% BSA. The monoclonal antibody against Syk (4D10 Mouse, #sc-1240) was used at a 1:1000 dilution and was obtained from Santa Cruz Biotechnology. To ensure equal loading membranes were re-probed using the primary anti-ß-actin antibody (8H10D10 Mouse mAb, #3700) from Cell Signaling (ZA Leiden, Netherlands) at 1:5000 dilution or anti-GAPDH-peroxidase antibody (clone GAPDH-71.1 Mouse mAb, #G9295) from Sigma-Aldrich at 1:30000. Mouse and rabbit- secondary HRP-conjugated antibodies were purchased from abcam (rabbit-HRP ab6721, mouse-HRP ab6728) and used at a 1:10000 dilution in 5% BSA.

3 Quality assessment and control

We used multiple steps of data quality assessment. First, we assessed the sensitivity of the platform by asking whether it could detect known gene-drug associations and expected correlations between similar drugs. To assess the robustness of the platform and safeguard against "batch effects" that have the potential to confound high-throughput experiments, we repeated the analyses taking the time point of experiments as potential batch confounders into account Supplementary Fig. S30. In addition, we assessed the reproducibility between different batch time points and compared the same samples from three patients in independent experiments Supplementary Fig. S31. The data were highly reproducible (Pearson correlation coefficients: 0.74, 0.86, 0.92 (48 h) and 0.81, 0.85 and 0.90 (72 h)

4 Data analysis

4.1 Raw data analysis

To quantify the response of a patient sample to a drug at a given concentration, we used viability relative to control; this value is the CellTiter Glo luminescence readout of the respective well divided by the median of luminescence readouts of the 32 DMSO control wells on the same plate. We used the R/Bioconductor package cellHTS2 version 2.26.0 [1] for processing the raw files obtained from the plate scanner.

4.2 Whole exome sequencing analysis

Reads were mapped to the human reference genome (GRCh 37.1 / hg 19) using BWA (version 0.6.2)(68) with default parameters and maximum insert size 1000 nt. BAM files were sorted with SAMtools (version 0.1.19), and duplicates were marked with Picard tools (version 1.90). The resulting mean coverage was 105x (range 56x – 193x) Table S4. For the detection of single-nucleotide variants (SNVs), we applied a genome sequencing analysis pipeline established at DKFZ, which is based on SAM-tools mpileup and bcftools with parameter adjustments to allow for calling of somatic variants with heuristic filtering as previously described [2]. After annotation with RefSeq (version September 2013) using ANNOVAR [3], somatic, non-silent coding variants of high confidence were selected.

4.3 RNA-seq analysis

The RNA-Seq reads were demultiplexed and aligned to the human reference genome (GRCh 37.1 / hg 19) using STAR version 2.3.0 [4] with default parameters. Read counts per gene were obtained with htseq-count [5] using the default mode union. Differential expression calling was performed using DESeq2 [6].

4.4 SNP array analysis

IDAT files were processed using GenomeStudio (Illumina, San Diego, CA, USA). Features located on sex chromosomes were excluded from the analysis. Segmentation of the normalized log2 ratios was performed by applying the circular binary segmentation (CBS) algorithm available [7] in the R/Bioconductor package DNAcopy version 1.38.1. Segments with a mean log2 ratio deviating by more than 0.18 from the median log2 ratio calculated over all features were called chromosomal gain or loss, respectively. To identify regions of allelic imbalance and loss of heterozygosity (LOH), we used the approach of reference [8].

4.5 Integrative data analysis

Analyses were performed using R version 3 and included univariate association tests, multivariate regression with and without lasso penalization, Cox regression, generalized linear models, principal component analysis and clustering. We used a model-free data analysis approach that did not rely on fitting parametric response curve models. Generally, we considered the data from each concentration separately to allow for dose-dependent target specificity. For some visualizations and lasso models (as indicated), we used averages of responses either at all concentrations, or at the two lowest concentrations for kinase inhibitors, where off-target activity at higher doses was a concern. For Figure 6a, the data for 17 drugs each at two concentrations were considered: for fludarabine and nutlin-3 at the two highest concentrations, for the other 15 at each of the two lowest concentrations. A version of the same visualization, using all drugs that passed quality control and showing more concentrations, is provided in Supplementary Fig. S8. For each column, median and 1.4826 times the median absolute deviation (i. e., a robust estimator of scale that coincides with the standard deviation in the case of normal data) were computed, and data in each column were centered and scaled by these values, resulting in robust z-scores. The heatmap was plotted using the CRAN package pheatmap. The rows (patients) were grouped by disease category. Within each group, an ordering of the rows was obtained from the dendrogram that resulted from hierarchical clustering with the Euclidean metric (R function *hclust*). Within-tree branch flips were permitted to arrange responders to BCR inhibitors towards the top. The columns were globally ordered using the dendrogram order produced by hclust with default branch arrangement.

4.6 Associations of ex-vivo drug responses with genomic features

We tested for associations between drug viability assay results and genomic features by Student's t-tests (two-sided, with equal variance). Each concentration was tested separately. We tested somatic mutations (aggregated at the gene level), copy number aberrations, methylation groups (low programmed: LP, intermediate programmed: IP, High programmed: HP) and IGHV status. We restricted the analysis to features that were present in at least 3 patient samples (63 features). p-values were adjusted for multiple testing by applying the Benjamini-Hochberg procedure. For Figure 9a and Supplementary Fig. S19 and S20 viabilities across different drug concentrations were aggregated using Tukey's median polish method.

4.7 Batch effects

The screen was performed in three groups of batches over a time period of 1.5 years. To control for confounding by the different batch groups we repeated the drug-feature association tests using batch group as a blocking factor and a two-way ANOVA test. We then compared the p-values from both tests (Supplementary Fig. S30). Only one drug, bortezomib, showed discrepant p-values, and exploration of its data suggested that it lost its

activity during storage. The data for this drug were not used for further analysis. For all remaining associations, testing with and without batch as a blocking factor yielded equivalent results. Therefore, all reported p-values for associations are taken from the t-tests without blocking for batch effects.

4.8 Gene expression and gene set enrichment analysis

For the $n = 123$ patient samples for which we had drug sensitivity data and RNA–Seq data, we searched for associations of these two data types. Using the DESeq2 method we regressed the RNA-Seq read count data onto the drug response groups (BTK, MEK, mTOR, weak responder). Differentially expressed genes between groups were selected (raw p-value < 0.05) and ranked by their test statistics. Parametric Analysis of Gene Set Enrichment (PAGE) [9] was applied to the ranked lists with the C6 and H gene set selections from the MSigDB database (`http://software.broadinstitute.org/gsea/msigdb`). The same procedure was used to identify gene expression signatures of CLL patient samples with trisomy12.

4.9 Survival analyses

Survival times were calculated from the time of sample collection to death (overall survival: OS) or to treatment (time to treatment: TTT). Follow-up information to calculate OS was available for all 184 CLL patients. For 174 of 184 CLL patients treatment information after sample collection was available. The impact of genomic features on survival endpoints was tested using the log-rank test. Impact of normalized drug responses as continuous variables on survival endpoints was calculated by univariate Cox regression modeling. Multivariate Cox regression modeling was performed to assess the impact of drug responses on survival endpoints in the context of important other covariates. For visualization purposes (i.e., not for inference), optimal cut-points of drug responses were calculated using maximally selected rank statistics as computed by the R/CRAN package maxstat [10]. Based on these cut points, patients were split into two groups, and their survival data were plotted using the Kaplan-Meier method.

4.10 Penalized multivariate regression

We performed multivariate regression to explain drug responses by the available potential predictors. We used a Gaussian linear model with L1-penalty (i.e., lasso regression) as implemented in the R package glmnet version 2.0 [11] with mixing parameter $alpha = 1$.
As the dependent variable, the average viability value across all 5 concentrations was used for the chemotherapeutics (fludarabine, doxorubicine and nutlin-3) and the average of the two lowest concentrations for the targeted drugs (ibrutinib, idelalisib, selumetinib, everolimus and PRT062607). As input to the model, the expression data were normalized and transformed using the *varianceStabilizingTransformation* function from DESeq2, and both expression and methylation data were filtered to include only the top 5000 most variable features each. Gene mutations were only included in the model if present in at least 5 samples. Features with more than 10% missing values were excluded, and only patients that were characterized in at least 90% of each data type's features were included in the model. Remaining missing values were imputed by the mean for methylation data and by the most common mutation status for genetic data. $n = 102$ samples were used for model fitting.
As predictors in the lasso model the genetic mutations ($p = 11$) and IGHV status (coded as 0-1), demographics (age, sex), pretreatment (coded as 0-1) and the top 20 principal components of gene expression and methylation data were used. All features were scaled to unit variance and mean zero before using lasso to achieve fair treatment of all predictors by the penalty constraint.
To compare explanatory power of different data sets (Figure 11), a separate model was fit including only predictors of one omic type at a time as well as a joint model including all predictors. Using 10-fold cross-validation the optimal penalty parameter λ was chosen to minimize the cross-validated R^2 of the model using the function *cv.glmnet*. As a measure of explained variance the reduction in cross-validated mean squared error relative to the null model was calculated. For single features, i.e., IGHV, the R^2 from a standard linear model was used as corresponding quantity. Mean and standard error of the explained fraction of variance were obtained from 100 repetitions of cross-validation.
For the models shown Figure 12, only genetic features, IGHV status, pretreatment (coded as 0, 1) and methylation cluster (coded as 0, 0.5, 1) were considered, which resulted in $n = 168$ cases and $p = 13$ features after removal of features with more than 10% missing values. Remaining missing values were assumed to be 0. Adaptive lasso [12] was used to consistently select genetic features that modulate drug response. The model was fit using the function *glmnet* without standardization of the predictors and with penalty factors given by the inverse of the absolute coefficients from a linear model fit. Using 10-times repetition of 10-fold cross-validation, the optimal penalty parameter λ was chosen to minimize the cross-validated R^2 of the model using the function *cvr.glmnet* from the R package ipflasso.

5 Supplementary figures

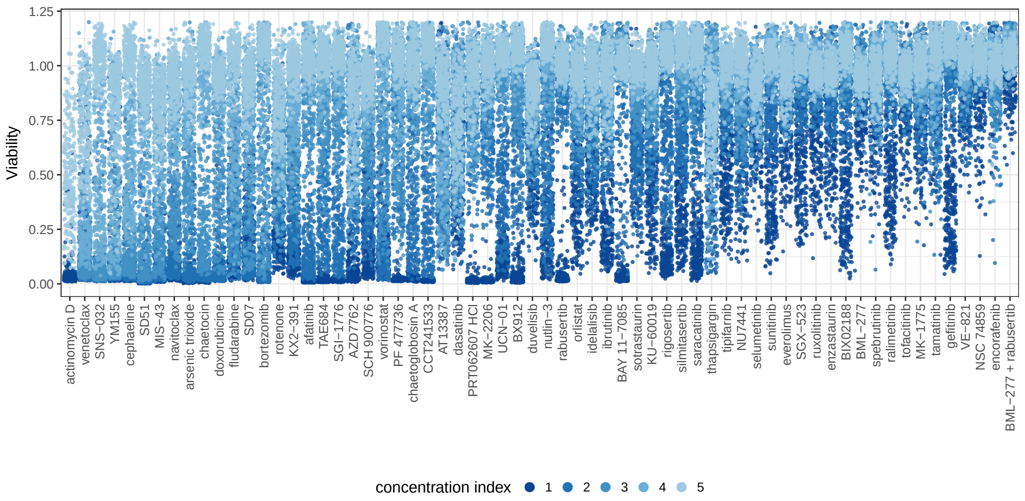

Figure S1: **Drug induced effects on cell viability.**
Relative cell viability of 246 primary tumor samples, compared to negative control, after treatment with $n = 64$ drugs at 5 concentrations each is shown along the y-axis. Drug concentrations are color coded, highest concentration: 1, lowest concentration: 5. The plot shows high variability of effects between different drugs, from mostly lethal (left) to mostly neutral (right), concentration dependence of effects and high variability of effects of the same drug/concentration across patients.

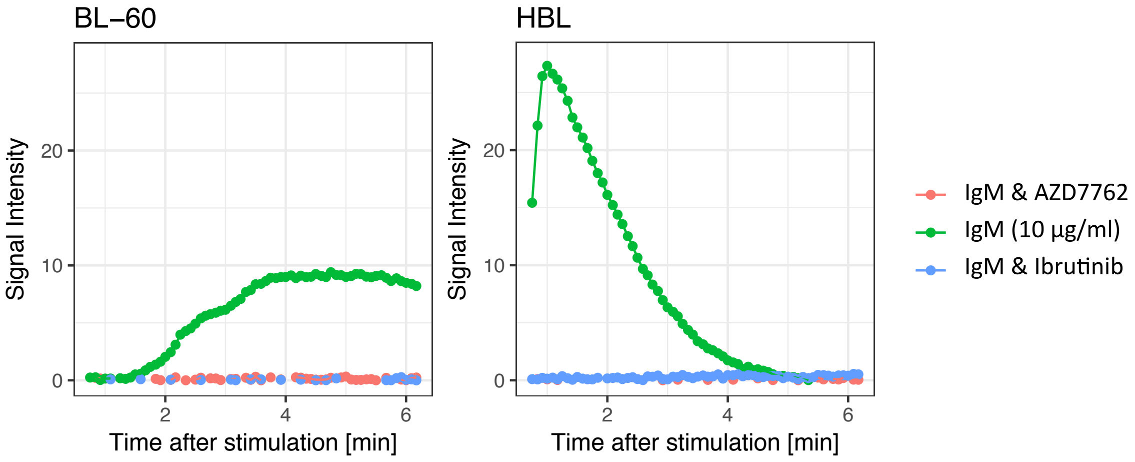

Figure S2: **Inhibition of calcium release after IgM stimulation.**
BL-60 and HBL-2 cells were stained with Fluo-8 (abcam), a calcium sensitive fluorescence dye. 5×10^4 cells/well were re-suspended in RPMI containing 1% FBS and stained according to the manufacturer's instructions. After pre-incubation with ibrutinib (500nM) and AZD7762 (500nM), cells were imaged over a period of 6.3 minutes with a Zeiss cell observer microscope and a 2.5x objective. IgM (BD) was added to achieve a final concentration of 10 $\mu g/ml$. A constant frame of 180.6 μm^2 was chosen and the mean intensity for each frame and time point was calculated. Both ibrutinib and AZD7762 blocked calcium mobilization after IgM stimulation.

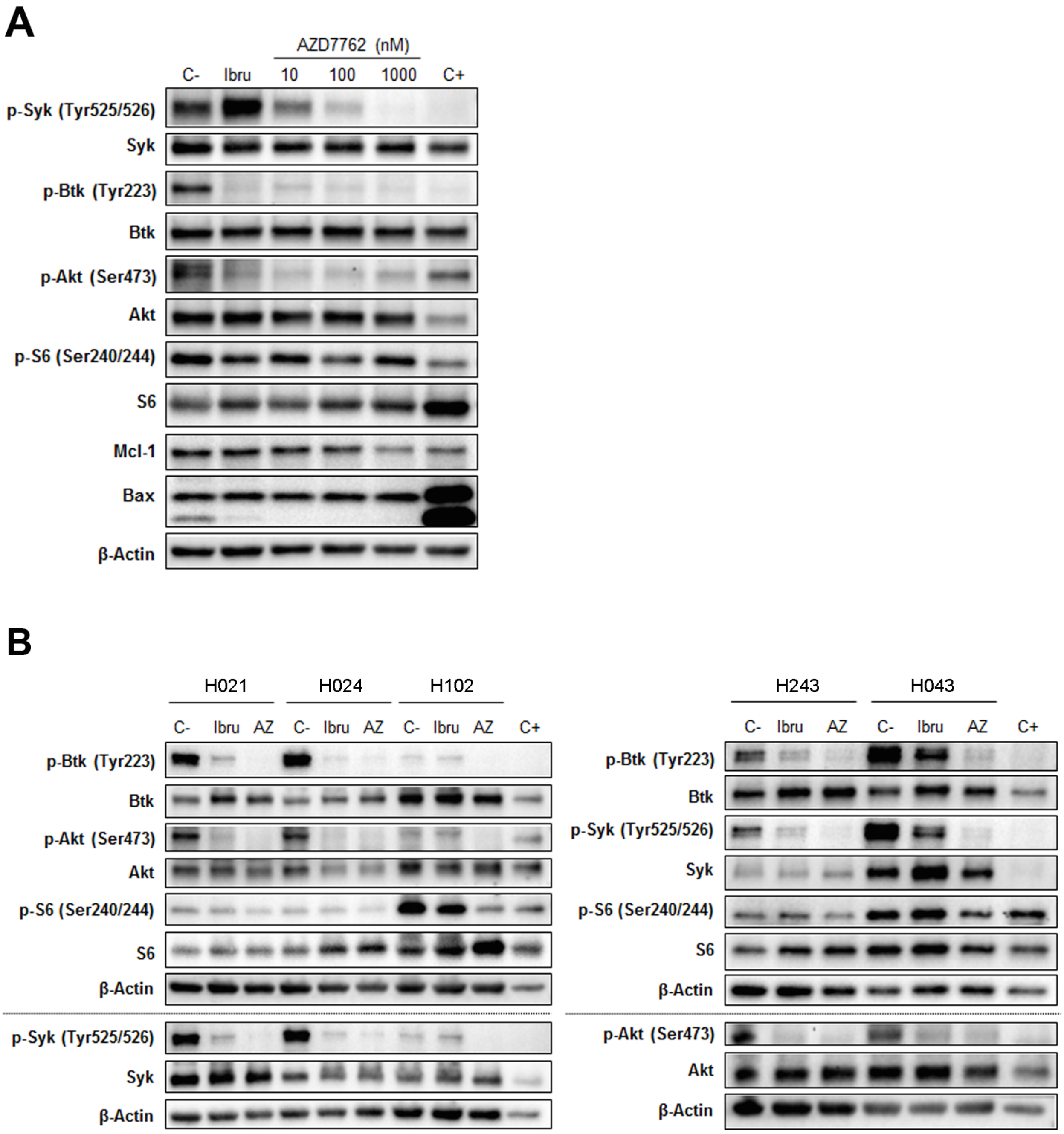

Figure S3: **Effect of AZD7762 on key signaling cascade members of the B-cell receptor pathway.** AZD7762 inhibited the phosphorylation of proteins involved in BCR signaling. Western blot analysis of the human mantle cell lymphoma cell line HBL-2 **(A)** and primary CLL cells **(B)** after 4 h treatment with AZD7762. DMSO, 0.001% (v/v), was used as vehicle control (C-), and cell lysate of GRANTA was used as antibody control (C+). Primary samples were treated with 500 nM AZD7762 (AZ) or Ibrutinib (Ibru): 500 nM. Data shown in **(A)** are representative of three independent experiments. Blots shown in **(B)** are the result of a single experiment. For each condition 15 μg of protein lysate were loaded. Membranes were probed with phospho- and total protein specific antibodies as indicated.

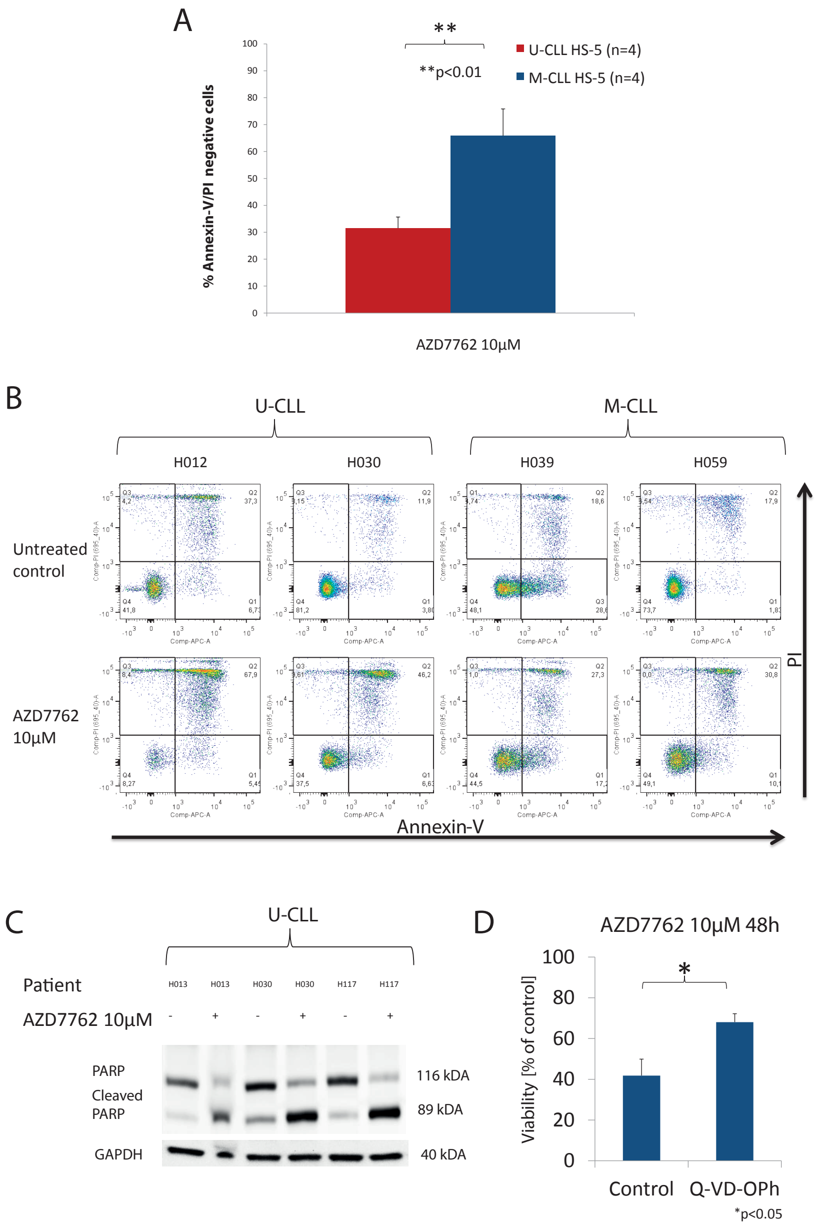

Figure S4: **AZD7762 induces apoptosis of primary CLL cells.**

Panel A) shows AZD7762 (CHEK1/2 inhibitor) induced apoptosis in primary CLL cells co-cultured with stroma cells (HS-5 cells). Apoptosis induction was measured after 48 h of drug exposure by flow cytometry and annexin-V / propidium iodid (PI) stains. Data was normalized to untreated controls. U-CLL ($n = 4$) patient samples are more sensitive to AZD7762 than M-CLL ($n = 4$) patient samples ($p < 0.01$).

Panel B) shows examples of FACS plots for U-CLL ($n = 2$) and M-CLL ($n = 2$) patient samples treated with 10 μM AZD7762 and untreated controls).

Panel C) shows western blots demonstrating PARP cleavage in U-CLL patient samples ($n = 3$) exposed to AZD7762, as a sign of apoptosis induction.

Panel D) shows U-CLL patient samples treated for 48 h with 10 μM Q-VD-OPh (pan-caspase inhibitor) and/or 10 μM AZD7762. Apoptosis induction of AZD7762 could be in part inhibited by the pan-caspase inhibitor Q-VD-OPh, which indicates that the majority of AZD7762 mediated cell death is through apoptosis.

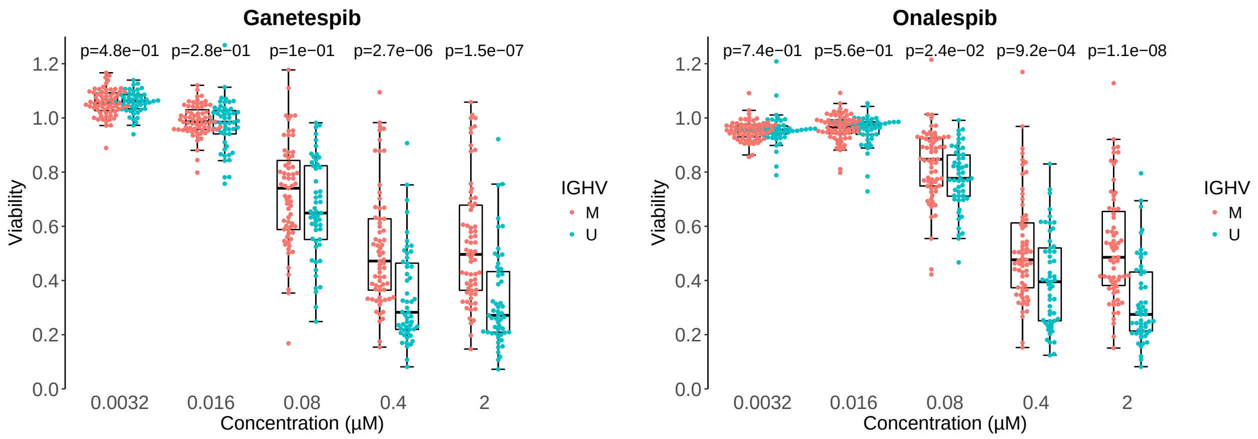

Figure S5: **Association between the response to HSP90 inhibitors and IGHV status.** CLL patient samples (n=120) were treated with Ganetespib or Onalespib and studied for drug response ex-vivo. After treatment with five different concentrations for 48 hours, viability of CLL cells was measured with the CellTiterGlo assay. Viabilities are shown for IGHV mutated (M) and IGHV unmutated (U) CLL samples.

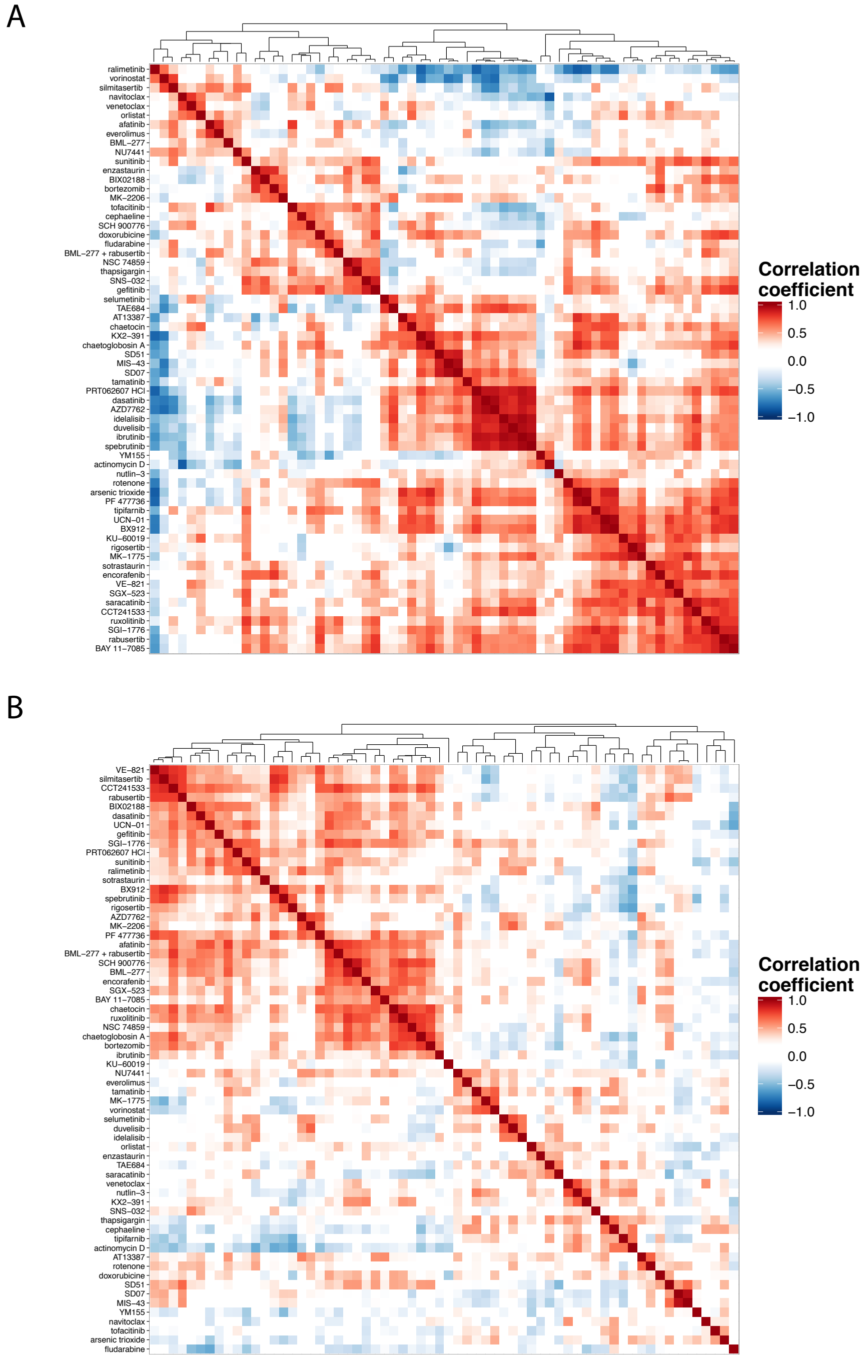

Figure S6: **Drug-drug correlations in MCL and T-PLL.**
The heatmaps show the drug-drug correlation matrices for all pairs of drugs for MCL and T-PLL. They complement Figure 3 in the main text. Pearson correlation coefficients were computed from the average of the drug responses at the two lowest concentration steps, within the subset of **(Panel A)** MCL patient samples ($n = 10$) and **(Panel B)** T-PLL patient samples ($n = 25$). Clusters of drugs with high correlation and anti-correlation are shown by red and blue squares, respectively. In MCL, the major clusters include: (I) kinase inhibitors of the B cell receptor, incl. idelalisib (PI3K), ibrutinib (BTK), duvelisib (PI3K), PRT062607 (SYK); (II) inhibitors of redox signaling / reactive oxygen species (MIS-43, SD07, SD51) and (III) BH3 mimetics (navitoclax, venetoclax). In T-PLL, the correlations are less pronounced, e.g., BTKi and PI3Ki correlations are largely lost.

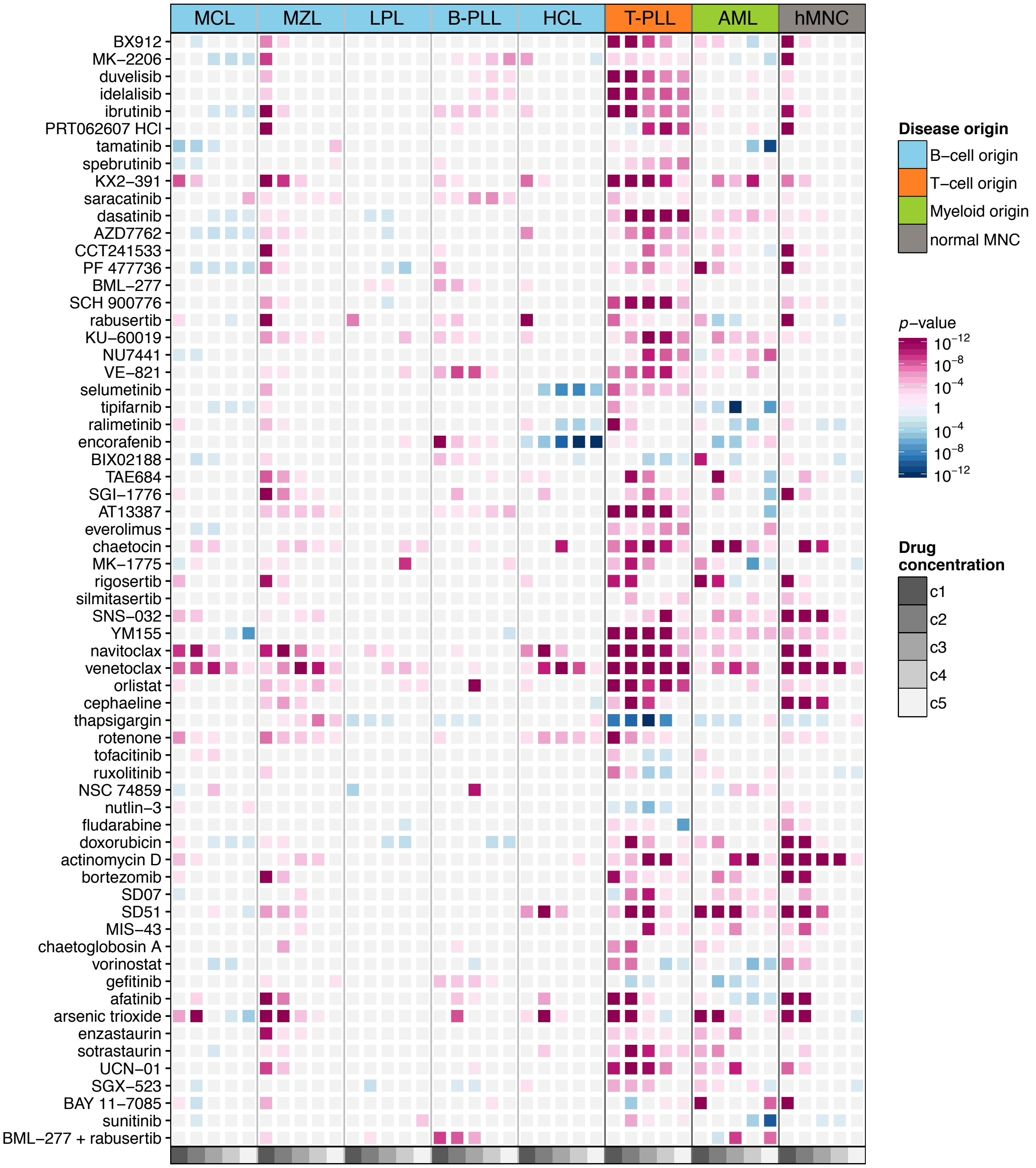

Figure S7: **Summary of disease-specific drug effects.**
The heatmap shows significant differences in drug responses of CLL samples (used as a common reference; $n = 184$) versus MCL: mantle cell lymphoma ($n = 10$), MZL: marginal zone lymphoma ($n = 6$), LPL: lymphplasmocytic lymphoma ($n = 4$), B-PLL: B-Prolymphocytic leukemia ($n = 3$), HCL: hairy cell leukemia ($n = 3$), T-PLL: T-Prolymphocytic leukemia ($n = 25$), AML: acute myeloid leukemia ($n = 5$) and hMNC: human mononuclear cells ($n = 3$). Drug responses between patient groups were compared with t-tests (null hypothesis: no difference), and two-sided p-values were computed. Multiple testing was accounted for with the Benjamini-Hochberg method, so that the rejection threshold corresponded to a false discovery rate (FDR) of 10%. Significant associations according to this threshold are shown with the color scale, where hue indicates the sign of the difference (pink: less, blue: more sensitive compared to CLL) and intensity the raw p-value, as indicated by the color key. Association tests for which the null hypothesis was not rejected are shown in light grey. The five columns within each block correspond to the five concentrations tested (c1: highest, c5: lowest, see Table S2).

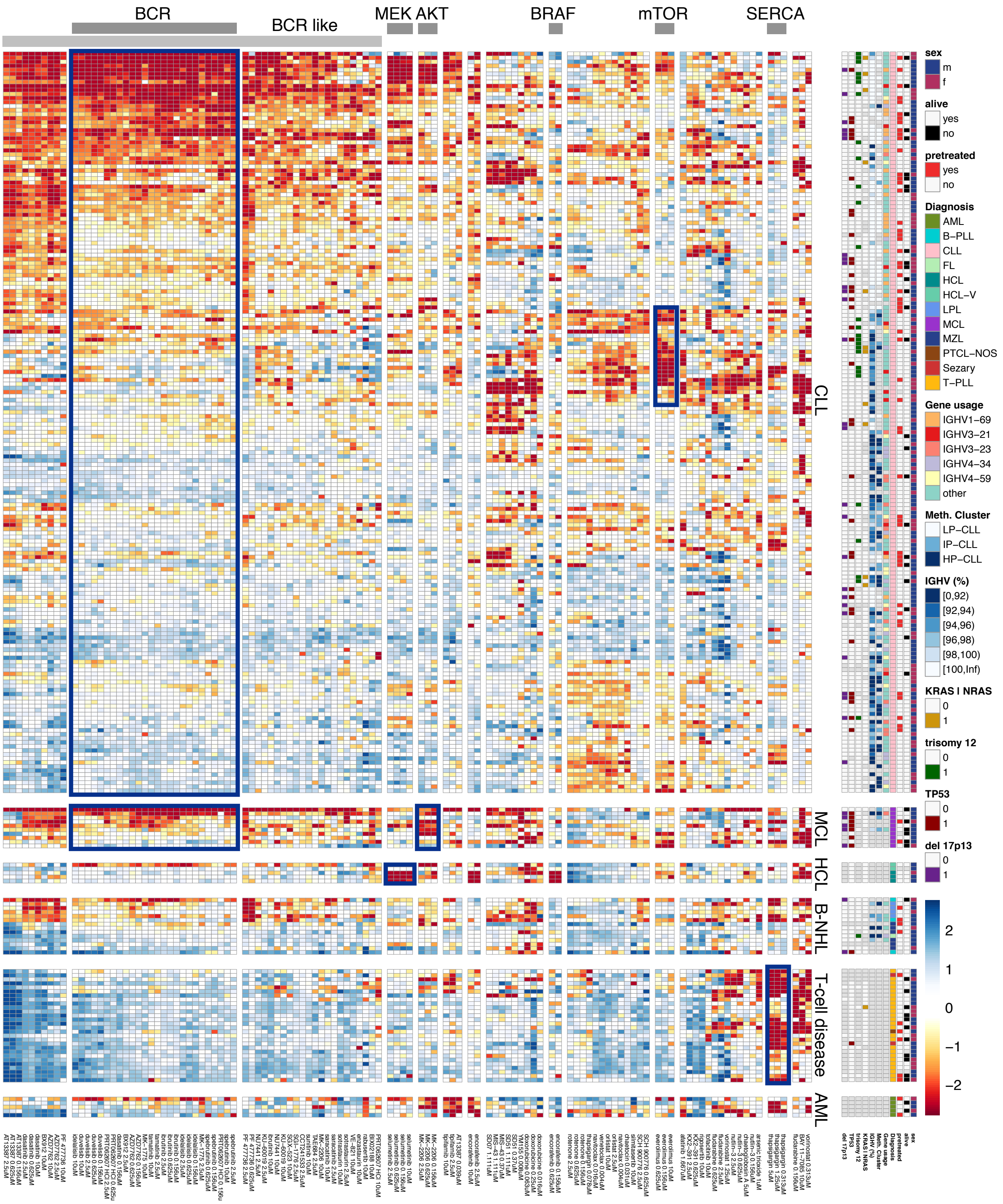

Figure S8: **Global overview of drug response landscape.**
The heatmap provides the drug response landscape for the 246 blood cancer samples. The viability measurements are shown on a z-score scale: for each column (response profile of a drug at one concentration), data were centered and scaled. 116 columns corresponding to a total of 53 drugs are shown (for the purpose of this visualization, 204 columns with insufficient variation across samples were omitted). The color bars to the right show sample annotations. The ordering of the samples and drugs was induced by a hierarchical clustering dendrogram (Euclidean metric, complete linkage). Prior to clustering, samples were divided into six disease groups, indicated by the horizontal gaps. Drug response based clustering indicates the existence of functionally defined disease subgroups.

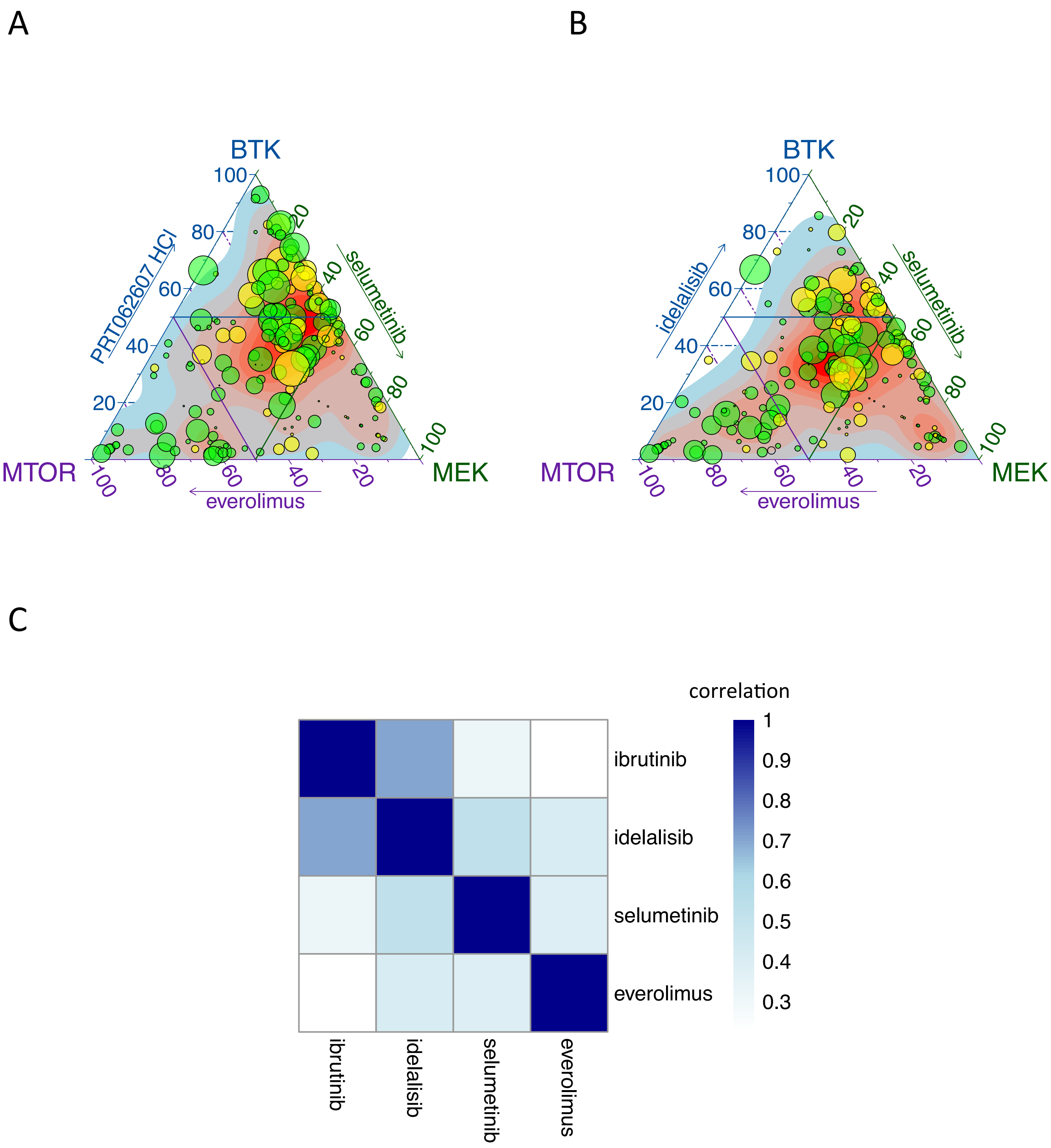

Figure S9: **Relative effects of BCR inhibitors (SYK, PI3K), everolimus (mTOR) and selumetinib (MEK) on cell viability and gene expression.**
Similar to Figure 6b in the main manuscript, but with PRT062607 HCl (SYK inhibitor, **Panel A**) and idelalisib (PI3K inhibitor, **Panel B**) instead of ibrutinib (184 CLL samples). Green dots represent pretreated samples. **Panel C)** Comparison of gene expression responses to drug treatments. 12 CLL samples (6 M-CLL and 6 U-CLL) were treated with ibrutinib, idelalisib, selumetinib, everolimus and negative control. Gene expression profiling was performed after 12 hours of drug incubation using Illumina microarrays. For each sample, drug and gene, the logarithmic fold change (LFC) between treatment and negative control was calculated, and the 2000 genes with highest median absolute LFC across samples and drugs were selected. LFCs were median-summarized across samples. For each pair of drugs, the correlation coefficient across the 2000 genes was calculated. The resulting correlation matrix is shown with the color code. The gene expression changes induced by idelalisib and ibrutinib were similar, whereas selumetinib and everolimus each led to expression changes of different genes.

A

B

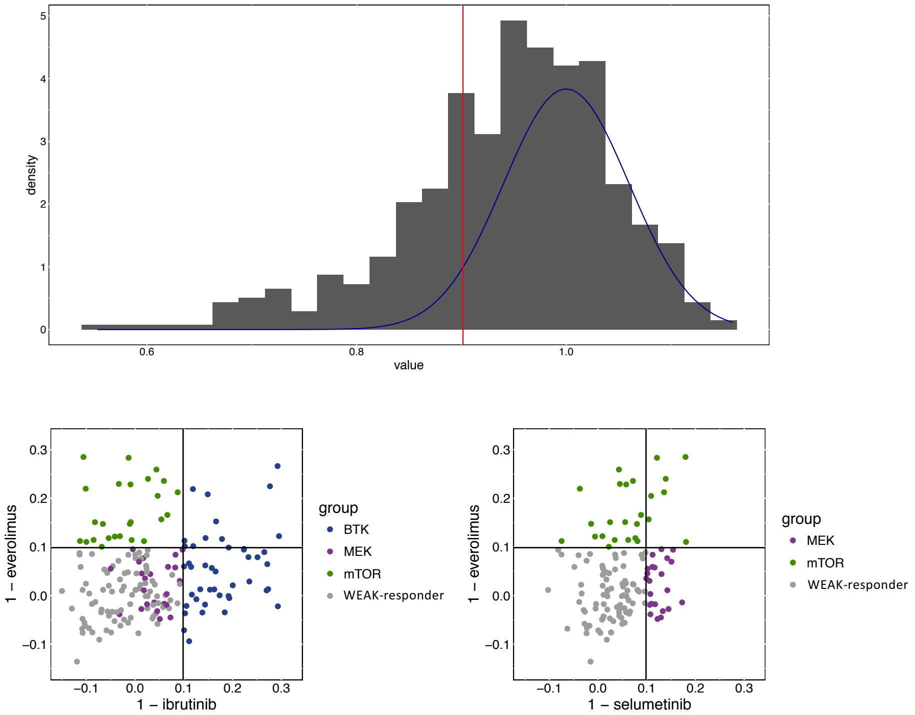

Figure S10: **Derivation of thresholds for the decision tree model.**
Panel A) For each of the drugs ibrutinib (targeting BTK), everolimus (mTOR) and selumetinib (MEK) we used the mean values from concentrations 156 nM and 625 nM and considered the distribution of these values across all CLL samples, shown in the histogram. In A) the histogram shows exemplarily the response to ibrutinib. We assumed that this distribution was a mixture of two components: (1) a null distribution (corresponding to no or negligible response to the drug), which is symmetric about 1, and (2) an alternative distribution (responders), which has negligible mass above 1. We then used the *mirror method*, as follows, to derive a common threshold for response versus weak response for all three drugs. First, we computed the standard deviation σ of the values ≥ 1, with mean fixed to $\mu = 1$. The corresponding normal density is shown by the blue line. The observed values x were transformed into z-scores $z = (x - \mu)/\sigma$. If the assumption is made that the fitted normal density (blue line) is a good approximation of the null distribution, the z-score can be interpreted as a probability, namely the false positive rate (FPR) via the relation FPR $= 1 - F_{\mathtt{N}}(z)$, where $F_{\mathtt{N}}$ is the standard normal distribution function. Setting the target FPR to 0.05 we derived the threshold of 0.9014 (vertical red line).
Panel B) Two-dimensional inverted scatterplots for response to ibrutinib versus everolimus, and selumetinib versus everolimus. Each point corresponds to a patient sample ($n = 184$). The common threshold that we used to separate responders from weak-/non-responders is shown by the black lines. The axes are inverted and show 1-viability, hence the threshold 0.9014 derived in Panel A corresponds to 0.0986 in this representation. Weak responders (lower left quadrant) are color coded in grey, responders in green (mTOR), blue (BTK) or purple (MEK).

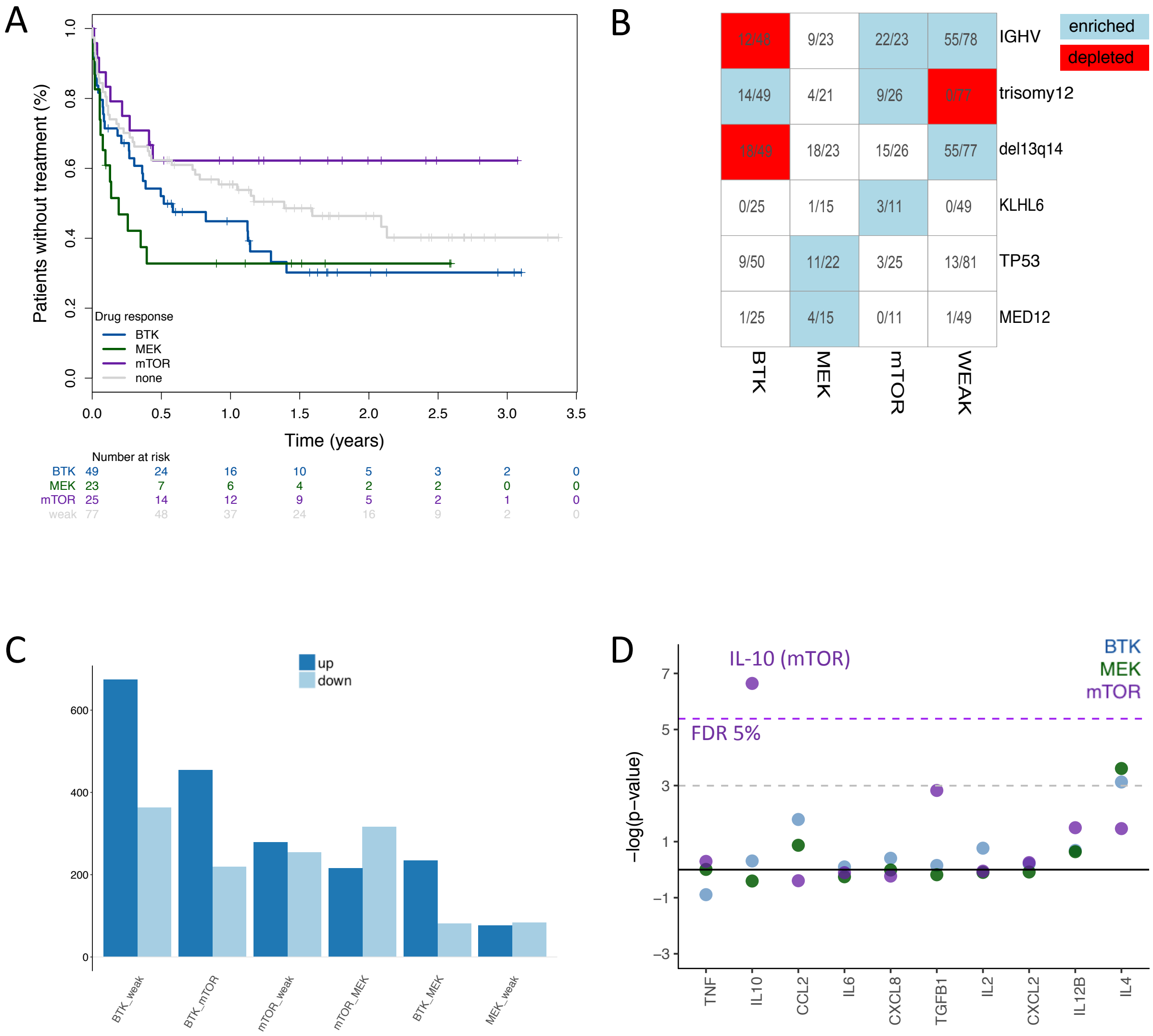

Figure S11: **Characterization of drug response groups in CLL.**
Panel A) Kaplan-Meier plot for time from sample collection to next treatment for the four groups. Patients in the mTOR-group had a longer time to next treatment (TTT) than patients in the BTK-group (p=0.05) and MEK-group (p=0.02). Other comparisons were not statistically significant. **Panel B)** Incidence of somatic gene mutations and CNVs in the four groups. Enrichment and depletion were assessed using Fisher-tests, and associations with p-value < 0.05 are highlighted by colors. **Panel C)** Number of differentially expressed genes between drug response groups. Differentially expressed genes were calculated by the DESeq2 method (FDR 10%). These genes were largely not overlapping. A gene enrichment analysis for differentially expressed genes is provided in S12 and S13. **Panel D)** Significantly (FDR 5%) increased IL-10 mRNA expression in mTOR group patients (purple). Other important cytokines / chemokines were not differentially expressed.

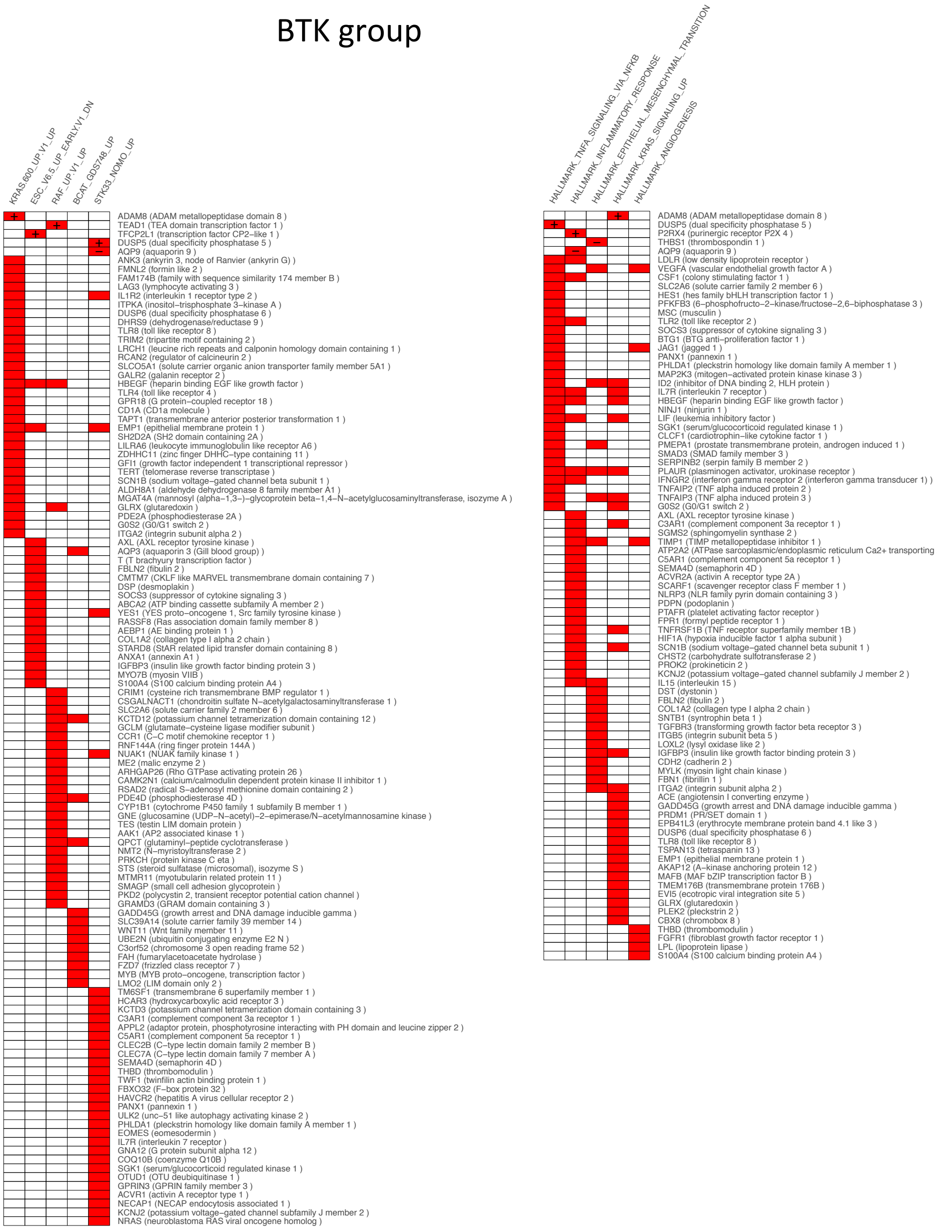

Figure S12: **Gene expression associated with the BTK group.**
Using the RNAseq data available for patient samples, differentially expressed genes between the BTK group ($n = 30$) and weak responders ($n = 59$) were identified and subjected to gene enrichment analysis using the MSigDB gene set collections *C6* and *Hallmark* (`http://software.broadinstitute.org/gsea/msigdb`). The top five enriched gene sets of the two gene set collections are shown together with the differentially expressed genes they contain. Differential gene expression analysis was performed with *DESeq2* using a loose threshold ($p<0.05$) [6]. Gene enrichment analysis was performed on the ranked gene lists using the Parametric Analysis of Gene Set Enrichment (PAGE) [9]. For each of the shown genes, we also computed the Pearson correlation coefficient with response to ibrutinib, within the BTK group samples; as well as the correlation test p-value. Genes for which $p<0.05$ and the absolute correlation coefficient >0.5 are marked with a $+$ or $-$ (depending on the sign of the coefficient). In this figure, the number of these genes is small and may potentially be explainable by multiple testing; a comparable analysis was performed for genes associated with the mTOR group in Figure S13, where the frequency of such genes is higher in **Panel B**.

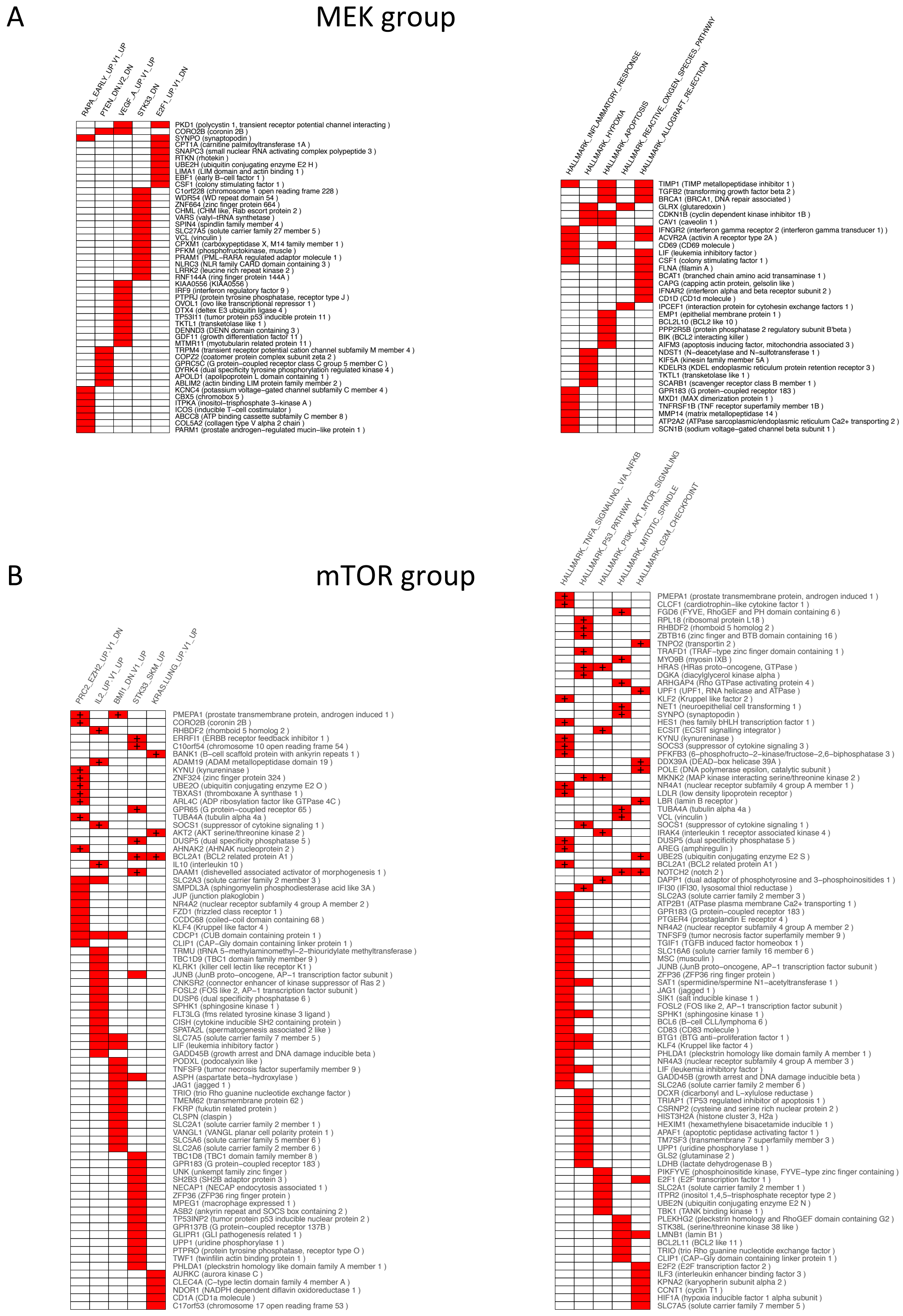

Figure S13: **Gene expression associated with the MEK and mTOR groups.**
Similar to Figure S12 for the MEK group ($n = 18$) vs. weak responders ($n = 59$) **(Panel A)** and the mTOR group ($n = 15$) vs. weak responders **(Panel B)**. The correlation tests were performed with respect to selumetinib **(Panel A)** and everolimus **(Panel B)**.

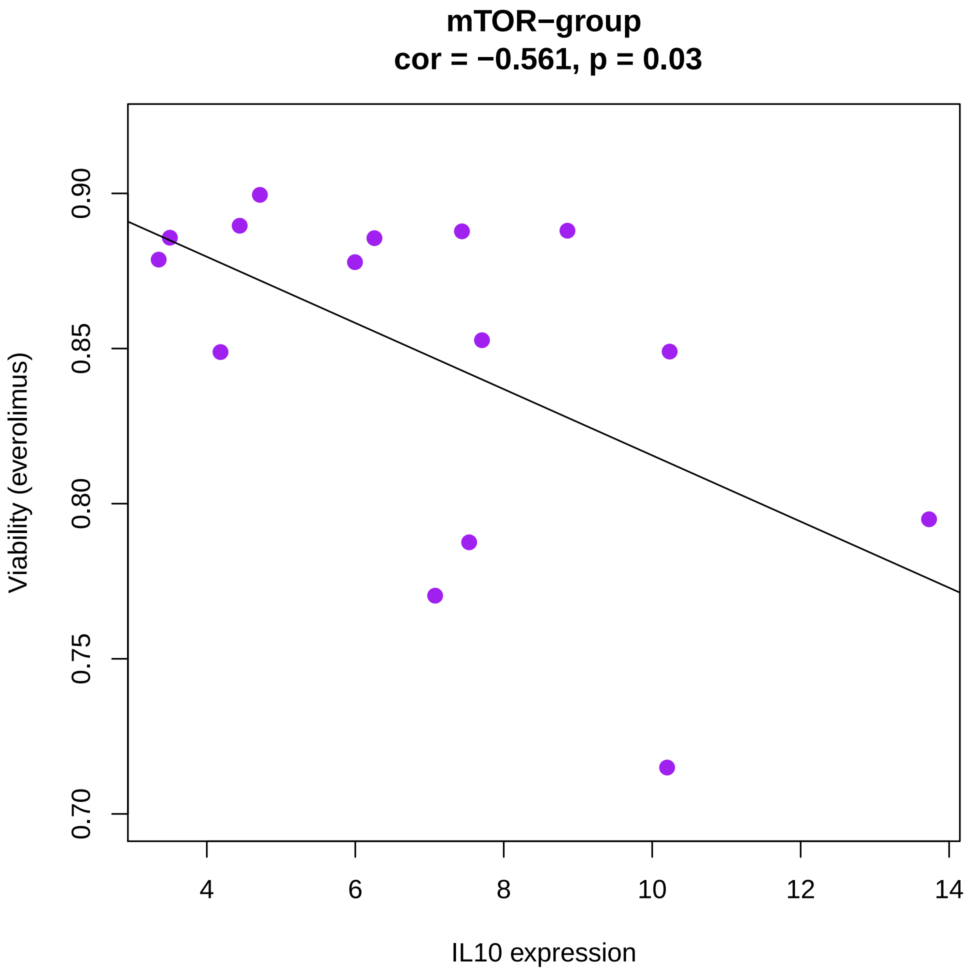

Figure S14: **Correlation of IL-10 mRNA expression and response to everolimus within the mTOR group.** Within the mTOR group ($n = 15$) we correlated IL-10 mRNA expression with response to everolimus. Cases with a better response to everolimus had a higher expression of IL-10. IL-10 mRNA expression shows normalized read counts on a logarithmic (base 2) scale. The *DESeq2* package was used for library size normalization and variance stabilizing transformation [6].

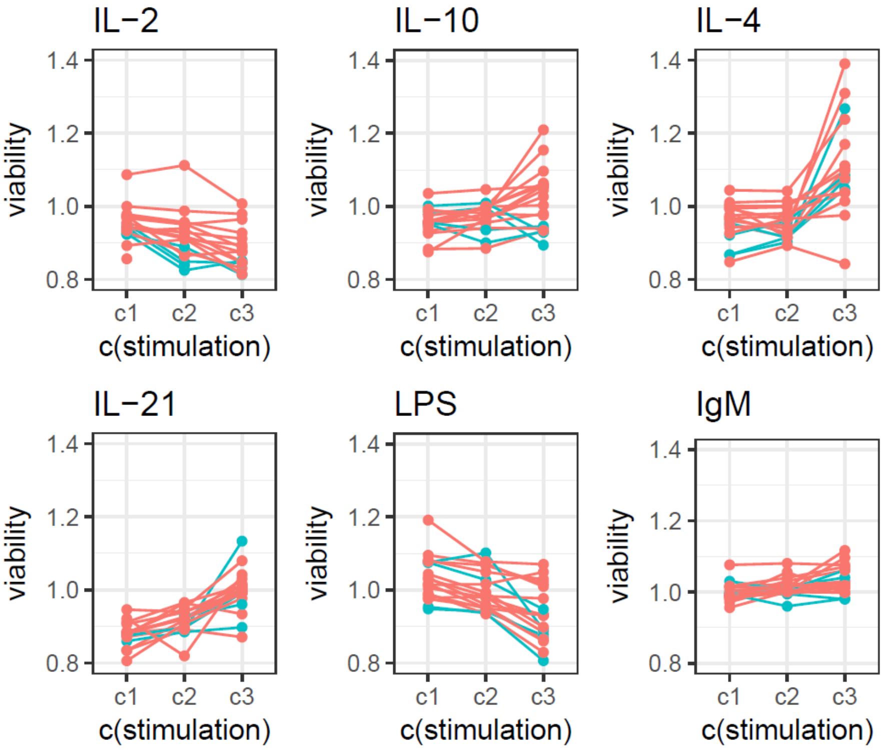

Figure S15: **Response to cytokines in CLL.**
18 CLL patient samples were exposed to IL-2, IL-10, IL-4, IL-21 (c1=0.001, c2=0.1, c3=10 ng/μl), LPS (c1=1, c2=10, c3=100 ng/μl) and IgM (c1=10 nM, c2=1, c3 = 10 μM) for 48 hours. Viability was measured using a CellTitre Glo assay, and luminescence was normalized to unstimulated controls. The blue lines mark samples from the mTOR group. IL-10 had a pro-survival effect on the majority of samples (*t*-test, c1 vs. c3 $p=0.009$), but not on those in the mTOR group. IL-4 and IL-21 had pro-survival effects on most samples, including the mTOR group (*t*-test, c1 vs. c3 $p<0.001$).

A

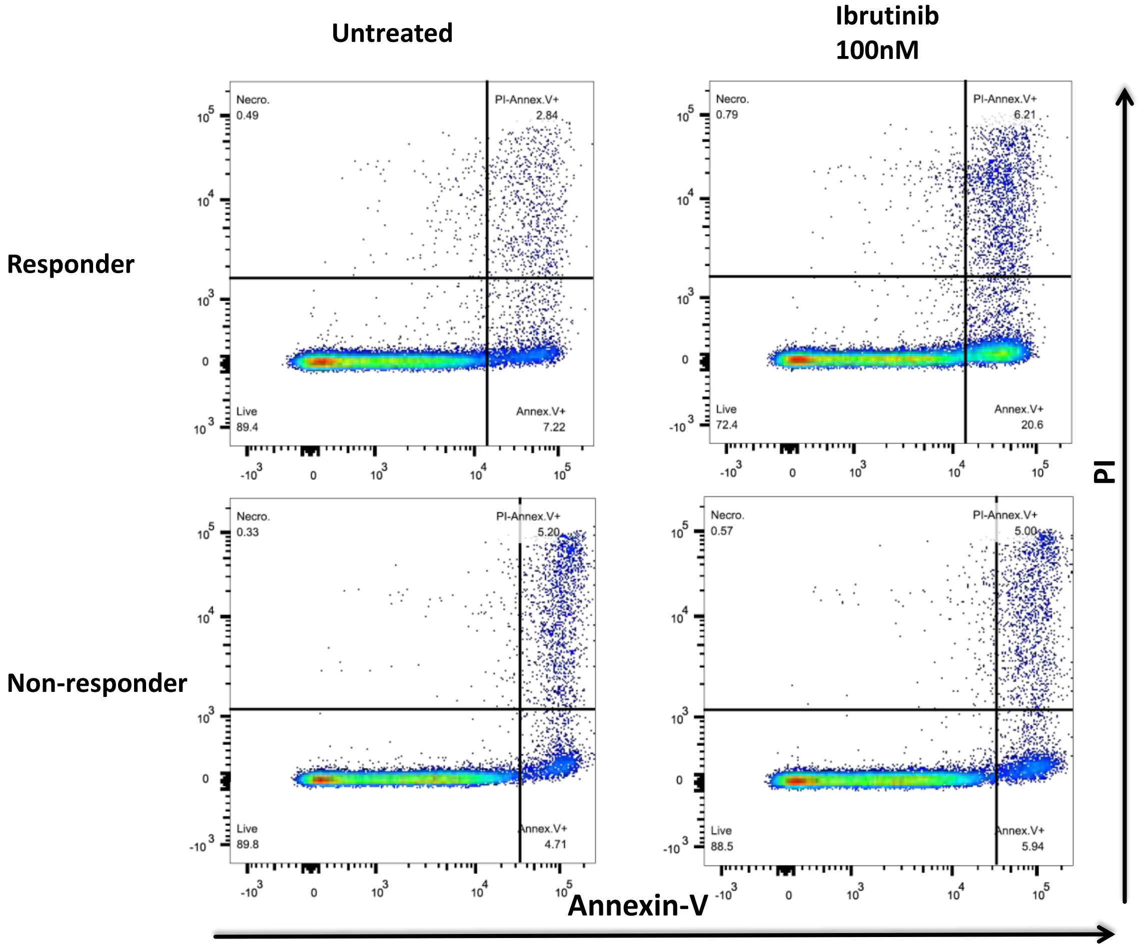

B

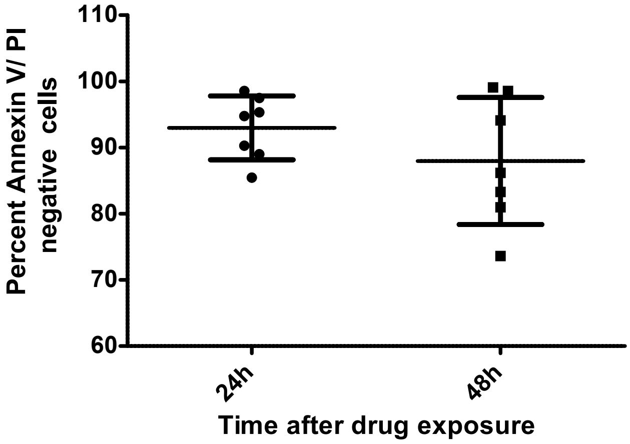

Figure S16: **Ibrutinib induces apoptosis in primary CLL cells.**
Panel A shows representative flow cytometry plots after drug exposure to 100 nM ibrutinib and for untreated controls. Apoptosis induction was measured by annexin-V staining, and cell death was quantified by propidium iodide (PI) staining.
Panel B summarizes the quantification of viable, i.e., annexin-V and PI negative, cells after 24 h and 48 h drug exposure. These results are in agreement with the results based on the ATP-based (cellTiter Glo) viability measurements.

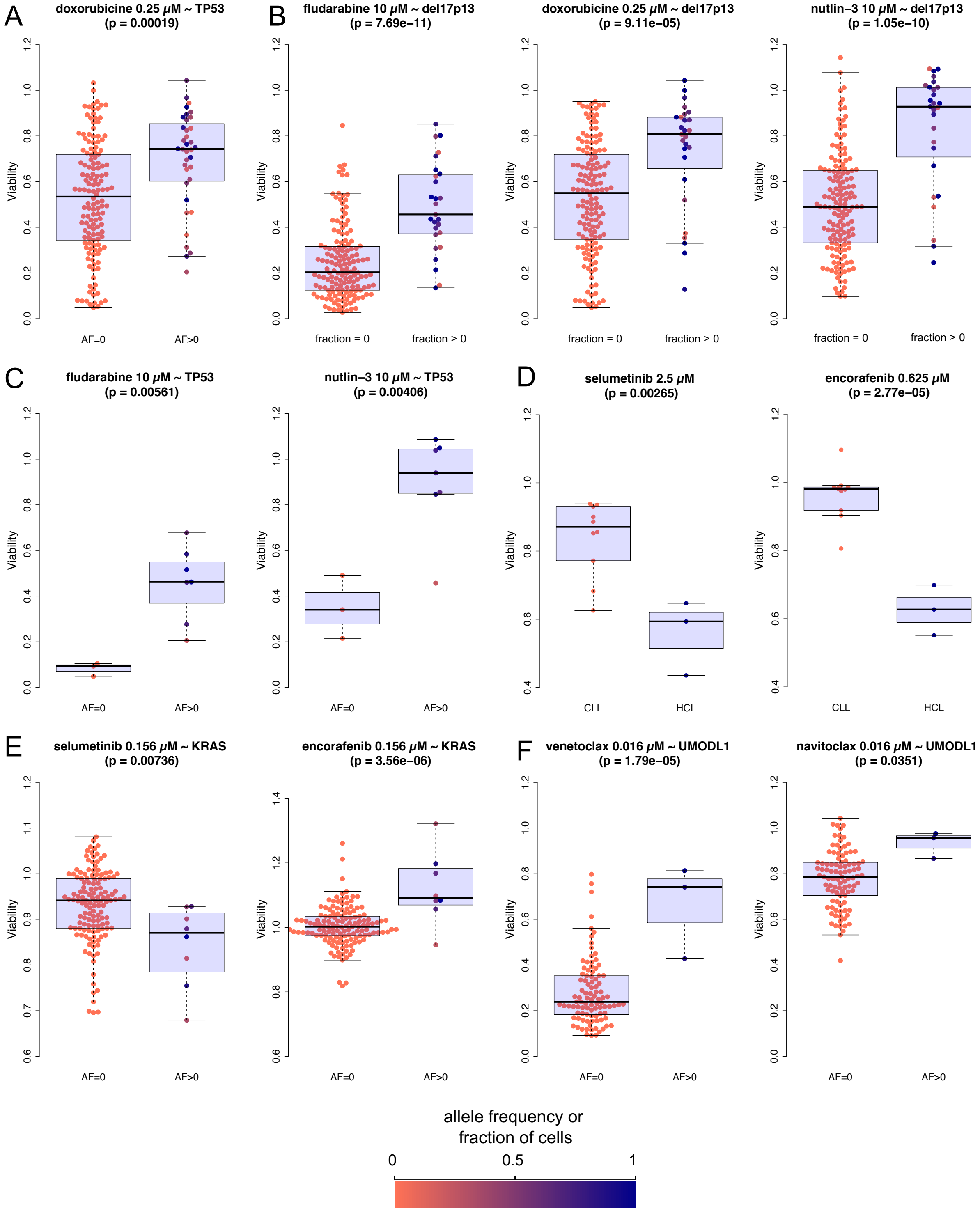

Figure S17: **Effect of mutations on drug response.** Exemplary beeswarm plots for associations between drugs and gene mutations or structural variants are shown. p-values were calculated with two-sided *t*-tests. Observed allele frequency (AF), a measure of clonality of the mutation, or fractions of cells are shown using the color code. Panels **A**, **B**, **E**, **F** show CLL samples, **C** mantle cell lymphoma (MCL), **D** CLL (n=10) and hairy cell leukemia (HCL, n=3) with BRAF mutation are shown.

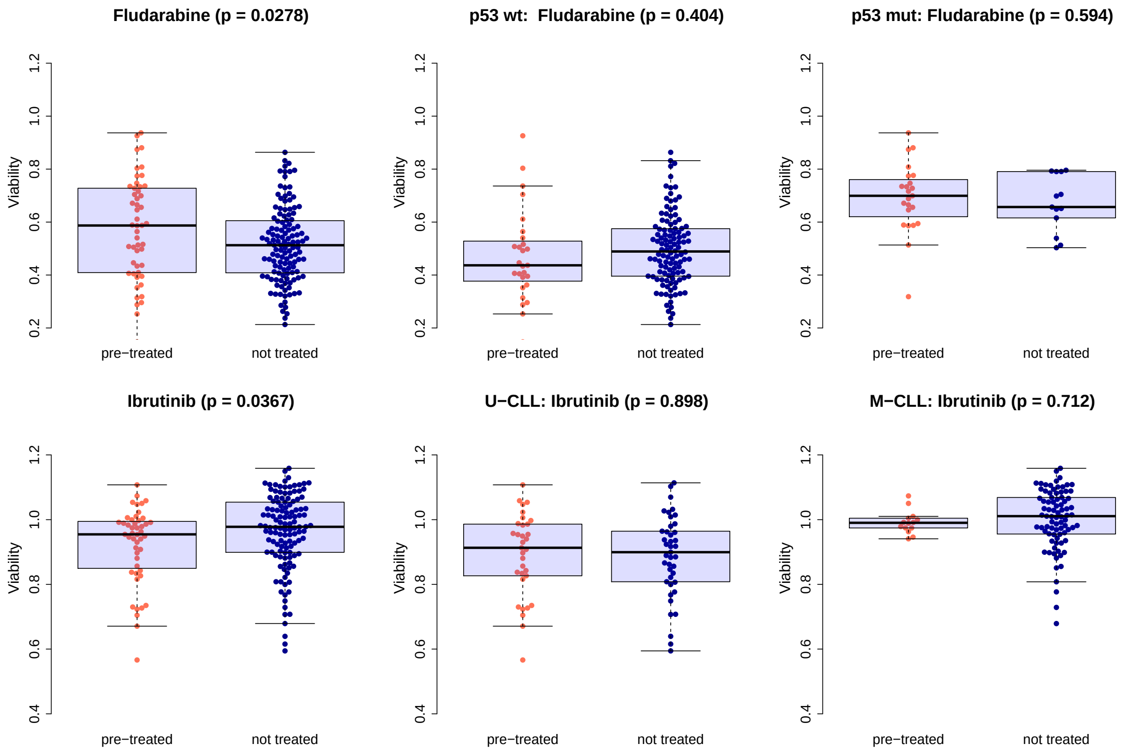

Figure S18: **Drug response in CLL stratified by pretreatment.**
Drug response in CLL stratified by pretreatment, *TP53* and IGHV status (Student's *t*-test). While pretreatment is associated with decreased response to fludarabine and increased response to ibrutinib, these apparent associations are driven by underlying causal associations with high risk genetics: *TP53* for fludarabine, IGHV mutation status for ibrutinib. After stratification into these genetically defined subgroups, there is no association with pretreatment status.

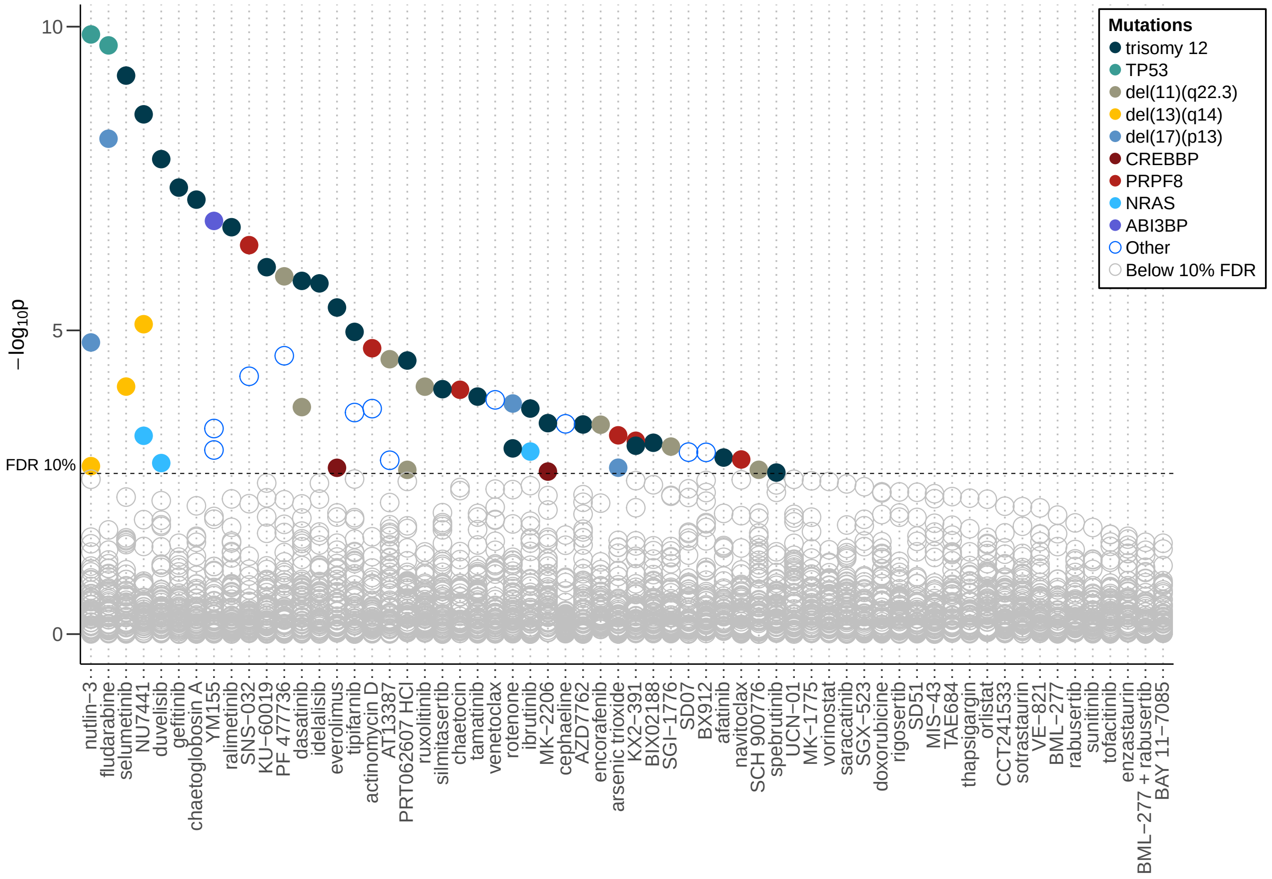

Figure S19: **Associations of drug sensitivities with gene mutations and structural aberrations in CLL.**
Each point represents a test for a drug-gene interaction. The drug is indicated by the points' position on the x-axis, the mutation by the color key (significant associations only). The y-axis shows the test p-values on a logarithmic scale. A simple two-group ANOVA was performed (as in figure 9 of the main text) but in contrast to the analysis for main figure 9 we additionally accounted for pretreatment status by including it as a second covariate. Multiple testing adjustment for FDR control was achieved by applying the method of Benjamini and Hochberg to all test p-values. For this analysis, the viability measurements for different drug concentrations were aggregated using Tukey's median polish method.

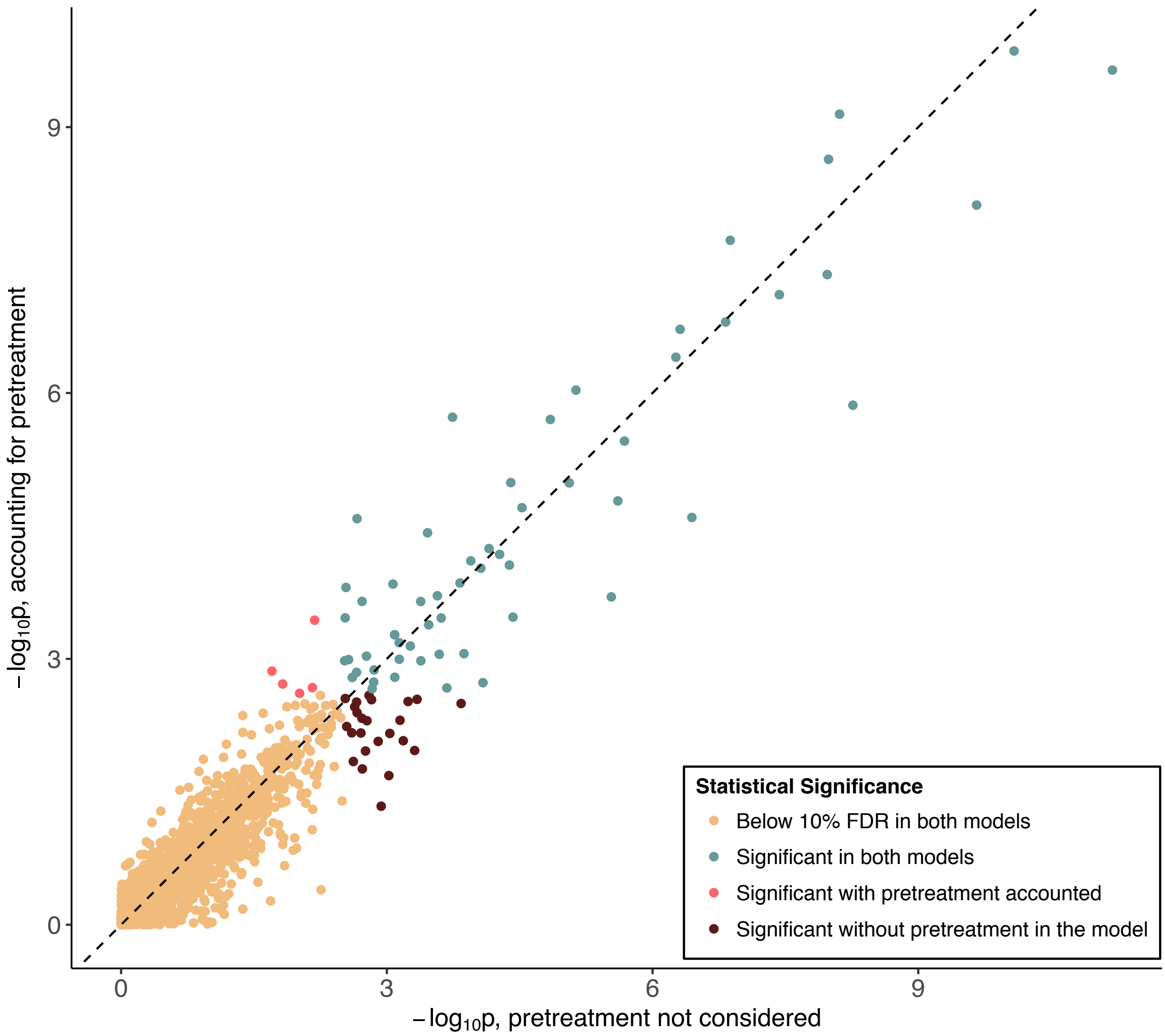

Figure S20: **Impact of pretreatment on associations of drug sensitivities with gene mutations.** We compared p-values between the models described in supplementary S19 and main figure 9. A scatter plot of this comparison in terms of the resulting association test p-values is shown. The scatter plot indicates that the two analyses are highly concordant.

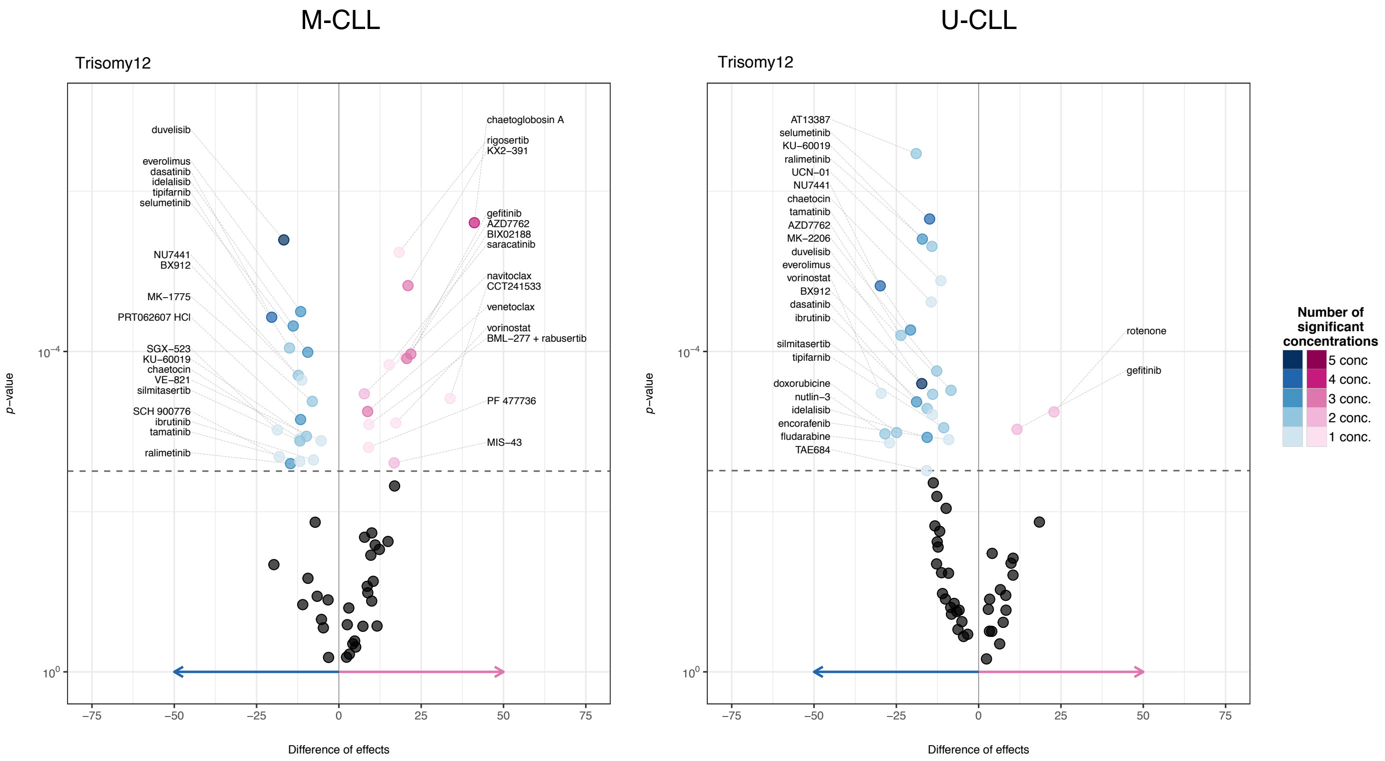

Figure S21: **Drug response associated with trisomy 12 within U-CLL and M-CLL.** The volcano plots show drug response differences between CLL patient samples with and without trisomy 12 as in Figure 10b of the main text but here separately for M-CLL (left; $n = 92$: 15 and 77 with and without trisomy 12, respectively) and U-CLL (right, $n = 69$: 11 and 58 with and without trisomy 12, respectively). The p-values (y-axis) were obtained from Student t-tests. The Benjamini-Hochberg procedure for multiple testing was applied to the p-values, and the dashed horizontal line corresponds to a false discovery rate of 10%. For each drug, several drug concentrations were tested, and we show the maximum difference in viability effect between the two tested groups (x-axis).

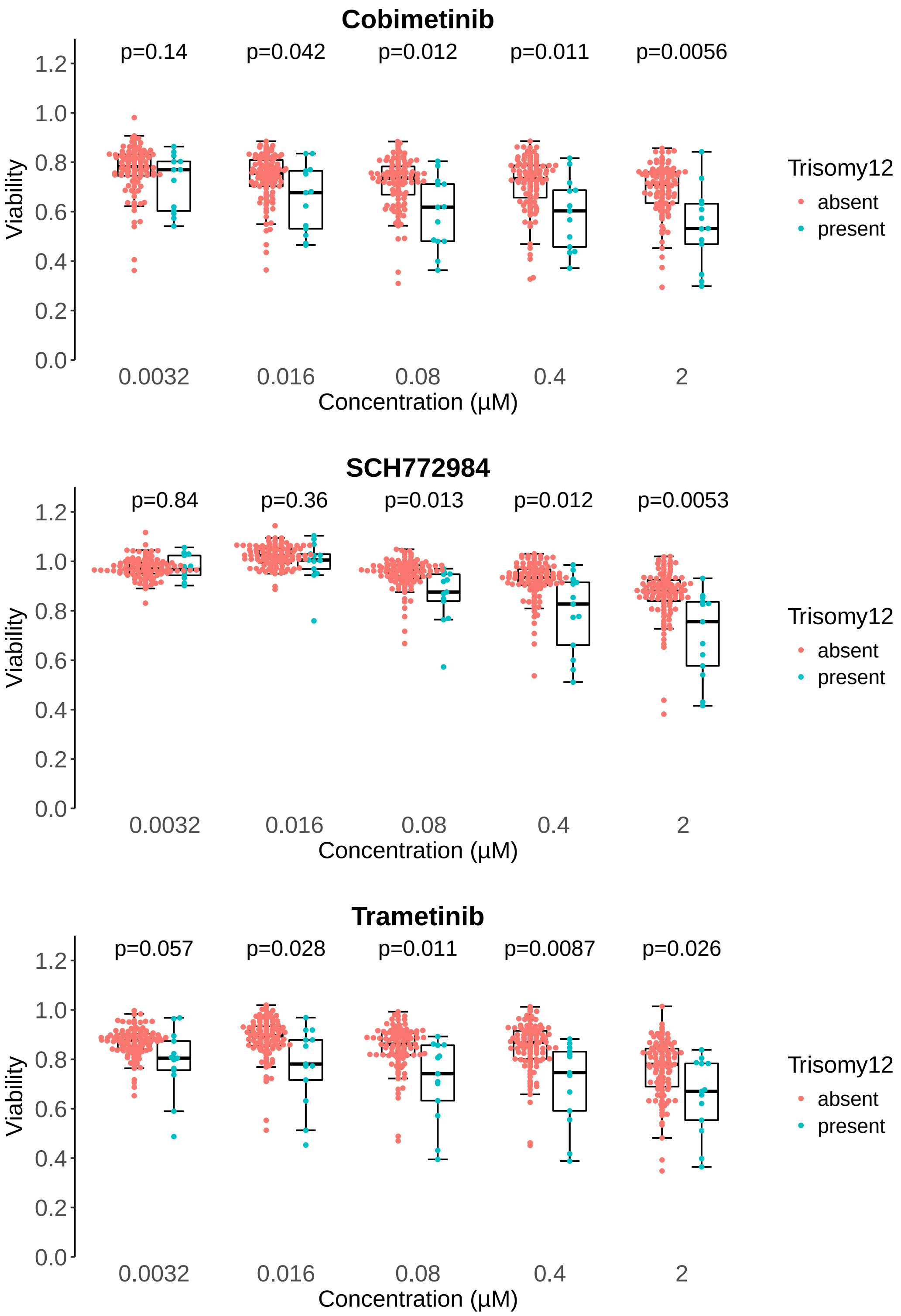

Figure S22: **Drug response associated with trisomy 12.**
The beeswarm plots show drug responses to MEK inhibitors (cobimetinib and trametinib) and ERK inhibitor SCH772984 at five different concentrations stratified by trisomy 12 status ($n = 106$ without trisomy 12 and $n = 13$ with trisomy 12; Student's t-test). ERK and MEK inhibition were more effective in trisomy 12 samples.

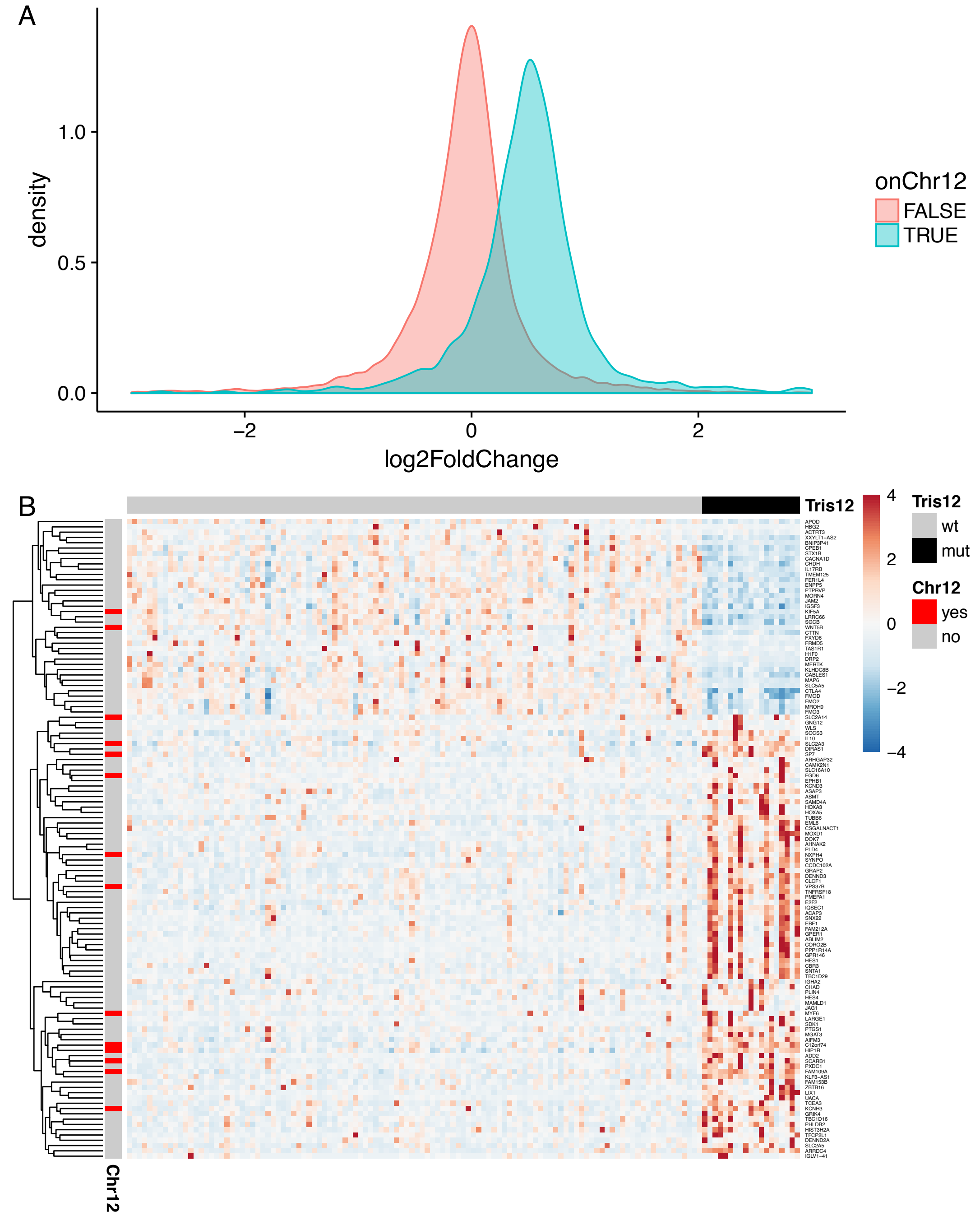

Figure S23: **Gene expression associated with trisomy 12.**
(**A**) Distributions of the $\log_2$-fold change between samples with ($n = 19$) and without ($n = 112$) trisomy 12, shown separately for the genes on chromosome 12 (blue) and on other chromosomes (red). The two distributions are shifted with respect to each other by an amount that is consistent with $\log_2(3/2) \approx 0.58$ and thus with gene dosage effects. (**B**) The heatmap shows row-centered and -scaled gene expression values for differentially expressed genes between patients with and without trisomy 12, based on RNA-Seq data. Red indicates higher expression while blue indicates lower expression. Analysis was performed with *DESeq2* (FDR=10% and absolute $\log_2$-fold change >1.5) [6].

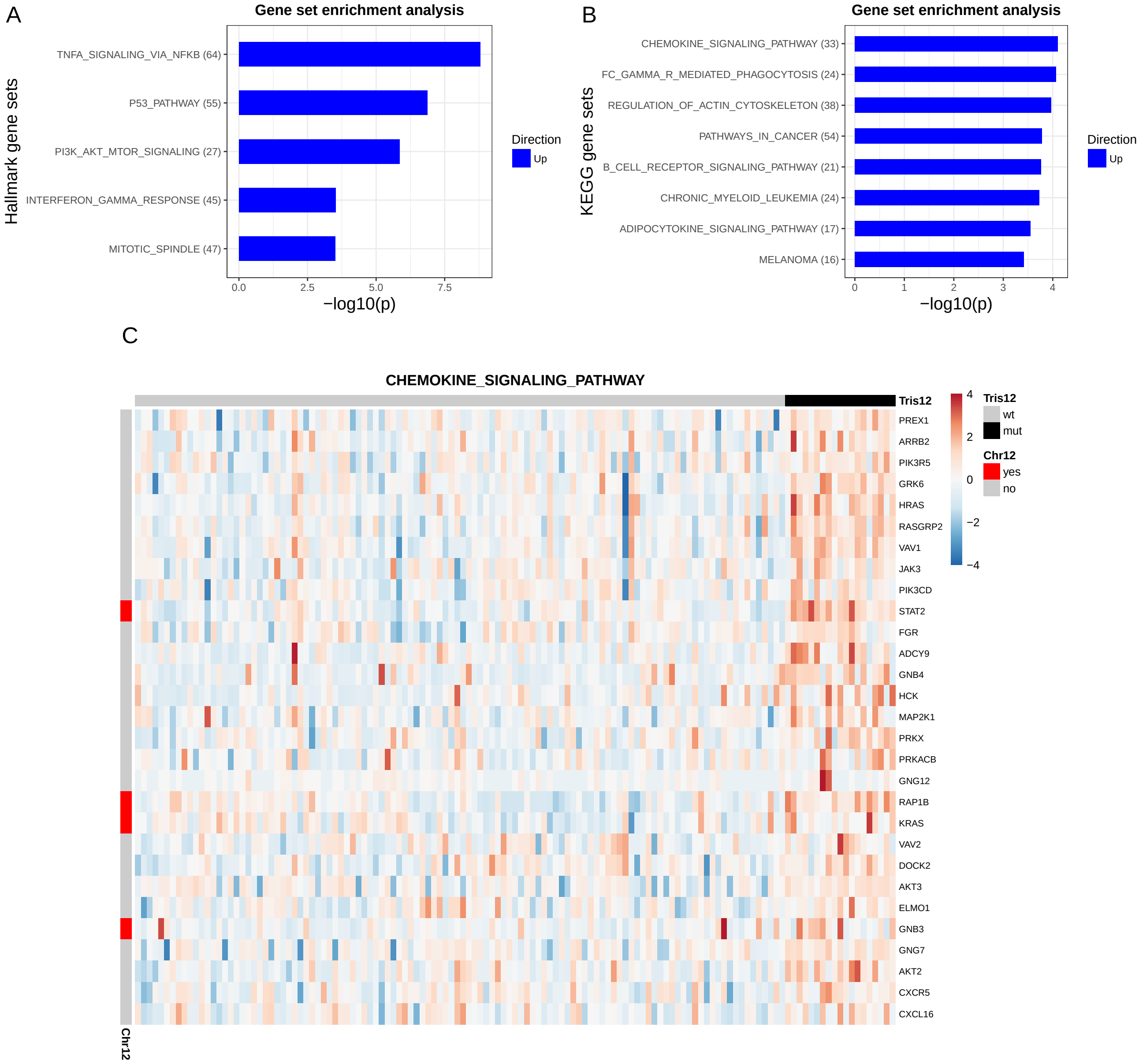

Figure S24: **Gene set enrichment analysis for trisomy 12.**
Differentially expressed genes between patients with (n = 19) and without (n = 112) trisomy 12 (raw p < 0.05) were subjected to a gene enrichment analysis using the Parametric Analysis of Gene Set Enrichment (PAGE) [9]. Panel A shows the result from screening the Hallmark gene sets from MSigDB (http://software.broadinstitute.org/gsea/msigdb), Panel B those for KEGG (http://www.genome.jp/kegg/pathway.html). Shown are the top enriched gene sets, corresponding to a threshold of 1% FDR. (**A**) In line with increased activity of mTOR and PI3K inhibitors in trisomy 12 patients, PI3K-AKT-mTOR associated genes were significantly enriched. (**B**) PAGE identified chemokine signaling, regulation of actin cytoskeleton and BCR signaling to be significantly enriched. (**C**) The heatmap shows the row-centered and -scaled gene expression values (from RNA-seq) for the genes annotated by KEGG to the chemokine signaling pathway and differentially expressed between patients with and without trisomy12 (FDR=10%).

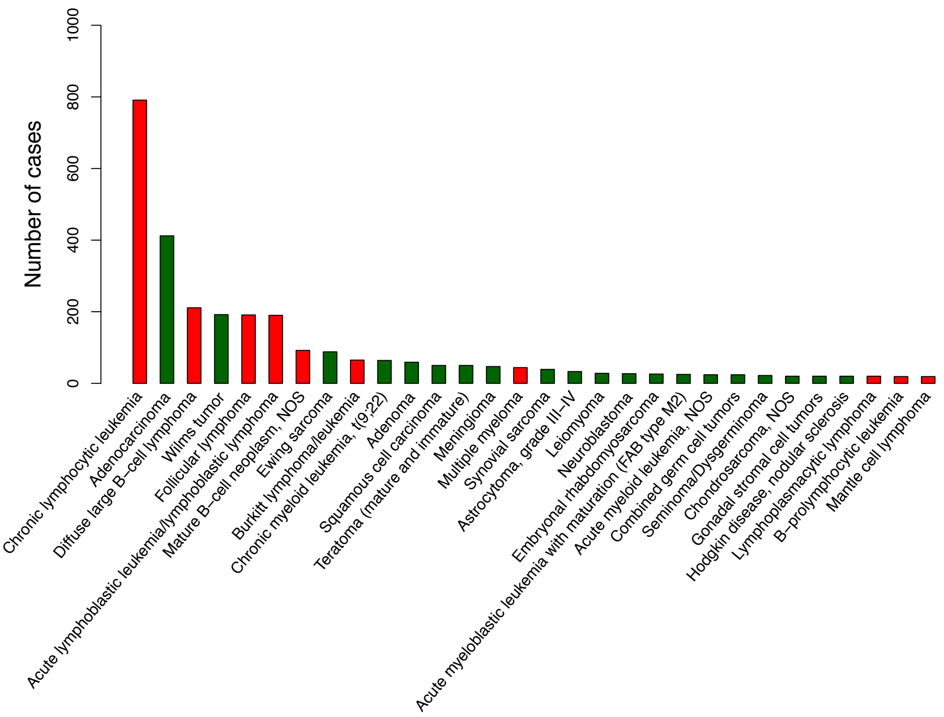

Figure S25: **Prevalence of trisomy 12 across different malignancies.**
The Mitelman Database (Mitelman F, Johansson B and Mertens F (Eds.), `http://cgap.nci.nih.gov/Chromosomes/Mitelman`) was queried for trisomy 12. Abundance of cases with trisomy 12 reported to the Mitelman database are shown. B-cell malignancies are highlighted in red.

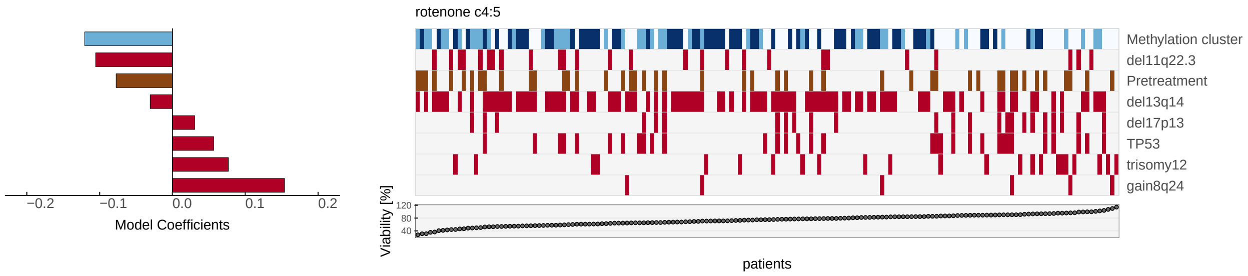

Figure S26: **Lasso model for rotenone.**
The representation and analysis is analogous to Figure 12 of the main text

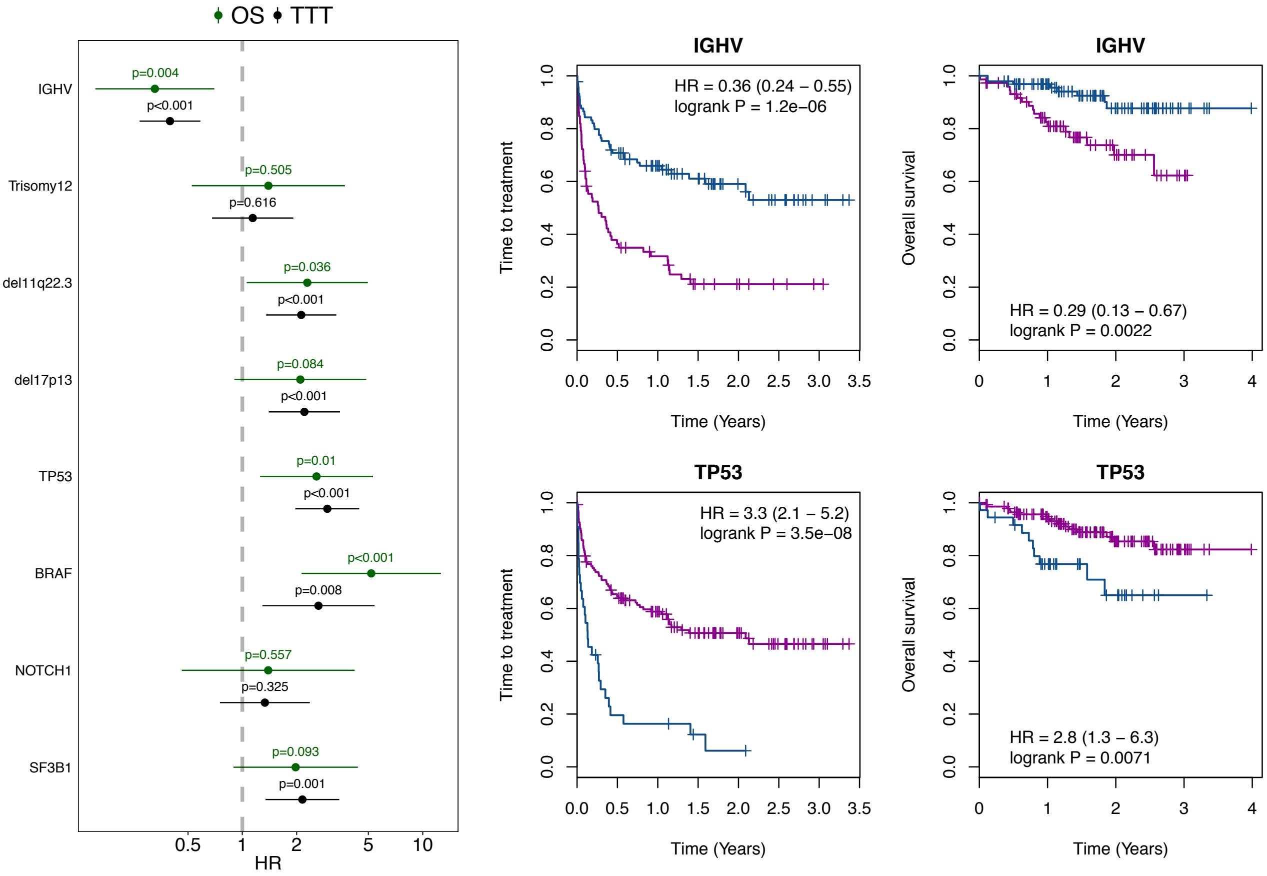

Figure S27: **Impact of genetic factors on survival.**
The **left panel** shows a forest plot for the impact of mutations and CNVs on time to treatment and overall survival. Hazard ratios (HR) were calculated for presence vs. absence of the genetic feature. The **right panels** show exemplary Kaplan-Meier plots for IGHV status and *TP53* mutation status. For the IGHV plots, the blue line represents patients with mutated IGHV locus (≤98% homology) and purple unmutated IGHV locus. For the *TP53* plots, the blue line represents patients with a *TP53* mutation, the purple line patients with no *TP53* mutation. The same Kaplan Meier plot for the impact of TP53 mutations on OS is also shown in Figure 13b.

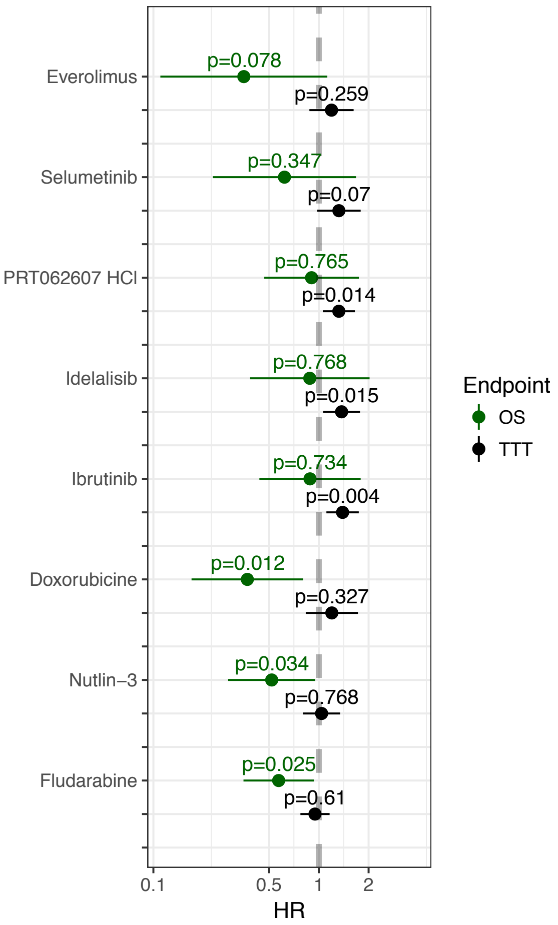

Figure S28: **Impact of drug response on survival in untreated patients.**
The plot is analogous to to figure 13a of the main text, but for this analysis only patients who were untreated were used.

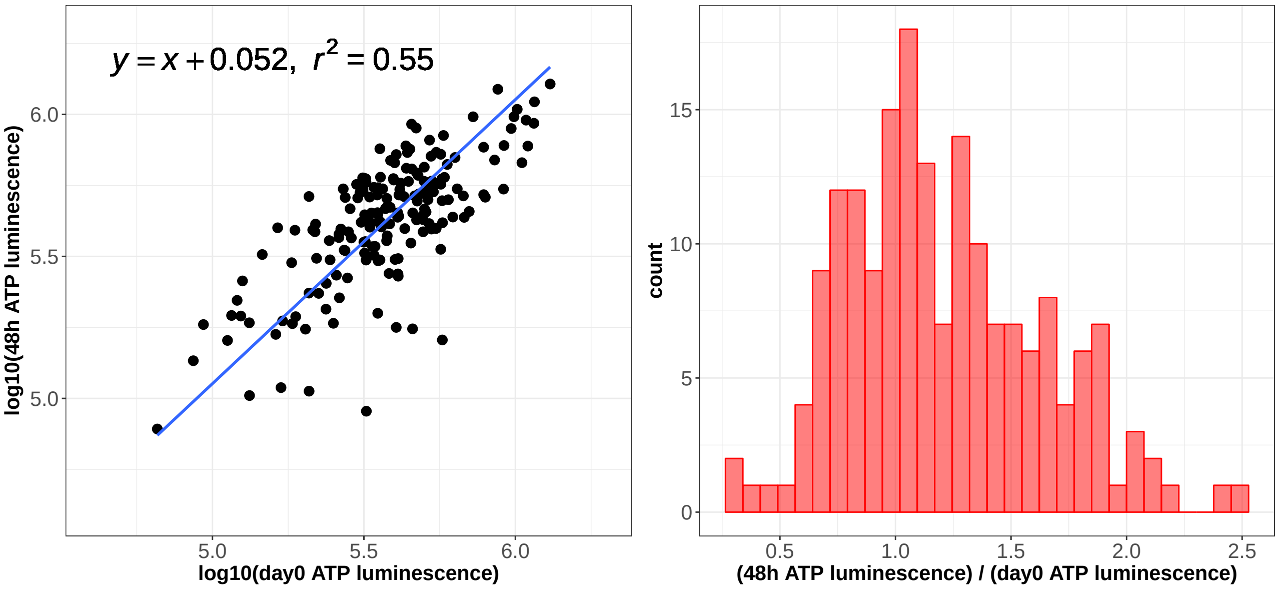

Figure S29: **ATP luminescence of DMSO controls at the beginning and after 48 h of incubation.** The scatterplot on the left shows the ATP luminescence of patient samples without drug treatment (negative controls) at the start of the incubation time (day0) and after 48 hours of culture. The majority of the samples show comparable levels of luminescence at both time points, indicating that viability did not generally decrease in this short-term culture assay. The regression line has an intercept of +0.052, which indicates that on average there is even a moderate increase (13%, since $10^{0.052} \approx 1.13$) of the ATP luminescence during the 48 hours of culture, perhaps due to recovery from freezing stress. The histogram on the right shows ratios of ATP counts between the time points.

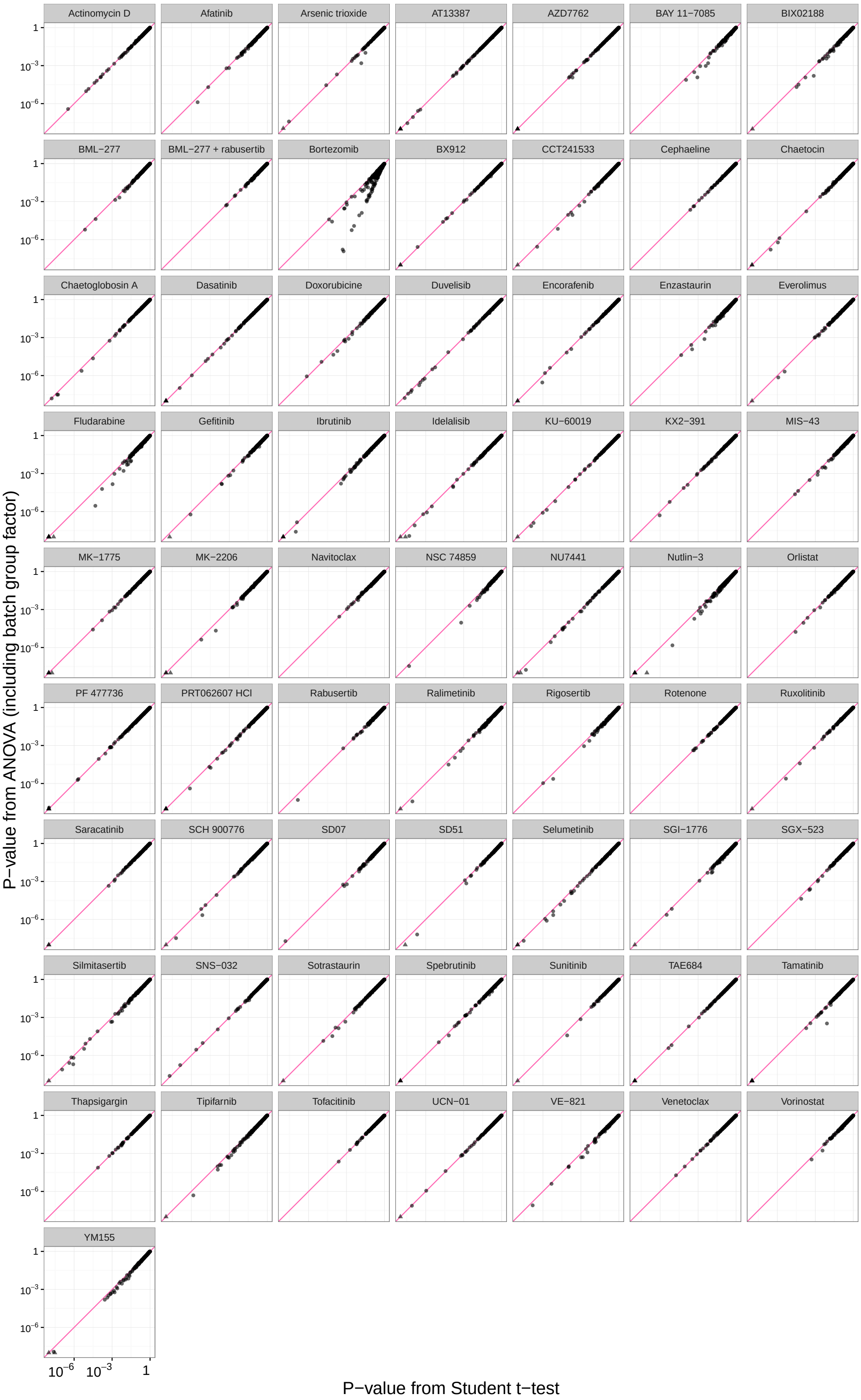

Figure S30: **Assessment of batch effects.**
To assess the potential impact of batch effects associated with different stages of the main screening campaigns, we compared p-values for associations between drug responses and genetic features computed in two different ways: two-way ANOVA with screening time point batches as a blocking factor (y-axis) and Student's t-test, which does not account for batch groups (x-axis). p-values below 10^{-8} in at least one of the two analyses are shown with triangular symbols. The results show good agreement for all drugs except for bortezomib.

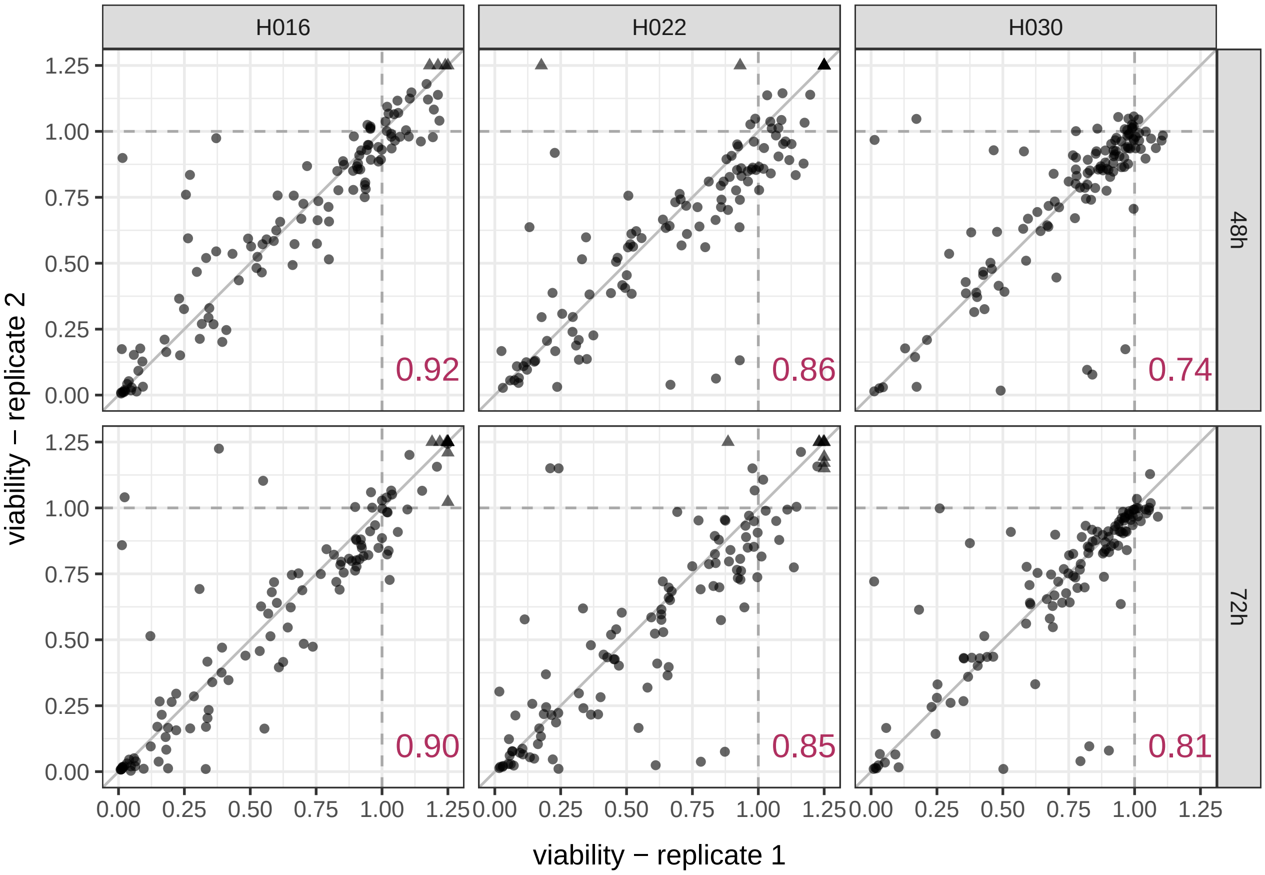

Figure S31: **Reproducibility of drug response measurements.**
The scatter plots show comparisons of drug response measurements in three patient samples that were repeatedly assayed at two different time points. Repeated measurements were performed for 48 and 72 h incubation time. Pearson correlation coefficients for measurements are shown within each plot. Triangles indicate data points outside the plotting range.

6 Supplementary tables

Table S1: **Drugs.** Drugs included in the drug screen.

Drug ID	Compound	Main targets	Distributor
D_001	navitoclax	BCL2, BCL-XL, BCL-W	Selleck Chemicals
D_002	ibrutinib	BTK	Selleck Chemicals
D_003	idelalisib	PI3K delta	Selleck Chemicals
D_004	SNS-032	CDK2/7/9	Selleck Chemicals
D_006	fludarabine	Purine analogue	Selleck Chemicals
D_007	vorinostat	HDAC I/IIa/IIb/IV	Selleck Chemicals
D_008	bortezomib	Proteasome	Selleck Chemicals
D_010	nutlin-3	MDM2	Selleck Chemicals
D_011	enzastaurin	PKC	Selleck Chemicals
D_012	selumetinib	MEK1/2	Selleck Chemicals
D_013	afatinib	EGFR, ERBB2	Selleck Chemicals
D_015	MK-1775	WEE1	Selleck Chemicals
D_017	AT13387	HSP90	Selleck Chemicals
D_020	AZD7762	CHK1/2	Selleck Chemicals
D_021	rigosertib	PLK	Selleck Chemicals
D_023	ralimetinib	p38 MAPK	Selleck Chemicals
D_024	SGI-1776	PIM	Selleck Chemicals
D_025	NSC 74859	STAT	Selleck Chemicals
D_029	TAE684	ALK	Selleck Chemicals
D_030	MK-2206	AKT1/2 (PKB)	Selleck Chemicals
D_032	NU7441	DNAPK	Selleck Chemicals
D_033	tipifarnib	Farnesyltransferase (FNTA)	Selleck Chemicals
D_034	chaetocin	Lysine-specific histone methyltransferase	Sigma-Aldrich
D_035	saracatinib	SRC, ABL1	Selleck Chemicals
D_036	tamatinib	SYK	Selleck Chemicals
D_039	thapsigargin	Sarco/endoplasmic reticulum Ca2+ ATPase (SERCA)	Enzo Life Sciences
D_040	YM155	Survivin	Selleck Chemicals
D_041	BAY 11-7085	NFkB	Enzo Life Sciences
D_043	SGX-523	MET	Selleck Chemicals
D_045	KU-60019	ATM	Selleck Chemicals
D_048	orlistat	LPL	Cayman Chemicals
D_049	chaetoglobosin A	Actin	Enzo Life Sciences
D_050	dasatinib	ABL1, KIT, LYN, PDGFRA, PDGFRB, SRC	Selleck Chemicals
D_053	sunitinib	VEGFR, PDGFRA/B, FLT3, KIT	Selleck Chemicals
D_054	gefitinib	EGFR	Selleck Chemicals
D_056	actinomycin D	RNA synthesis	Sigma-Aldrich
D_060	cephaeline	40S ribosomal subunit	Sigma-Aldrich
D_063	everolimus	mTOR	Selleck Chemicals
D_066	arsenic trioxide		Sigma-Aldrich
D_067	rotenone	Electron transport chain in mitochondria	Sigma-Aldrich
D_071	KX2-391	SRC	Selleck Chemicals
D_074	VE-821	ATR	Selleck Chemicals
D_075	rabusertib	CHK1	Selleck Chemicals
D_077	SCH 900776	CHK1, CDK2	Selleck Chemicals
D_078	PF 477736	CHK1, CHK2	Selleck Chemicals
D_079	spebrutinib	BTK	Celgene
D_081	venetoclax	BCL2	Selleck Chemicals
D_082	duvelisib	PI3K gamma, PI3K delta	Selleck Chemicals
D_083	encorafenib	BRAF	Novartis
D_084	ruxolitinib	JAK1/2/3	Selleck Chemicals

D_127	SD07	ROS	Academic cooperation
D_141	SD51	ROS	Academic cooperation
D_149	MIS-43	ROS	Academic cooperation
D_159	doxorubicine	DNA intercalation, Topoisomerase II	Sigma-Aldrich
D_162	BML-277	CHK2	Sigma-Aldrich
D_163	CCT241533	CHK2	MedChem Express
D_164	BX912	PDK1	Selleck Chemicals
D_165	silmitasertib	CK2	Selleck Chemicals
D_166	PRT062607 HCl	SYK	Selleck Chemicals
D_167	UCN-01	PKC, MK2, CHK1	Sigma-Aldrich
D_168	sotrastaurin	PKC	Selleck Chemicals
D_169	tofacitinib	JAK3	Selleck Chemicals
D_172	BIX02188	MEK5	Selleck Chemicals
D_CHK	BML-277 + rabusertib		

Table S2: **Drug concentrations.** Drug concentrations used in the drug screen.

Drug ID	c1 [μM]	c2 [μM]	c3 [μM]	c4 [μM]	c5 [μM]
D_001	1.000	0.250	0.063	0.016	0.004
D_002	40.000	10.000	2.500	0.625	0.156
D_003	40.000	10.000	2.500	0.625	0.156
D_004	4.000	1.000	0.250	0.063	0.016
D_006	40.000	10.000	2.500	0.625	0.156
D_007	20.000	5.000	1.250	0.313	0.078
D_008	20.000	5.000	1.250	0.313	0.078
D_010	40.000	10.000	2.500	0.625	0.156
D_011	40.000	10.000	2.500	0.625	0.156
D_012	40.000	10.000	2.500	0.625	0.156
D_013	15.000	5.000	1.667	0.556	0.185
D_015	40.000	10.000	2.500	0.625	0.156
D_017	10.000	2.500	0.625	0.156	0.039
D_020	40.000	10.000	2.500	0.625	0.156
D_021	40.000	10.000	2.500	0.625	0.156
D_023	40.000	10.000	2.500	0.625	0.156
D_024	40.000	10.000	2.500	0.625	0.156
D_025	40.000	10.000	2.500	0.625	0.156
D_029	40.000	10.000	2.500	0.625	0.156
D_030	40.000	10.000	2.500	0.625	0.156
D_032	40.000	10.000	2.500	0.625	0.156
D_033	40.000	10.000	2.500	0.625	0.156
D_034	2.000	0.500	0.125	0.031	0.008
D_035	40.000	10.000	2.500	0.625	0.156
D_036	40.000	10.000	2.500	0.625	0.156
D_039	20.000	5.000	1.250	0.313	0.078
D_040	2.000	0.500	0.125	0.031	0.008
D_041	40.000	10.000	2.500	0.625	0.156
D_043	40.000	10.000	2.500	0.625	0.156
D_045	40.000	10.000	2.500	0.625	0.156
D_048	40.000	10.000	2.500	0.625	0.156
D_049	20.000	10.000	5.000	2.500	1.250
D_050	40.000	10.000	2.500	0.625	0.156
D_053	40.000	10.000	2.500	0.625	0.156
D_054	40.000	10.000	2.500	0.625	0.156
D_056	0.300	0.100	0.033	0.011	0.004
D_060	4.000	1.000	0.250	0.063	0.016
D_063	40.000	10.000	2.500	0.625	0.156
D_066	8.000	4.000	2.000	1.000	0.500
D_067	40.000	10.000	2.500	0.625	0.156
D_071	40.000	10.000	2.500	0.625	0.156
D_074	40.000	10.000	2.500	0.625	0.156
D_075	40.000	10.000	2.500	0.625	0.156
D_077	40.000	10.000	2.500	0.625	0.156
D_078	40.000	10.000	2.500	0.625	0.156
D_079	40.000	10.000	2.500	0.625	0.156
D_081	1.000	0.250	0.063	0.016	0.004
D_082	40.000	10.000	2.500	0.625	0.156
D_083	40.000	10.000	2.500	0.625	0.156
D_084	40.000	10.000	2.500	0.625	0.156
D_127	30.000	10.000	3.333	1.111	0.370
D_141	30.000	10.000	3.333	1.111	0.370
D_149	30.000	10.000	3.333	1.111	0.370
D_159	4.000	1.000	0.250	0.063	0.016
D_162	40.000	10.000	2.500	0.625	0.156
D_163	40.000	10.000	2.500	0.625	0.156
D_164	40.000	10.000	2.500	0.625	0.156
D_165	40.000	10.000	2.500	0.625	0.156

D_166	40.000	10.000	2.500	0.625	0.156
D_167	10.000	2.500	0.625	0.156	0.039
D_168	40.000	10.000	2.500	0.625	0.156
D_169	40.000	10.000	2.500	0.625	0.156
D_172	40.000	10.000	2.500	0.625	0.156
D_CHK	10.000	2.500	0.625	0.156	0.039

Table S3: **Patient characteristics.** Characteristics of samples used in the drug screen (n.d. - no data available).

No	Patient ID	Diagnosis	Age	Sex	IGHV status	Treated	Alive
1	H001	hMNC	n.d.	n.d.	n.d.	n.d.	n.d.
2	H002	hMNC	n.d.	n.d.	n.d.	n.d.	n.d.
3	H003	hMNC	n.d.	n.d.	n.d.	n.d.	n.d.
4	H005	CLL	75	male	mutated	yes	yes
5	H009	B-PLL	57	male	unmutated	yes	yes
6	H010	CLL	73	female	unmutated	no	yes
7	H011	CLL	73	female	mutated	no	yes
8	H012	CLL	62	female	unmutated	yes	no
9	H013	CLL	77	male	unmutated	yes	no
10	H014	CLL	86	female	unmutated	yes	no
11	H015	CLL	62	female	unmutated	no	yes
12	H016	CLL	55	male	mutated	no	yes
13	H017	CLL	56	male	unmutated	no	no
14	H019	CLL	70	female	unmutated	yes	no
15	H020	CLL	64	male	mutated	no	yes
16	H021	CLL	50	male	mutated	no	yes
17	H023	CLL	71	female	unmutated	yes	no
18	H025	T-PLL	73	male	n.d.	yes	no
19	H026	LPL	59	male	mutated	no	yes
20	H027	CLL	58	male	unmutated	no	yes
21	H028	CLL	73	female	mutated	no	no
22	H029	CLL	75	female	mutated	yes	yes
23	H030	CLL	53	male	unmutated	no	yes
24	H031	CLL	62	female	mutated	no	yes
25	H032	CLL	67	male	unmutated	yes	no
26	H033	CLL	63	female	mutated	no	yes
27	H035	CLL	79	female	mutated	yes	yes
28	H036	CLL	75	female	mutated	no	yes
29	H037	CLL	71	male	mutated	no	yes
30	H038	CLL	74	male	mutated	no	yes
31	H039	CLL	55	female	mutated	no	yes
32	H040	CLL	84	female	mutated	no	no
33	H041	CLL	76	male	mutated	no	yes
34	H042	CLL	72	female	unmutated	yes	no
35	H043	CLL	44	female	unmutated	yes	yes
36	H044	CLL	61	male	unmutated	yes	yes
37	H045	CLL	91	male	unmutated	yes	no
38	H046	CLL	88	male	mutated	no	yes
39	H047	CLL	69	male	unmutated	yes	no
40	H048	CLL	65	female	unmutated	yes	yes
41	H049	CLL	58	male	mutated	no	yes
42	H050	CLL	63	female	mutated	no	yes
43	H051	CLL	79	female	unmutated	yes	no
44	H053	CLL	83	female	mutated	no	yes
45	H054	CLL	50	female	mutated	no	yes
46	H055	CLL	65	male	mutated	no	yes
47	H056	CLL	83	male	mutated	no	yes
48	H057	CLL	67	male	mutated	no	yes
49	H058	CLL	75	female	mutated	no	no
50	H059	CLL	55	male	mutated	no	yes
51	H060	CLL	75	male	unmutated	no	yes
52	H062	CLL	53	male	mutated	no	yes
53	H063	CLL	49	female	mutated	no	yes
54	H064	CLL	71	male	n.d.	yes	yes
55	H065	CLL	77	female	unmutated	yes	no
56	H066	CLL	47	male	unmutated	yes	no
57	H067	CLL	77	female	mutated	no	yes
58	H069	CLL	77	female	unmutated	yes	no
59	H070	CLL	71	male	n.d.	no	yes
60	H071	FL	60	male	mutated	no	yes
61	H072	CLL	58	male	unmutated	no	yes
62	H073	CLL	65	male	mutated	yes	yes

63	H076	MCL	67	male	mutated	no	yes
64	H077	CLL	70	female	unmutated	no	yes
65	H078	CLL	68	male	unmutated	yes	yes
66	H079	CLL	48	male	unmutated	no	yes
67	H080	CLL	82	male	unmutated	yes	yes
68	H081	CLL	64	female	mutated	no	yes
69	H082	CLL	82	male	mutated	no	yes
70	H083	CLL	69	male	n.d.	no	yes
71	H084	CLL	88	male	mutated	no	yes
72	H086	T-PLL	64	male	n.d.	no	yes
73	H088	CLL	60	female	mutated	no	yes
74	H089	CLL	55	female	mutated	no	yes
75	H090	CLL	70	female	mutated	yes	yes
76	H092	MZL	82	male	mutated	no	yes
77	H093	CLL	76	female	unmutated	no	yes
78	H094	CLL	46	male	mutated	no	yes
79	H095	CLL	53	female	unmutated	no	yes
80	H096	CLL	62	female	n.d.	no	yes
81	H098	MCL	79	male	unmutated	no	no
82	H099	CLL	54	female	mutated	no	yes
83	H100	CLL	74	male	mutated	no	yes
84	H101	CLL	73	female	mutated	no	yes
85	H102	CLL	78	female	unmutated	no	yes
86	H103	CLL	71	male	mutated	no	yes
87	H104	CLL	79	male	unmutated	no	yes
88	H105	CLL	49	male	mutated	no	yes
89	H106	CLL	71	male	mutated	no	yes
90	H107	CLL	43	male	unmutated	no	yes
91	H108	CLL	57	male	mutated	no	yes
92	H109	CLL	85	male	unmutated	no	yes
93	H110	CLL	66	male	mutated	no	yes
94	H111	CLL	55	male	unmutated	yes	no
95	H113	CLL	70	male	mutated	no	yes
96	H115	CLL	72	male	mutated	no	no
97	H117	CLL	51	female	unmutated	yes	yes
98	H118	CLL	49	male	mutated	yes	yes
99	H120	MZL	74	female	mutated	no	yes
100	H122	LPL	53	male	mutated	no	yes
101	H126	T-PLL	68	male	n.d.	no	yes
102	H127	T-PLL	69	female	n.d.	no	no
103	H128	T-PLL	78	female	n.d.	no	yes
104	H133	CLL	69	male	n.d.	no	yes
105	H134	Sezary	67	male	n.d.	yes	yes
106	H135	CLL	76	female	mutated	yes	no
107	H136	CLL	66	male	unmutated	yes	yes
108	H137	CLL	53	male	mutated	no	yes
109	H140	HCL	55	female	n.d.	no	yes
110	H141	MCL	46	female	n.d.	yes	no
111	H142	MCL	67	male	n.d.	no	yes
112	H143	HCL-V	n.d.	male	n.d.	n.d.	n.d.
113	H144	MCL	67	male	n.d.	yes	no
114	H145	HCL	45	male	n.d.	no	yes
115	H146	MCL	n.d.	male	n.d.	n.d.	no
116	H147	MCL	59	male	n.d.	no	yes
117	H148	CLL	34	female	unmutated	yes	no
118	H149	T-PLL	83	male	n.d.	no	no
119	H150	T-PLL	75	female	n.d.	no	yes
120	H151	T-PLL	63	female	n.d.	no	no
121	H152	T-PLL	42	female	n.d.	no	no
122	H153	T-PLL	52	female	n.d.	yes	no
123	H154	T-PLL	65	female	n.d.	no	no
124	H155	T-PLL	74	female	n.d.	yes	no
125	H156	B-PLL	61	female	n.d.	no	no
126	H157	T-PLL	70	female	n.d.	no	no
127	H158	MZL	60	male	n.d.	yes	yes
128	H159	LPL	58	female	n.d.	no	yes

129	H160	LPL	62	male	n.d.	yes	yes
130	H161	T-PLL	83	male	n.d.	no	yes
131	H162	MZL	50	female	n.d.	no	yes
132	H163	CLL	65	male	mutated	no	yes
133	H164	CLL	73	female	unmutated	no	yes
134	H165	CLL	58	female	unmutated	no	yes
135	H166	CLL	63	female	unmutated	no	yes
136	H167	CLL	64	female	unmutated	no	yes
137	H168	CLL	58	male	n.d.	yes	yes
138	H169	CLL	42	female	mutated	no	yes
139	H170	CLL	75	female	mutated	yes	yes
140	H171	CLL	73	male	unmutated	yes	yes
141	H172	T-PLL	45	male	n.d.	no	yes
142	H173	CLL	74	female	mutated	yes	yes
143	H174	CLL	64	female	unmutated	yes	yes
144	H175	CLL	62	male	unmutated	yes	yes
145	H176	CLL	70	male	mutated	no	yes
146	H177	CLL	70	male	unmutated	no	yes
147	H178	CLL	71	male	unmutated	yes	yes
148	H179	CLL	61	male	mutated	no	yes
149	H180	CLL	86	male	unmutated	no	yes
150	H181	CLL	76	female	mutated	no	yes
151	H182	CLL	72	female	mutated	no	yes
152	H183	CLL	70	male	unmutated	no	yes
153	H184	CLL	75	male	mutated	no	no
154	H185	CLL	87	female	mutated	no	no
155	H186	CLL	73	female	mutated	no	yes
156	H187	CLL	60	male	unmutated	no	yes
157	H188	T-PLL	71	male	n.d.	no	no
158	H189	T-PLL	71	male	n.d.	yes	yes
159	H190	MCL	66	male	n.d.	no	yes
160	H191	CLL	39	male	n.d.	yes	yes
161	H192	CLL	72	female	mutated	no	yes
162	H193	CLL	81	male	mutated	no	yes
163	H194	CLL	76	male	mutated	no	yes
164	H195	T-PLL	76	male	n.d.	no	yes
165	H196	CLL	85	male	mutated	no	yes
166	H197	CLL	74	female	mutated	yes	yes
167	H198	CLL	58	female	mutated	no	yes
168	H199	CLL	83	male	mutated	no	yes
169	H200	CLL	83	female	unmutated	yes	yes
170	H201	CLL	72	female	n.d.	no	yes
171	H202	CLL	80	male	mutated	n.d.	yes
172	H203	CLL	83	female	mutated	no	yes
173	H204	CLL	66	female	n.d.	no	yes
174	H205	CLL	66	female	unmutated	no	yes
175	H206	CLL	69	male	mutated	no	yes
176	H207	CLL	65	male	mutated	no	yes
177	H208	CLL	73	male	mutated	no	yes
178	H209	CLL	72	female	mutated	yes	yes
179	H210	CLL	73	female	mutated	no	yes
180	H211	CLL	51	male	unmutated	no	yes
181	H212	CLL	74	male	mutated	no	yes
182	H213	CLL	63	male	mutated	no	yes
183	H214	CLL	65	male	unmutated	no	yes
184	H215	CLL	47	male	unmutated	no	yes
185	H216	CLL	48	female	mutated	no	yes
186	H217	CLL	65	male	mutated	no	yes
187	H218	CLL	50	male	n.d.	yes	yes
188	H219	CLL	74	female	mutated	no	yes
189	H220	CLL	75	female	mutated	no	yes
190	H221	CLL	55	male	mutated	no	yes
191	H222	CLL	56	male	mutated	no	yes
192	H223	CLL	47	female	mutated	no	yes
193	H224	CLL	47	male	unmutated	no	yes
194	H225	CLL	47	female	mutated	no	yes

195	H226	HCL	64	male	n.d.	no	yes
196	H227	MCL	64	male	n.d.	no	no
197	H228	CLL	65	male	unmutated	no	yes
198	H229	CLL	75	female	mutated	yes	no
199	H230	CLL	71	male	unmutated	yes	no
200	H231	CLL	47	male	unmutated	no	yes
201	H232	T-PLL	62	male	n.d.	no	yes
202	H233	CLL	55	male	unmutated	no	yes
203	H234	CLL	68	male	unmutated	no	yes
204	H235	CLL	73	male	mutated	no	yes
205	H236	CLL	67	male	mutated	no	yes
206	H237	CLL	73	female	mutated	no	yes
207	H238	CLL	75	male	unmutated	no	no
208	H239	CLL	70	female	unmutated	no	yes
209	H240	CLL	83	male	mutated	no	yes
210	H241	Sezary	59	male	n.d.	no	yes
211	H242	CLL	49	male	unmutated	no	yes
212	H243	CLL	80	male	unmutated	no	yes
213	H244	B-PLL	80	male	n.d.	n.d.	yes
214	H245	PTCL-NOS	80	male	n.d.	yes	yes
215	H246	CLL	75	male	unmutated	no	yes
216	H247	CLL	47	female	mutated	no	yes
217	H248	CLL	63	female	mutated	no	yes
218	H249	CLL	83	male	unmutated	no	yes
219	H250	CLL	52	male	unmutated	no	yes
220	H251	HCL-V	73	male	n.d.	no	yes
221	H252	CLL	70	male	unmutated	no	yes
222	H253	T-PLL	57	female	n.d.	no	yes
223	H254	CLL	75	male	mutated	no	yes
224	H255	CLL	67	male	unmutated	yes	yes
225	H256	CLL	63	female	n.d.	yes	yes
226	H257	CLL	66	female	unmutated	no	yes
227	H258	CLL	65	male	mutated	no	yes
228	H259	CLL	60	male	unmutated	yes	yes
229	H260	CLL	63	male	unmutated	yes	yes
230	H261	T-PLL	49	female	n.d.	no	yes
231	H262	T-PLL	86	male	n.d.	no	n.d.
232	H263	T-PLL	77	male	n.d.	n.d.	n.d.
233	H264	CLL	77	male	mutated	yes	yes
234	H265	CLL	59	male	unmutated	yes	yes
235	H266	CLL	74	male	mutated	yes	yes
236	H267	T-PLL	68	male	n.d.	no	no
237	H268	CLL	83	male	n.d.	no	yes
238	H269	MCL	46	female	n.d.	no	no
239	H270	CLL	67	female	mutated	no	yes
240	H271	CLL	65	male	mutated	no	yes
241	H272	CLL	56	male	unmutated	yes	yes
242	H273	MZL	77	male	n.d.	yes	yes
243	H274	AML	61	female	n.d.	yes	yes
244	H275	AML	77	female	n.d.	no	no
245	H276	AML	62	male	n.d.	no	yes
246	H277	AML	83	male	n.d.	no	no
247	H278	AML	75	male	n.d.	yes	yes
248	H279	T-PLL	58	male	n.d.	no	yes
249	H280	MZL	63	male	n.d.	no	yes

Table S4: **Sequencing quality.** (IS: paired-end insert size)

Patient ID	Sample type	On target [%]	Coverage	Total number of counts	Properly paired counts [%]	Singletons [%]	Duplicates [%]	IS sd	IS median	IS mean
H005	control	70.87	101.87	114477796	99.16	0.09	9.94	58.76	183.00	195.98
H005	tumor	69.85	100.73	113683108	99.31	0.08	9.34	67.14	195.00	209.35
H006	control	71.33	92.22	102245696	98.80	0.12	9.32	62.16	183.00	196.19
H006	tumor	71.31	85.82	95976636	98.84	0.10	8.56	62.78	184.00	197.75
H008	control	71.04	116.08	134779454	98.79	0.13	12.43	57.55	178.00	190.42
H008	tumor	71.00	120.55	137208440	98.75	0.13	11.25	60.47	180.00	193.24
H009	control	71.89	58.00	60520750	98.70	0.09	4.84	65.61	185.00	199.4
H009	tumor	70.35	100.65	108254616	98.68	0.11	5.44	63.84	184.00	197.61
H010	control	69.71	100.81	119497348	98.28	0.35	9.82	57.92	181.00	193.18
H010	tumor	69.71	122.99	137540714	99.12	0.14	7.49	64.91	190.00	204.34
H011	control	70.50	96.26	108350558	99.38	0.08	9.70	63.79	192.00	205.63
H011	tumor	70.79	119.71	133363064	99.11	0.09	9.38	61.04	186.00	198.54
H012	control	72.45	103.55	136812374	97.92	0.30	23.67	60.60	209.00	218.72
H012	tumor	69.20	146.78	169012662	99.34	0.10	9.78	62.45	197.00	210.04
H013	control	71.72	112.70	141532794	99.32	0.11	19.82	59.57	210.00	219.76
H013	tumor	71.85	125.19	157586056	99.33	0.10	20.16	61.36	214.00	223.9
H014	control	69.97	86.59	99911814	98.97	0.12	8.89	62.56	186.00	199.38
H014	tumor	70.46	101.82	118072082	98.77	0.16	9.48	59.61	183.00	195.34
H015	control	69.48	80.74	95228312	98.80	0.16	10.12	59.46	182.00	194.13
H015	tumor	70.90	96.18	113157896	98.84	0.15	11.57	60.52	184.00	196.86
H016	control	70.22	95.80	106555668	99.18	0.09	8.26	62.13	186.00	199.41
H016	tumor	72.80	141.72	153687492	99.07	0.23	8.76	63.37	197.00	210.06
H017	control	70.84	97.14	113931382	99.00	0.13	11.43	62.22	185.00	198.5
H017	tumor	71.53	99.70	116371806	98.83	0.16	11.52	60.44	182.00	194.59
H018	control	69.98	149.98	183593582	98.89	0.13	15.60	63.09	184.00	197.78
H018	tumor	70.52	118.58	135685410	98.85	0.12	11.09	63.42	185.00	199
H019	control	70.15	96.87	113565082	98.79	0.13	10.44	62.82	186.00	198.95
H019	tumor	68.91	94.43	105641130	99.15	0.09	7.24	65.19	191.00	205.23
H020	control	69.84	93.73	106990310	99.37	0.08	10.02	62.47	191.00	204.17
H020	tumor	70.32	114.38	130986324	99.16	0.09	11.25	66.25	192.00	206.5
H021	control	71.77	120.61	153754612	99.11	0.14	20.79	61.52	211.00	221.84
H021	tumor	71.66	110.70	150228640	99.17	0.14	25.52	61.58	211.00	221.81
H022	control	72.04	124.51	156901158	99.26	0.12	20.55	61.50	213.00	223.23
H022	tumor	70.55	123.14	150974090	99.28	0.11	16.47	62.30	212.00	222.5
H023	control	70.63	108.46	120513460	99.30	0.08	8.78	59.12	187.00	198.79
H023	tumor	69.78	106.38	119232810	99.14	0.09	8.59	63.48	189.00	202.36
H024	control	69.31	103.93	116213034	99.32	0.09	7.53	66.04	195.00	208.64
H024	tumor	72.98	141.43	152657364	98.86	0.24	8.41	64.23	195.00	208.14
H026	control	70.21	81.89	93474556	98.72	0.15	10.31	61.91	182.00	195.84
H026	tumor	70.06	103.44	118141886	99.02	0.10	9.07	62.37	186.00	198.93
H027	control	70.97	130.22	143390020	99.22	0.12	7.94	60.08	192.00	204.72
H027	tumor	71.46	132.53	151252616	99.34	0.11	11.90	62.91	197.00	210.45
H028	control	69.59	149.20	171792170	99.21	0.09	10.59	64.07	190.00	203.46
H028	tumor	72.36	132.09	141908682	99.11	0.22	7.43	63.13	199.00	212.23
H029	control	70.69	98.43	110639068	99.16	0.10	9.80	62.45	188.00	201.12
H029	tumor	73.40	140.89	152283464	99.05	0.23	9.29	65.79	202.00	215.39
H030	control	70.62	111.80	139664122	99.15	0.12	18.10	61.86	213.00	223.75
H030	tumor	70.20	115.83	151721430	99.34	0.11	21.50	62.55	214.00	224.05
H031	control	70.57	83.18	92086494	99.28	0.08	8.36	66.08	193.00	207.45
H031	tumor	72.71	115.50	126724824	99.02	0.24	9.65	65.79	201.00	214.56
H033	control	69.58	121.11	136357860	99.37	0.08	8.48	61.71	189.00	202.48
H033	tumor	71.14	132.89	150202808	99.20	0.08	11.21	61.99	189.00	201.73
H035	control	71.74	107.57	124637084	98.78	0.13	13.19	57.85	178.00	190.13
H035	tumor	71.47	114.69	134956232	98.73	0.13	14.69	62.21	182.00	195.54
H036	control	70.44	92.28	104265752	99.39	0.07	10.07	64.43	193.00	206.93
H036	tumor	70.23	123.14	137064690	99.08	0.10	8.56	65.06	190.00	203.76
H037	control	70.62	69.23	73580076	98.73	0.09	4.83	63.87	184.00	197.38
H037	tumor	70.74	95.10	102603564	98.69	0.08	6.54	69.31	191.00	206.28
H038	control	71.45	69.61	73984798	98.73	0.08	5.97	70.32	190.00	205.4
H038	tumor	69.95	88.68	96480080	98.47	0.10	6.05	65.60	185.00	198.73
H039	control	71.00	96.25	107941940	99.33	0.08	10.06	60.55	187.00	200.05
H039	tumor	70.93	123.11	136533708	99.27	0.08	9.24	63.64	190.00	203.98
H040	control	70.48	84.54	94484164	99.26	0.09	9.03	60.07	186.00	198.78
H040	tumor	71.00	116.02	130386114	99.15	0.09	10.50	63.95	191.00	204.78
H041	control	72.00	89.98	99849830	99.22	0.09	10.34	57.89	184.00	196.53
H041	tumor	69.73	116.96	132858860	99.19	0.09	9.67	63.90	190.00	203.7
H042	control	70.71	93.79	106388896	98.92	0.14	7.72	60.93	183.00	196.27
H042	tumor	71.52	114.81	129078252	98.95	0.10	9.83	62.26	185.00	198.04
H043	control	72.97	100.19	138794086	99.07	0.14	28.48	58.59	207.00	216.84
H043	tumor	71.85	127.07	158504598	99.28	0.13	19.17	62.11	210.00	221.33
H044	control	69.94	113.84	133946228	98.74	0.14	10.40	60.66	182.00	194.92
H044	tumor	69.52	88.35	104567892	98.93	0.16	10.04	62.23	187.00	199.99
H045	control	72.08	100.86	111607402	98.92	0.13	9.65	60.67	183.00	195.61
H045	tumor	70.61	143.39	165670464	98.57	0.14	11.96	60.66	178.00	191.67
H046	control	70.19	112.54	133052906	98.63	0.14	12.87	62.96	181.00	194.93
H046	tumor	69.41	88.19	102145380	98.42	0.17	10.56	88.94	189.00	213.89
H047	control	68.51	85.28	95420482	99.32	0.08	6.47	65.53	193.00	207.23
H047	tumor	71.87	131.27	145355380	99.04	0.21	9.64	61.27	192.00	205.06
H049	control	71.38	71.22	76278002	98.51	0.10	6.47	69.74	188.00	203.47
H049	tumor	71.46	83.56	87848078	98.77	0.08	4.92	65.45	187.00	200.87
H051	control	71.18	84.17	96721828	98.85	0.14	9.96	67.96	186.00	201.57
H051	tumor	69.68	109.64	128011726	98.75	0.17	8.90	58.63	182.00	193.83
H053	control	71.08	111.08	133102964	98.86	0.13	15.26	60.90	182.00	195
H053	tumor	68.93	116.21	135839818	98.35	0.19	10.39	84.25	188.00	210.68

H054	control	70.65	92.40	104186672	99.41	0.07	10.12	63.95	193.00	205.97
H054	tumor	70.24	110.65	123962400	99.17	0.09	9.17	61.88	187.00	200.66
H055	control	70.90	111.26	144145644	96.43	0.14	18.03	66.07	216.00	227.2
H055	tumor	70.48	102.43	123340268	97.01	0.15	12.17	68.23	217.00	229.37
H056	control	70.85	109.27	127198560	98.91	0.12	12.47	59.48	181.00	193.72
H056	tumor	69.80	110.50	128627662	98.64	0.16	11.39	63.25	184.00	197.64
H057	control	71.09	98.09	112871072	98.74	0.15	10.06	58.46	180.00	192.01
H057	tumor	70.47	114.05	133626156	98.69	0.17	10.37	60.80	183.00	196.14
H058	control	70.18	89.31	98395246	99.37	0.07	7.39	63.26	191.00	204.17
H058	tumor	70.91	119.45	132748940	99.14	0.10	9.33	59.51	185.00	197.57
H059	control	70.40	96.85	112277292	98.89	0.12	11.51	59.76	181.00	194.05
H059	tumor	69.91	116.14	131478638	98.80	0.13	9.13	63.79	184.00	198.24
H060	control	70.15	91.43	101641254	99.26	0.09	7.95	59.95	186.00	199.07
H060	tumor	69.50	113.82	129966040	99.16	0.09	9.80	67.45	193.00	207.6
H063	control	69.92	82.93	91464260	99.17	0.10	7.06	61.07	187.00	199.79
H063	tumor	72.43	128.94	138175638	99.13	0.21	7.30	64.65	200.00	214.15
H064	control	70.71	77.34	84438652	99.23	0.09	7.17	59.04	185.00	197.64
H064	tumor	71.83	137.88	153998672	99.04	0.21	10.39	65.73	200.00	214.25
H065	control	70.93	110.44	141403880	96.85	0.13	17.74	60.26	214.00	223.79
H065	tumor	71.30	127.12	155270820	96.65	0.14	14.00	63.16	218.00	228.4
H066	control	71.99	124.19	159446274	99.31	0.14	21.61	61.39	212.00	222.45
H066	tumor	71.25	127.88	157471276	99.17	0.14	17.27	64.85	217.00	228.2
H069	control	70.77	121.79	153330368	96.40	0.16	15.54	71.12	219.00	232.44
H069	tumor	70.51	107.83	137535898	96.31	0.14	16.20	65.83	215.00	227.02
H070	control	70.44	64.49	73661856	98.77	0.09	11.21	69.34	188.00	203.05
H070	tumor	71.28	84.94	91043384	98.64	0.09	6.63	71.19	189.00	204.44
H072	control	70.84	96.29	110370312	99.31	0.09	11.73	62.02	189.00	202.42
H072	tumor	69.99	116.27	133843720	99.17	0.09	11.22	69.18	196.00	211.03
H073	control	70.43	86.71	97272384	99.32	0.09	9.24	63.48	192.00	204.92
H073	tumor	70.55	136.31	152360788	99.15	0.09	9.47	61.43	186.00	199.35
H076	control	70.51	138.55	153438748	99.43	0.09	8.01	64.16	198.00	211.72
H076	tumor	68.46	172.41	198417054	99.12	0.13	8.60	58.91	191.00	203.07
H077	control	72.17	104.41	117815802	98.91	0.15	9.24	57.91	179.00	191.55
H077	tumor	70.37	102.86	115722266	98.70	0.13	8.12	59.79	180.00	193.09
H078	control	70.87	169.84	191180514	99.32	0.11	9.83	63.97	197.00	210.81
H078	tumor	70.96	118.76	132501532	99.25	0.12	9.12	60.42	195.00	207.42
H079	control	71.23	97.81	108232312	98.88	0.13	8.81	61.00	183.00	195.88
H079	tumor	70.73	97.45	110676532	98.87	0.10	9.09	61.48	181.00	194.76
H080	control	72.29	99.21	114493242	98.60	0.16	11.97	59.57	180.00	192.46
H080	tumor	70.73	122.05	140559358	98.73	0.17	9.30	57.23	179.00	191.46
H081	control	69.40	94.12	110488948	98.44	0.32	8.77	62.76	185.00	198.29
H081	tumor	68.96	126.34	140044902	99.00	0.14	5.53	64.08	188.00	201.83
H082	control	70.78	87.43	100619150	98.44	0.31	8.71	60.54	184.00	196.58
H082	tumor	70.57	144.66	166890730	99.05	0.15	11.45	63.25	188.00	201.64
H083	control	69.89	91.39	107234010	98.56	0.31	9.20	66.10	190.00	204.3
H083	tumor	71.05	122.49	133903788	99.31	0.09	7.63	63.56	190.00	203.5
H084	control	69.64	88.97	103190434	98.44	0.33	7.64	63.32	187.00	200.33
H084	tumor	70.52	139.81	153972640	99.14	0.11	7.45	61.03	187.00	199.59
H085	control	70.19	89.04	98516288	99.18	0.10	7.62	58.85	184.00	196.34
H085	tumor	72.45	133.84	148317308	99.01	0.22	10.47	62.59	193.00	205.67
H087	control	71.44	142.59	159930924	98.93	0.15	10.15	65.27	201.00	214.27
H087	tumor	73.03	133.75	144497896	99.10	0.14	8.88	60.40	194.00	206.16
H088	control	70.93	92.46	102501014	98.88	0.12	8.69	60.74	181.00	194.14
H088	tumor	70.82	116.66	140741950	99.04	0.12	13.31	64.20	189.00	202.47
H089	control	70.98	79.00	90773944	98.44	0.33	8.90	61.70	185.00	197.64
H089	tumor	72.11	114.86	129714704	99.31	0.09	12.08	61.47	187.00	199.89
H090	control	70.80	172.81	194968154	99.35	0.10	10.16	57.81	189.00	200.5
H090	tumor	71.71	154.01	175616632	99.11	0.14	12.10	63.08	198.00	211.25
H093	control	70.81	94.58	107239232	98.88	0.15	7.83	58.37	180.00	192.2
H093	tumor	70.92	110.83	124511960	98.98	0.10	8.98	63.66	185.00	199.05
H094	control	70.09	86.49	100193342	98.32	0.35	8.16	60.91	185.00	197.29
H094	tumor	71.51	88.65	96394840	99.35	0.08	8.04	63.73	190.00	203.3
H095	control	68.88	82.10	96288258	98.28	0.36	7.63	62.95	187.00	199.84
H095	tumor	70.35	140.86	156984358	99.26	0.09	8.71	61.35	188.00	200.75
H096	control	71.00	80.26	89143292	98.89	0.15	6.20	60.90	181.00	194.49
H096	tumor	70.87	120.64	139634652	98.71	0.14	10.45	60.14	181.00	193.88
H097	control	70.99	96.02	113270484	98.27	0.35	11.21	57.68	179.00	191.21
H097	tumor	72.03	111.47	122425280	99.30	0.08	9.72	60.12	186.00	198.59
H098	control	71.47	92.28	101286450	98.69	0.13	8.52	56.33	177.00	188.95
H098	tumor	70.15	117.66	136391188	98.90	0.10	10.58	62.41	185.00	198.43
H099	control	71.33	60.43	63712976	98.68	0.08	5.16	67.01	187.00	201.52
H099	tumor	70.56	128.69	140361002	98.64	0.09	6.99	65.77	186.00	199.67
H100	control	58.78	104.04	145699794	98.56	0.16	11.61	64.44	184.00	198.39
H100	tumor	71.57	134.78	161903948	98.73	0.13	16.54	61.16	181.00	194.19
H101	control	70.29	81.58	95314000	98.39	0.31	9.37	60.60	184.00	196.17
H101	tumor	71.15	143.80	160090178	99.39	0.08	9.39	60.33	187.00	200.21
H102	control	70.92	157.90	202408008	98.36	0.34	18.38	61.89	185.00	197.48
H102	tumor	70.57	122.06	134170722	99.39	0.07	7.50	61.16	189.00	201.52
H103	control	70.91	74.07	79017888	98.69	0.09	5.47	65.83	187.00	200.44
H103	tumor	69.38	95.17	103900056	98.49	0.10	5.39	71.54	188.00	204.34
H104	control	70.68	80.06	92487494	98.92	0.13	9.13	60.35	182.00	194.51
H104	tumor	71.26	109.92	129003728	98.98	0.12	12.29	63.89	187.00	200.85
H105	control	70.42	84.89	95010688	99.28	0.09	9.10	64.50	191.00	204.55
H105	tumor	72.55	116.27	126097680	99.16	0.21	8.58	63.62	201.00	213.84
H107	control	69.23	87.38	101800404	98.78	0.16	8.53	57.59	179.00	191.07
H107	tumor	71.34	110.18	130210444	98.58	0.19	12.31	57.16	178.00	190.27
H108	control	70.52	84.48	94762816	99.33	0.08	9.45	59.29	186.00	198.09
H108	tumor	70.05	133.54	152033424	99.08	0.10	10.32	62.02	188.00	200.9
H109	control	71.55	110.03	119080536	98.54	0.13	7.55	65.16	185.00	198.88
H109	tumor	70.10	78.36	83865042	98.62	0.09	4.76	67.86	186.00	200.89
H110	control	71.25	70.42	74931292	98.73	0.10	5.58	66.05	188.00	201.51

H110	tumor	69.38	83.24	89956984	98.49	0.09	4.64	67.87	186.00	200.78
H111	control	71.24	107.22	122900498	98.86	0.12	11.64	61.37	182.00	195.58
H111	tumor	72.20	113.56	130021700	99.39	0.07	13.35	61.85	190.00	202.74
H112	control	70.66	74.02	86057790	98.73	0.15	9.71	61.16	181.00	194.1
H112	tumor	70.14	104.61	123247630	98.97	0.14	10.19	60.82	184.00	196.55
H113	control	71.02	97.32	104875918	98.84	0.11	6.21	60.48	182.00	194.87
H113	tumor	71.31	104.67	113260346	99.37	0.08	7.29	60.29	187.00	199.37
H114	control	70.13	139.07	163797750	98.85	0.12	12.59	64.12	186.00	199.61
H114	tumor	70.89	129.29	151239260	98.73	0.13	13.38	60.88	182.00	194.89
H115	control	70.17	95.22	110971754	99.03	0.13	11.59	76.32	192.00	210.68
H115	tumor	70.73	115.48	125670208	99.24	0.09	7.00	59.83	183.00	195.72
H116	control	70.90	99.51	120809708	98.83	0.13	16.11	64.53	186.00	200.2
H116	tumor	71.87	107.06	117494334	99.30	0.08	9.40	59.50	185.00	197.7
H117	control	71.23	107.87	128685556	98.92	0.12	15.16	58.96	180.00	192.83
H117	tumor	70.34	120.91	136210442	98.89	0.11	9.46	63.53	186.00	199.39
H118	control	71.13	81.22	92306414	98.88	0.12	8.31	61.32	183.00	196.06
H118	tumor	70.26	120.09	138068768	98.74	0.14	8.93	60.78	183.00	195.52
H119	control	71.32	101.23	121427228	98.88	0.13	14.11	63.70	185.00	198.63
H119	tumor	70.68	116.72	140050528	98.56	0.20	12.65	58.80	178.00	190.97
H120	control	70.97	97.84	112435532	98.97	0.12	11.41	60.61	182.00	194.54
H120	tumor	70.32	127.82	153833542	98.85	0.14	15.05	63.18	184.00	197.77
H121	control	70.89	88.66	100204660	98.95	0.12	7.45	62.16	183.00	196.52
H121	tumor	70.73	122.38	141903496	98.69	0.16	9.40	57.21	178.00	189.98
H122	control	71.03	81.06	87991286	98.91	0.12	6.98	61.84	183.00	196.06
H122	tumor	70.20	89.83	103777386	99.03	0.10	10.39	64.24	187.00	200.87
H133	control	70.37	69.28	74335320	98.59	0.10	5.25	65.71	186.00	199.73
H133	tumor	70.91	96.89	104124174	98.64	0.09	6.29	66.23	186.00	200.49
H148	control	70.98	89.30	97623988	98.84	0.12	7.47	59.60	180.00	192.86
H148	tumor	71.00	107.46	119967166	98.74	0.11	7.87	58.73	178.00	191.15
H163	control	71.08	77.80	84193280	98.45	0.11	7.01	70.24	190.00	204.78
H163	tumor	70.18	89.62	96116130	98.81	0.09	5.16	69.19	189.00	204.16
H164	control	70.55	80.38	87446594	98.52	0.11	6.59	71.24	189.00	204.6
H164	tumor	68.85	100.05	110248782	98.70	0.09	5.82	72.57	192.00	207.7
H165	control	71.33	62.88	65874362	98.60	0.10	4.32	61.62	180.00	193.33
H165	tumor	70.88	80.98	86623174	98.80	0.09	5.91	69.32	190.00	204.96
H167	control	71.86	76.06	79810566	98.68	0.08	5.36	66.82	186.00	200.42
H167	tumor	69.93	102.84	112436480	98.75	0.09	6.48	69.28	189.00	204.33
H171	control	70.43	73.69	79161220	98.57	0.10	5.45	66.84	186.00	200.49
H171	tumor	69.78	115.41	126620474	98.80	0.09	6.57	66.44	187.00	201.65
H173	control	70.02	69.45	73848372	98.68	0.09	4.06	72.45	189.00	205.66
H173	tumor	71.15	86.47	94437086	98.79	0.09	8.08	69.99	191.00	205.9
H187	control	71.12	74.07	79239724	98.56	0.10	6.12	63.58	184.00	197.09
H187	tumor	70.40	96.02	102980058	98.63	0.11	5.29	66.35	187.00	201.17
H190	control	71.61	65.33	68670786	98.66	0.09	4.98	65.43	185.00	199.41
H190	tumor	70.18	83.05	89259284	98.72	0.08	5.48	67.72	189.00	203.14
H191	control	70.99	69.32	73755348	98.69	0.10	5.30	63.63	183.00	196.98
H191	tumor	69.46	86.04	93361880	98.73	0.08	5.36	66.01	186.00	200.4
H225	control	71.86	67.38	70664770	98.87	0.08	5.35	66.05	187.00	201.31
H225	tumor	70.17	76.14	81687484	98.79	0.08	5.38	79.13	194.00	212.35
H227	control	70.55	77.04	82575520	98.73	0.09	5.32	66.65	187.00	201.46
H227	tumor	70.16	79.33	85192968	98.69	0.09	5.05	69.00	188.00	203.38
H228	control	70.99	57.34	59536644	98.62	0.11	2.79	69.74	188.00	203.75
H228	tumor	69.60	128.35	142614566	98.69	0.09	7.48	69.66	190.00	204.8
H230	control	70.84	63.52	66900496	98.80	0.10	4.12	66.78	187.00	201.52
H230	tumor	71.28	102.04	110551048	98.82	0.08	7.49	68.64	189.00	204.13
H231	control	71.29	68.71	72443982	98.49	0.09	4.88	67.77	190.00	204.45
H231	tumor	70.05	107.95	118923772	98.75	0.09	7.33	68.52	189.00	203.78
H233	control	70.68	56.46	58899216	98.52	0.12	2.66	70.51	185.00	201.46
H233	tumor	69.12	101.09	111670474	98.76	0.09	6.26	73.37	192.00	208.61
H234	control	70.56	63.77	67709056	98.79	0.09	4.59	69.27	189.00	203.97
H234	tumor	71.45	97.08	104005544	98.66	0.09	6.66	66.81	186.00	200.51
H236	control	71.72	73.67	77321468	98.58	0.09	5.13	64.32	184.00	197.13
H236	tumor	70.22	94.12	101670542	98.68	0.10	5.76	67.51	187.00	201.95
H238	control	70.88	74.43	79133764	98.58	0.10	5.11	63.73	183.00	196.64
H238	tumor	69.34	93.97	102750964	98.70	0.10	5.69	68.30	189.00	203.21
H240	control	71.06	70.53	74679914	98.65	0.09	4.94	65.76	185.00	199.53
H240	tumor	71.00	97.66	106280466	98.81	0.09	7.61	70.91	191.00	206.5

Table S5: **Target profiling of AZD7762 and PF477736 in cell lysates of K562 cells.** Targets identified in kinobead assays for AZD7762 and PF477736 at 2 and 10 μM are shown. A target score of <0.5 indicates a good target specificity. The column BCR indicates if the protein was identified as a B-cell receptor responsive protein after IgM stimulation in Burkitt lymphoma cell lines [13].

Nb.	Gene	AZD7762-10	AZD7762-2	PF477736-10	PF477736-2	BCR
1	AAK1	0.25	0.39	0.23	0.23	No
2	ABL1	0.28	0.40	0.44	0.73	Yes
3	AURKA	0.89	0.92	0.48	0.65	
4	AURKB	0.50	0.52	0.43	0.52	No
5	AXL	0.45	0.68	0.50	0.68	
6	BCR	0.28	0.37	0.57	0.78	No
7	BMP2K	0.23	0.43	0.21	0.20	No
8	BRSK2	0.37	0.52	0.62	0.90	
9	CAMK2G	0.48	0.79	0.60	1.00	
10	CHEK1	0.28	0.26	0.29	0.26	Yes
12	CHEK2	0.17	0.19	0.24	0.43	No
13	CIT	0.56	0.80	0.40	0.44	Yes
14	CSK	0.19	0.32	0.66	0.93	No
15	EGFR	0.33	0.60	0.54	0.86	
16	EPHA7	0.28	0.54	0.77	0.93	
17	FER	0.24	0.35	0.88	1.07	No
18	FES	0.46	0.72	0.94	1.04	
19	FGFR2	0.87	1.02	0.44	0.65	
20	FLT4	0.48	0.52	0.55	0.61	
21	FRK	0.39	0.69	0.82	0.95	
22	FYN	0.28	0.47	0.46	0.79	No
23	GAK	0.23	0.31	0.21	0.25	No
24	HCK	0.28	0.39	0.52	0.69	Yes
25	IRAK4	0.25	0.40	0.56	0.88	No
26	KIAA0999	0.40	0.48	0.41	0.45	No
27	LIMK2	1.11	1.07	0.33	0.41	No
28	LOC100128443	0.94	0.95	0.36	0.21	No
29	LYN	0.33	0.53	0.40	0.58	Yes
30	MAP2K1	0.30	0.47	0.73	0.95	Yes
31	MAP2K2	0.22	0.43	0.63	0.84	Yes
32	MAP2K5	0.21	0.29	0.45	0.74	No
33	MAP3K11	0.43	0.46	0.63	0.83	No
34	MAP3K2	0.40	0.64	0.67	0.83	
35	MAP3K3	0.46	0.71	1.13	0.94	
36	MAP3K7	0.49	0.77	0.77	0.95	
37	MAP4K3	0.29	0.45	0.47	0.73	No
38	MAP4K4	0.25	0.29	0.58	0.87	No
39	MAP4K5	0.27	0.32	0.37	0.71	Yes
40	MAPK3	1.16	1.14	0.38	0.74	
41	MARK1	0.25	0.33	0.36	0.54	No
42	MARK2	0.23	0.35	0.39	0.54	Yes
44	MARK3	0.26	0.30	0.37	0.53	No
45	MARK4	0.34	0.55	0.17	0.24	No
46	MERTK	0.43	0.44	0.71	0.90	No
47	MINK1	0.37	0.39	0.55	0.80	No
48	MYLK3	0.18	0.26			No
49	PAK4	0.22	0.27	0.30	0.33	No
50	PDPK1	0.20	0.30	0.38	0.58	No
51	PKN2	0.34	0.52	0.36	0.61	
52	PRKAA1	0.33	0.52	0.41	0.66	
53	PRKAA2	0.42	0.65	0.58	0.85	
54	PRKCD	0.28	0.47	0.68	0.95	Yes
55	PRKG1	0.37	0.71	0.68	0.96	
56	PRKX	0.25	0.25	0.10	0.32	No

57	PTK2B	0.40	0.68	0.96	1.07	
58	RPS6KA4	0.54	0.70	0.45	0.69	
59	SIK2	0.28	0.33	0.27	0.34	No
60	SLK	0.27	0.44	0.73	0.92	Yes
61	SRC	0.25	0.35	0.50	0.83	No
62	STK10	0.27	0.41	0.78	0.94	Yes
63	STK17A	0.35	0.76	0.35	0.41	No
64	STK3	0.28	0.37	0.65	0.87	No
65	STK33	0.26	0.43	0.77	1.02	No
66	STK35	0.49	0.72			
67	STK4	0.20	0.21	0.65	0.94	No
68	SYK	0.30	0.46	0.70	0.92	Yes
69	TGFBR2	0.36	0.36	0.62	0.78	No
70	TNIK	0.37	0.41	0.41	0.70	No
71	TNK2	0.42	0.61	0.79	0.85	
72	YES1	0.38	0.64	0.38	0.75	
73	ACADM	0.40	0.39	1.00	1.03	No
74	FASN	0.23	0.24	1.27	0.99	Yes
75	IDH2	0.49	0.54			
76	ME1	0.25	0.28			No
77	PRDX2	0.44	0.49	1.25	1.07	No
78	SOD2	0.44	0.53	1.45	1.13	
79	CTSD	0.45	0.49	1.26	0.95	No
80	ANXA2	0.40	0.43	1.20	0.96	Yes
81	AP2A1	0.28	0.48	0.43	0.45	No
82	AP2A2	0.30	0.37	0.54	0.52	No
83	AP2B1	0.26	0.43	0.19	0.21	No
84	AP2M1	0.25	0.37	0.32	0.34	No
85	AP2S1	0.32	0.45	0.50	0.49	No
86	C20ORF3	0.39	0.40			No
87	CALML3	0.18	0.18			No
88	FDPS	0.42	0.45	1.19	1.02	No
89	GRB2	0.30	0.32	0.50	0.72	Yes
90	INCENP	0.24	0.35	0.41	0.47	No
91	LGALS7	0.18	0.17			No
92	MOBKL1B	1.03	0.98	0.45	0.71	
93	PHKA2	0.47	0.54			
94	PKP1	0.31	1.36	0.72	0.48	No
95	PRKAB1	0.23	0.40	0.31	0.61	No
96	PRKAB2	0.18	0.37	0.32	0.58	No
97	PRKAG1	0.27	0.47	0.36	0.64	No
98	PRKAG2	0.32	0.51	0.43	0.61	
99	S100A7	0.28	0.29	1.56	1.02	No
100	S100A8	0.16	0.24	5.77	1.04	No
101	S100A9	0.24	0.42	2.20	0.84	No
102	SERPINB12	0.69	2.30	0.62	0.48	No
103	YJ005	0.36	0.46	0.48	0.50	No
104	EXOSC6			0.18	0.21	No

Table S6: **Multivariate Cox regression model for overall survival with response to fludarabine as a covariate.**
The impact of response to fludarabine on overall survival was tested considering other important covariates. Complete case analysis was performed for $n = 156$ CLL patients (events: n=24).

	p-value	HR	lower 95% CI	upper 95% CI
age (per 10 years)	0.4	1.2	0.81	1.7
pretreatment	2.8×10^{-4}	9.2	2.8	31
trisomy 12	0.02	5.3	1.3	22
del(11)(q22.3)	0.78	1.2	0.38	3.6
del(17)(p13)	0.96	1	0.3	3.6
TP53	0.47	0.63	0.18	2.2
U-CLL	0.05	2.8	0.99	7.8
fludarabine (per 10% viability change)	0.09	0.8	0.57	1.1

Table S7: **Multivariate Cox regression model for overall survival with response to doxorubicin as a covariate.**
Similar to Table S6, but with doxorubicine instead of fludarabine as a covariate. Complete case analysis was performed for $n = 156$ CLL patients (events: n=24).

	p-value	HR	lower 95% CI	upper 95% CI
age (per 10 years)	0.12	1.4	0.92	2
pretreatment	1.8×10^{-4}	8.8	2.8	27
trisomy 12	0.01	6.1	1.5	25
del(11)(q22.3)	0.99	1	0.34	3
del(17)(p13)	0.92	1.1	0.3	3.8
TP53	0.72	0.81	0.26	2.5
U-CLL	0.04	2.9	1.1	8.1
doxorubicine (per 10% viability change)	0.03	0.52	0.28	0.95

Table S8: **Multivariate Cox regression model for time to treatment with response to ibrutinib as a covariate.**
The impact of response to ibrutinib on time to treatment was tested considering other important covariates. Complete case analysis was performed for $n = 152$ CLL patients, of whom 83 received treatment after sample collection. In addition to the main effect, also the interaction term between *IGHV* status and response to ibrutinib was tested (*IGHV*:ibrutinib).

	p-value	HR	lower 95% CI	upper 95% CI
age (per 10 years)	0.28	0.91	0.76	1.1
pretreatment	9.6×10^{-8}	0.22	0.13	0.39
trisomy 12	0.25	1.5	0.76	3
del(11)(q22.3)	0.78	0.91	0.48	1.7
del(17)(p13)	0.31	1.4	0.74	2.6
U-CLL	0.01	2.2	1.2	3.9
ibrutinib (per 10% viability change)	0.03	1.6	1.1	2.5
IGHV:ibrutinib	0.04	0.6	0.37	0.98

Table S9: **Multivariate Cox regression model for time to treatment with response to idelalisib as a covariate.**
Similar to Table S8, but with idelalisib instead of ibrutinib.

	p-value	HR	lower 95% CI	upper 95% CI
age (per 10 years)	0.30	0.91	0.76	1.1
pretreatment	3.4×10^{-8}	0.21	0.12	0.37
trisomy 12	0.21	1.5	0.78	3
del(11)(q22.3)	0.67	0.87	0.46	1.7
del(17)(p13)	0.31	1.4	0.74	2.6
U-CLL	8×10^{-3}	2.4	1.3	4.7
idelalisib (per 10% viability change)	0.04	1.6	1	2.6
IGHV:idelalisib	0.07	0.6	0.35	1.01

Table S10: **Multivariate Cox regression model for time to treatment with response to PRT62607 as a covariate.**
Similar to Table S8, but with PRT062607 instead of ibrutinib.

	p-value	HR	lower 95% CI	upper 95% CI
age (per 10 years)	0.4	0.93	0.78	1.1
pretreatment	2×10^{-8}	0.2	0.12	0.36
trisomy 12	0.4	1.4	0.67	2.7
del(11)(q22.3)	0.58	0.83	0.43	1.6
del(17)(p13)	0.42	1.3	0.69	2.4
U-CLL	4.5×10^{-3}	2.7	1.4	5.4
PRT062607 (per 10% viability change)	0.01	1.6	1.1	2.4
IGHV:PRT062607	0.02	0.58	0.37	0.91

References

[1] Michael Boutros, Lígia P Brás, and Wolfgang Huber. Analysis of cell-based rnai screens. *Genome Biology*, 7(7):R66, 2006.

[2] H. Li, B. Handsaker, A. Wysoker, T. Fennell, J. Ruan, N. Homer, G. Marth, G. Abecasis, and R. Durbin. The Sequence Alignment/Map format and SAMtools. *Bioinformatics*, 25(16):2078–2079, Aug 2009.

[3] K. Wang, M. Li, and H. Hakonarson. Annovar: functional annotation of genetic variants from high-throughput sequencing data. *Nucleic Acids Res*, 38(16):e164, 2010.

[4] A. Dobin, C. A. Davis, F. Schlesinger, J. Drenkow, C. Zaleski, S. Jha, P. Batut, M. Chaisson, and T. R. Gingeras. Star: ultrafast universal rna-seq aligner. *Bioinformatics*, 29(1):15–21, 2012.

[5] Simon Anders, Paul Theodor Pyl, and Wolfgang Huber. HTSeq – a Python framework to work with high-throughput sequencing data. *Bioinformatics*, 31(2):166–169, 2015.

[6] Michael I Love, Wolfgang Huber, and Simon Anders. Moderated estimation of fold change and dispersion for RNA-seq data with DESeq2. *Genome Biology*, 15(12):550, 2014.

[7] AB Olshen, ES Venkatraman, R Lucito, and M Wigler. Circular binary segmentation for the analysis of array-based dna copy number data. *Biostatistics*, 5:557–572, 2004.

[8] J. Staaf, D. Lindgren, J. Vallon-Christersson, A. Isaksson, H. Goransson, G. Juliusson, R. Rosenquist, M. Hoglund, A. Borg, and M. Ringner. Segmentation-based detection of allelic imbalance and loss-of-heterozygosity in cancer cells using whole genome snp arrays. *Genome Biology*, 9(9):R136, 2008.

[9] Seon-Young Kim and David J Volsky. PAGE: parametric analysis of gene set enrichment. *BMC Bioinformatics*, 6(5):73–85, 2005.

[10] Berthold Lausen, Rudolf Lerche, and Martin Schumacher. Maximally Selected Rank Statistics for Dose-Response Problems. *Biometrical Journal*, 44(2):131–147, 2002.

[11] Jerome H. Friedman, Trevor Hastie, and Rob Tibshirani. Regularization paths for generalized linear models via coordinate descent. *Journal of Statistical Software*, 33(1):1–22, 2 2010.

[12] Hui Zou. The Adaptive Lasso and Its Oracle Properties. *Journal of the American Statistical Association*, 101(476):1418–1429, 2006.

[13] Jasmin Corso, Kuan-Ting Pan, Roland Walter, Carmen Doebele, Sebastian Mohr, Hanibal Bohnenberger, Philipp Ströbel, Christof Lenz, Mikolaj Slabicki, Jennifer Hüllein, Federico Comoglio, Michael A. Rieger, Thorsten Zenz, Jürgen Wienands, Michael Engelke, Hubert Serve, Henning Urlaub, and Thomas Oellerich. Elucidation of tonic and activated B-cell receptor signaling in Burkitt's lymphoma provides insights into regulation of cell survival. *Proceedings of the National Academy of Sciences*, 113(20):5688–5693, 2016.
